# Biomarkers of meat and seafood intake: an extensive literature review

**DOI:** 10.1186/s12263-019-0656-4

**Published:** 2019-12-30

**Authors:** Cătălina Cuparencu, Giulia Praticó, Lieselot Y. Hemeryck, Pedapati S. C. Sri Harsha, Stefania Noerman, Caroline Rombouts, Muyao Xi, Lynn Vanhaecke, Kati Hanhineva, Lorraine Brennan, Lars O. Dragsted

**Affiliations:** 1 Department of Nutrition, Exercise and Sports, University of Copenhagen, Rolighedsvej 30, 1958 Frederiksberg C, Denmark; 2 Department of Veterinary Public Health & Food Safety, Ghent University, Salisburylaan 133, 9820 Merelbeke, Belgium; 3 School of Agriculture and Food Science, Institute of Food & Health, University College Dublin, Belfield 4, Dublin, Ireland; 4 Institute of Public Health and Clinical Nutrition, University of Eastern Finland, Yliopistonranta 1, 70210 Kuopio, Finland

**Keywords:** Biomarkers of food intake, Protein sources, Red meat, Processed meat, Poultry, Fish, Seafood

## Abstract

Meat, including fish and shellfish, represents a valuable constituent of most balanced diets. Consumption of different types of meat and fish has been associated with both beneficial and adverse health effects. While white meats and fish are generally associated with positive health outcomes, red and especially processed meats have been associated with colorectal cancer and other diseases*.* The contribution of these foods to the development or prevention of chronic diseases is still not fully elucidated. One of the main problems is the difficulty in properly evaluating meat intake, as the existing self-reporting tools for dietary assessment may be imprecise and therefore affected by systematic and random errors. Dietary biomarkers measured in biological fluids have been proposed as possible objective measurements of the actual intake of specific foods and as a support for classical assessment methods. Good biomarkers for meat intake should reflect total dietary intake of meat, independent of source or processing and should be able to differentiate meat consumption from that of other protein-rich foods; alternatively, meat intake biomarkers should be specific to each of the different meat sources (e.g., red vs. white; fish, bird, or mammal) and/or cooking methods. In this paper, we present a systematic investigation of the scientific literature while providing a comprehensive overview of the possible biomarker(s) for the intake of different types of meat, including fish and shellfish, and processed and heated meats according to published guidelines for biomarker reviews (BFIrev). The most promising biomarkers are further validated for their usefulness for dietary assessment by published validation criteria.

## Background

Meat, including fish and shellfish, represents a valuable constituent of a balanced omnivorous diet. The importance of meat from a nutritional point of view is related to its high-quality protein content, as it comprises a balanced source of all essential amino acids for muscle maintenance [1]. Minerals and vitamins, such as iron and B_12_ vitamin, and other micronutrients that are essential for growth and development, are additionally highly bioavailable from meat compared to other sources [2]. Meat processing, such as curing, smoking, or heating, further improves its organoleptic properties, as well as its microbiological safety and shelf life [3]. On the other hand, high consumption of processed meat and possibly red meat has been associated with a series of adverse health outcomes, including cardiovascular disease (CVD) [4], overall mortality [5], and certain types of cancer, especially colorectal cancer [6–8]. However, recent large, prospective investigation including almost half a million subjects from ten European countries [9], only found significant associations with processed meat intake. Poultry was not related to all-cause mortality, whereas red meat was related to higher all-cause mortality only before correcting for potential confounding [9]. Additionally, high intake of red and processed meat has also been associated with an increased risk of developing type 2 diabetes, although major inconsistencies regarding consumption levels exist between studies [10–12]. Fish and shellfish intake has been associated with positive health outcomes, such as decreasing CVD risk [13] and possibly colorectal cancer [14, 15], while its role in preventing type 2 diabetes development is still unclear [16, 17]. Different outcomes in the studies may also associate with the type of fish consumed and the cooking methods [17]. Moreover, aquatic meat has proven to be one of the major dietary sources of contaminants that are potentially harmful to human health, such as methylmercury and arsenic [18, 19].

Since associations are always debatable in terms of cause and effect and weak associations like those between meat and chronic disease are always at a high risk of being affected by bias or confounding, it is important to find biomarkers able to objectively discriminate between different classes of meat, particularly red meat, white meat from poultry, white meat from fish or shellfish, and processed meats [20]. The existing self-reporting tools for dietary assessment, such as food frequency questionnaires (FFQ) and dietary records, are imprecise and can be affected by systematic and random errors, especially when a certain food is perceived as healthy or unhealthy [21]. Dietary biomarkers have been proposed as possible objective measurements of the actual specific food intakes or at least as a support for the classical assessment methods [22]. A good marker of overall meat intake should reflect total dietary meat intake differentiating any meat consumption from that of other protein-rich foods; additional markers should discriminate between different meat sources (e.g., red vs. white or between different species, e.g., pork vs. beef) and identify cooking methods. In this paper, we present the results of a systematic search of the scientific literature according to the BFIrev methodology [23] providing a comprehensive overview of the possible marker(s) for the intake of different meat types, including fish and shellfish. The most promising biomarkers are further validated according to a validation scheme previously proposed for biomarkers of food intake [24]. This review represents the continuation of the reviewing process for candidate intake biomarkers for various foods of animal origin initiated in a previous paper on dairy and egg biomarkers, as part of the FoodBAll project [25].

## Methods

### Selection of food groups

In order to obtain a good coverage of the different meat sources, meat was subdivided into fresh meat (e.g., overall meat intake, red meat, white meat), fish and fish oil, and other aquatic meat, e.g., various shellfish, processed meat products (cured and smoked), and offal or organ meats. Two particular groups, strongly heated (e.g., grilled) meat products and biomarkers for fish contaminants, were also investigated. A total of nine food groups were thus selected for reviewing their respective markers of intake. A systematic literature search was carried out separately for each food group as detailed below.

### Primary literature search

The reviewing process, including article search and selection, reviewing and reporting of the results, follows the guidelines previously proposed by the FoodBAll consortium to carry out an extensive literature search and evaluation of biomarkers for food intake (BFIs) [23].

In brief, original research papers and reviews were searched in at least two databases, among which PubMed, Scopus, and ISI Web of Knowledge, using combinations of the grouped search terms (biomarker* OR marker* OR metabolite* OR biokinetics OR biotransformation) AND (trial OR experiment OR study OR intervention OR cohort) AND (human* OR men OR women OR patient* OR volunteer* OR participant*) AND (urine OR plasma OR serum OR blood OR excretion) AND (intake OR meal OR diet OR ingestion OR consumption OR eating OR drink* OR administration), as reported in Additional file 1: Table S1, together with specific keywords related to each animal-derived food group (Additional file 1: Table S2). The fields used as default for each of the databases were [All Fields] for PubMed, [Article Title/ Abstract/ Keywords] for Scopus, and [Topic] for ISI Web of Science, respectively. For almost all food groups, the three databases were consulted. For “processed meat” and “offal meat,” the search was carried out only in PubMed and ISI Web of Knowledge. The literature search process was carried out between November and December 2015 and updated at the end of December 2018 for all food groups.

The search was limited to papers written in English, while no restriction was applied regarding publication date. The research papers identifying or using potential biomarkers of intake for the different kinds of meat were selected from the list of retrieved references by one or more skilled researchers in a process outlined in Additional file 1: Figure S1. The papers obtained from the search in different databases were merged and filtered for duplicates. Subsequently, papers were screened based on title and abstract. The selected papers were then retrieved and assessed for eligibility based on the contents of the whole manuscript. Additional papers were identified from reference lists in these papers and from reviews or book chapters identified through the search. The result was a list of compounds potentially relevant as biomarkers for the food group and corresponding references.

### Secondary search, marker identification, and classification

A second search step was used to evaluate the apparent specificity of the markers in the list. The compound databases HMDB [26] and FooDB (http://foodb.ca/)) were used for a first evaluation of marker specificity. If the marker was not specific for a single food group, it was noted whether it was related to any food outside the meat food groups. In the latter case, unless levels were reported to be very low elsewhere, the compound was omitted from the list for the next search. The remaining list of putative biomarkers was used for a second literature search in the three bibliographic databases used also for the primary search. This was done to potentially identify other foods containing the putative biomarkers or their precursors as well as foods otherwise associated with these compounds. For the second web-search, we used the chemical name(s) of the marker as keyword, together with AND (biomarker* OR marker* OR metabolite* OR biokinetics OR biotransformation). Further filters, such as (urine OR plasma OR serum OR blood OR excretion) AND (intake OR meal OR diet OR ingestion OR consumption OR eating OR drink* OR administration) AND (human* OR men OR women OR patient* OR volunteer* OR participant* OR subject*), were added based on the results obtained. Again, markers not related to meat intake were deleted from the list. The remaining putative markers with potential specificity for each food group or for several combined animal food groups were finally listed in Additional file 2: Table S3, together with information about the study designs of the papers reporting their use. Due to the high number of results on marine fatty acids from the search on “fish and fish oil,” only the most representative among the observational studies having the highest number of participants were kept in Additional file 2: Table S3. A summary of the markers considered for further validation according to the validation guidelines [24] is reported in Additional file 3: Table S4.

### Marker validation

In order to further assess the validity of the candidate biomarker, the scoring system for biomarkers of food intake was used as described previously [24]. Briefly, the usefulness of each selected marker in Additional file 3: Table S4 has been established by answering a set of simple questions reflecting the biological and analytical criteria that a biomarker should fulfill in order to be considered valid. The questions have been answered for the most promising biomarkers. Possible answers were Y (yes), N (no), or U (unknown or uncertain). Each candidate marker was evaluated for plausibility (question 1), meaning that it is considered a plausible BFI for the food or food group based on food chemistry; dose-response relationship between quantity of food ingested and biomarker response (question 2); kinetics of immediate postprandial response (question 3a) and/or of repeated intakes (question 3b); robustness in complex diets or real-life exposure situations (question 4); reliability, i.e., concordance with other established measures of intake for the food or food group in question (question 5); analytical aspects, including the chemical stability of the marker (question 6), its analytical performance (question 7); and reproducibility in different labs (question 8) [24].

## Results

### General biomarkers of meat intake

General biomarkers of meat intake are common to all or a large number of foods investigated in this review. The search for biomarkers of meat intake provided 953 hits after removal of duplicates, resulting in the final selection of 20 papers from the web-search (Additional file 1: Figure S1). From the analysis of the reference lists and from the secondary search, another six papers were included in the review, resulting in a total of 26 papers; several relevant papers were dealing with specific meat subgroups and were therefore moved to this heading after reading the full text papers (Additional file 2: Table S3). The main markers associated with the intake of animal protein from meats were various isotope ratios, anserine, carnosine, 1- and 3-methylhistidine (MH), creatine, creatinine, carnitine and acylcarnitines, taurine, trimethylamine oxide (TMAO), and several unidentified features specified by their m/z ratio. In addition, urinary nitrogen was used for total protein intake assessment in many studies but the marker is not a specific meat intake biomarker and therefore not included. The main human sample investigated in the studies was urine, followed by plasma, although feces and hair were also used occasionally. Both interventional and observational studies were considered for the evaluation of the putative BFIs for meat intake.

#### Histidine-related compounds

Vertebrate muscles contain a large amount of dipeptides containing histidine, such as carnosine (*β*-Alanyl-l-histidine), anserine (*β*-Alanyl-3-methyl-l-histidine), and balenine (*β*-Alanyl-1-methyl-histidine); therefore, the quantification in human bio-specimens of such compounds or products of their catabolism, such as *β*-alanine, 1-MH (N^τ^-MH) and 3-MH (N^π^-MH), may support the assessment of meat intake [20]. Some degree of confusion exists regarding the nomenclature of 1-MH and 3-MH in regards to the numbering of the nitrogen in the imidazole ring of the histidine moiety because chemists and biochemists have used opposite designations for the two molecules; for instance, PubChem lists both compounds with both names and with both synonyms. We use here the chemical nomenclature, where N^π^-MH (π for nearest to the side chain) is termed 3-methylhistidine, while the common form in human muscle, N^τ^-MH (t for far from the side chain) is termed, 1-methylhistidine, as recommended in the IUPAC definition and in the major compound databases [26, 27]. The synonym of the dipeptide anserine is thus *β*-Alanyl-3-methyl-l-histidine (Mora et al. 2007; HMDB metabocard for anserine (HMDB00194)); however, while some older papers have termed it *β*-Alanyl-1-methyl-l-histidine, in this review we have reported the results from these papers with the IUPAC name [28, 29]. The dipeptide anserine, common in poultry, undergoes cleavage to give rise to 3-MH. The dipeptide balenine, common in some whales, cleaves to form 1-MH [30].

The content of histidine-containing dipeptides in food is highly variable [31–33] and their use as markers for meat intake may therefore be better at the group level than at the individual level. Carnosine contents are higher in beef and pork, while they are slightly lower in poultry [31, 33] and almost absent in fish, with the exception of some species of Anguilloidei (eel) [32]. Moreover, this compound was not detected in other foods of animal origin, such as milk [34] and may only be found in some sources of liver [35, 36], suggesting that the compound could represent a putative marker to assess the intake of terrestrial meat. Carnosine levels were found to be markedly increased in urine after the ingestion of beef, pork, chicken, and eel [31, 34, 37, 38], showing also dose-response [34]. Cheung et al. [38] further compared several meat sources and reported that urinary carnosine associates with meat intake in a selected group of subjects from the European Prospective Investigation into Cancer and Nutrition (EPIC). Carnosine levels were significantly higher in the urine samples from subjects who consumed chicken, red meat, and processed meat compared to those consuming fish, with no significant differences observed between subjects consuming similar amounts of the three types of meat. Carnosine is completely excreted in urine within 20–25 h following the meal, reaching a peak after 5 h [31]. This kinetic behavior makes the compound suitable for quantification in 24 h urines to estimate total intake of terrestrial muscle meat.

The results regarding the detection of carnosine in plasma are contradictory. Parker et al. [39] observed a peak in plasma at 2.5 h after beef consumption returning to background levels after 5.5 h, while other studies failed to confirm this [37, 38]. In a cross-sectional study of 294 Bavarian men and women completing 24-h recalls and providing non-fasting samples, plasma carnosine was associated with intake levels for all meat, red meat, and beef + pork but not for poultry, fish, or dairy [40] with approximately similar intake levels. These discrepancies could therefore be ascribed to a possibly weak performance of this marker, even at the group level. Plasma carnosine needs to be further investigated in carefully controlled trials to evaluate its usefulness as a BFI. With the current evidence, plasma carnosine is not a likely candidate marker for meat intake.

Anserine is present in the muscles of different non-human vertebrates, with poultry, rabbit, tuna, plaice, and salmon having generally higher contents than other marine foods, beef, or pork [31–33, 41]. An increase of urinary anserine excretion was found in humans after the consumption of chicken [31, 37, 38], rabbit [41], and tuna [31] and has been associated with intake of chicken [38], salmon [42], and, to a lesser extent, beef [34], while other observational evidence indicates no increase with fish intake [38]. In particular, anserine in urine was a good marker for chicken intake based on FFQ data in a selected sample of high vs. non-consumers from the EPIC study [38].

In a cross-sectional study from Bavaria, plasma anserine was associated in a dose-response manner with 24-h recall information for total meat, beef + pork, turkey, processed meat, and total dairy but not with chicken, total poultry, “seafood,” or beef or pork, individually [40].

In the human body, anserine is a substrate for carnosinase and is mainly degraded and excreted as 3-MH [31]. Endogenous formation of 3-MH is minimal in humans; therefore, plasma and urinary 3-MH are primarily associated with food intake. A significant increase in the level of urinary 3-MH was observed after consumption of red meat [34, 43, 44], chicken or poultry [31, 34, 38, 44–46], and fish [31, 38, 42, 44, 47], with a clear dose- or time-response relationship between urinary 3-MH excretion and the intake of the different animal proteins [38, 43, 44, 46]. Urinary 3-MH was able to discriminate subjects with vegetarian or omnivorous diets in controlled settings [43] as well as in free living subjects [48], suggesting that this marker could provide evidence of overall habitual meat intake. Importantly, the excretion of 3-MH depends on meat source. In studies reflecting recent intake, poultry intake gave higher levels of 3-MH when compared to those following consumption of pork and/or beef, most likely due to anserine breakdown [34, 38, 44]. The discrimination between poultry and fish intake is more uncertain, as fish species vary significantly in anserine and 3-MH content [32, 44], but chicken meat seems to have generally higher levels than fish. An increased level of 3-MH was reported after consumption of chicken compared to that of cod [38]. In one observational study from the Adventist Health Study-2 cohort (*n* = 1011 subjects), urinary 3-MH was positively associated with the intake of animal protein, red meat, and poultry, assessed both by FFQ and 24-h recall, while the correlation tended to be lower with fish, probably due to the preference for meat consumption in the population investigated [49]. On the other hand, Cheung et al. [38] observed higher concentration of 3-MH in the urine of a subset of EPIC subjects who consumed fish (mainly cod and haddock) compared to those consuming red and processed meat. Also in this case, poultry intake gave the highest amount of 3-MH excretion. 3-MH peaks in urine around 5 h after intake and decreases to baseline levels after ~ 40 h [31] or 48 h [50]. Sjolin et al. [44] reported an excretion half-life of 11.7 h for 3-MH, indicating that excretion would not be complete in a 24-h urine collection. In a recent study, ten healthy subjects ingested increasing amounts of chicken daily for 3 days in three consecutive weeks. Peak postprandial plasma levels on day 3 and fasting levels in urine and plasma on day 4 in each week increased with dose [46] corroborating accumulation of 3-MH with daily intakes. 3-MH was also detected in plasma samples after the intake of chicken and to a lesser extent after the consumption of fish in another controlled intervention study [38]. A much smaller increase was also observed after intake of beef and processed meat. Urinary and plasma 3-MH therefore represent possible markers to assess meat intake, but due to the differences between meat sources with higher levels observed after intake of chicken and some species of fish, the marker should be carefully evaluated for different study populations, based on their food preferences.

1-MH is a constituent of actin and myosin, the contractile proteins of skeletal muscles, and its urinary excretion is rapid following muscle protein breakdown [51]. This compound has been observed to significantly increase in urine after meat consumption [43, 52–54]. A single meal with ^15^N-labeled beef increased ^15^N-labeled 1-MH in urine at 0–8 h, confirming that food-derived 1-MH is excreted in urine [55]. Unlike 3-MH, the excretion of 1-MH increased during 72 h of fasting indicating catabolism of muscle tissue [56]. Daily meat intake in omnivores associates with 1-MH excretion, however with much more variation compared to 3-MH [34, 44], although with a similar postprandial kinetics and a half-life of 12.6 h [44]. Endogenous production causes a high inter-individual variability making this marker less suitable to asses meat intake by itself [43]. In one observational study, urinary 1-MH after meat and fish intake was not different between high and non-consumers [38]. Plasma 1-MH was not associated with any group of meat, poultry, or fish intake in a study based on 24-h recalls [40] and did not increase after a meal intervention with chicken breast [50].

As a consequence of the breakdown of histidine-containing dipeptides, β-alanine can also be observed in body fluids [41]. So far, only one metabolomics study reported an increase in plasma levels of this compound after beef consumption [57]. In an early study, β-alanine appeared to increase in human urine only after an anserine containing meal [41], but since β-alanine can also be formed endogenously by catabolism of pyrimidines [26], it may not be a robust marker to assess meat intake; further investigations are necessary to evaluate its specificity and sensitivity in a cross-sectional study.

#### Carnitine and acylcarnitines

Carnitine represents an essential cofactor in fatty acid metabolism in mammals, as it transports fatty acids under the form of acylcarnitines into the mitochondria of muscle cells for cellular energy production by lipid β-oxidation [58]. Carnitine may be synthetized in the body from the essential amino acids lysine and methionine. Most of the carnitine in omnivores comes from dietary sources, particularly beef and lamb [20]. Carnitine and acetylcarnitine were higher in the urine [59] and serum [60] of subjects consuming high-meat diets compared to vegetarians, and their urinary values were representative of habitual intake of red and processed meat in free-living subjects [61, 62]. Three acylcarnitines (acetylcarnitine, propionylcarnitine and 2-methylbutylcarnitine) were significantly higher in the urine of subjects from the EPIC cohort after the intake of meat and fish compared to control subjects with no meat intake, with no distinction among the different animal protein sources [38]. Propionylcarnitine and acetylcarnitine were also observed in plasma after a dietary intervention with meat and fish from the same research group [38]. Acetylcarnitine had slower kinetics than propionylcarnitine, reaching the highest value after 24 h when propionylcarnitine had already returned to the baseline level. In the EPIC-Oxford cohort, a series of acylcarnitines were also characteristic of the dietary pattern associated with high meat intake [63]. In particular, the concentrations of carnitine and acylcarnitines C-4 and C-5 were highest in meat eaters, followed by fish eaters, vegetarians, and vegans. Furthermore, C-3 and C-16 were higher in meat eaters and lower in vegans. Carnitine and acylcarnitines may reflect the intake of highly accessible amino acids and fatty acids contained in meat and fish and may be considered generic markers of intake of foods of animal origin. However, given that physiological conditions such as age, gender, and health status, as well as the intake of other foods with highly accessible amino acids or fatty acids may affect plasma acylcarnitine levels and their excretion into urine [64, 65], these metabolites may not be suitable markers per se for specific and quantitative assessment of meat intake and are therefore not considered for further validation as single markers.

#### Creatine and creatinine

Creatine is present in animal muscles, mainly in the form of phosphocreatine, and can be synthesized endogenously from arginine, glycine, and methionine. The main dietary source is meat, including red meat, fish, and poultry [66]. Several studies reported an increase in creatine levels after meat intake in urine [59, 62, 67, 68], erythrocytes [60], plasma, and serum [60, 69, 70]. Even a single meal containing meat has been shown to increase creatine in urine [71]. Associations between urinary creatine and habitual intake of shellfish [62] and oily fish such as salmon [42] have also been reported. Pallister et al. [69] proposed circulating levels of creatine as a possible marker of habitual intake of red meat and poultry, as observed in the UK Twin cohort study (*n* = 3559 subjects). However, experimental studies supporting this observation are lacking. Creatine in urine and plasma/serum are therefore candidate markers of total meat intake.

Creatinine can originate both from spontaneous decomposition of creatine and creatine phosphate in the human body and to some extent also during cooking of meat [72]. An effect of diet on urinary creatinine was observed in a study comparing omnivores and vegetarians, being higher in the former group [60]. Cross et al. [43] showed that urinary creatinine performed as a food intake biomarker only for extreme levels of meat intake. Creatinine excretion mainly reflects endogenous production. The excretion into urine is highly regulated in the body and is proportional to the total content of creatine and creatine phosphate, i.e., the total muscle mass, in individuals with normal kidney function [20]. Therefore, creatinine cannot be used to estimate intake of meat without correcting for muscle mass and the compound is consequently not generally useful as a meat intake biomarker.

#### Taurine

Taurine is the most abundant free amino acid in animal tissues and mainly derives from the ingestion of many different foods of animal origin including eggs and dairy, while it can also be synthetized endogenously [73]. Metabolomics studies reported higher urinary taurine in omnivorous subjects compared to vegetarians [59, 74]. In two intervention studies, taurine excretion increased with animal protein intake but the change was not significant for low levels of meat intake [43, 75]. Taurine also associated with shellfish intake in an observational study (*n* = 253 subjects), but not with general meat intake [62]. The marker does not appear robust enough to assess total meat intake.

#### Trans-4-hydroxyproline

Hydroxyproline (Hyp) is a post-translationally modified amino acid that represents a major component in protein collagen and elastin, and its main dietary source comes from animal foods rich in connective tissue [76]. This compound was found in plasma after the intake of beef [57], while Pallister et al. [69, 70] observed a significant correlation between the level of this compound in fasting blood and the frequency of meat and processed meat consumption in the UK Twin cohort. High levels of collagen have also been reported in fish [77], therefore it can be speculated that this marker may better reflect the overall intake of meat rich in collagen, rather than that of red or processed meat. The dipeptide prolyl-hydroxyproline (Pro-Hyp) has been reported as a marker of a healthy diet rich in fish [78]. Since there is also collagen in offal meat, HyP in plasma is a potential intake marker of total meat intake. Further studies of the contribution of endogenously formed HyP to its urinary excretion is needed as well as studies on HyP containing dipeptides in plasma and urine to potentially discriminate between endogenous and dietary sources.

#### δ15N and δ13C

Recently, the ratios of naturally occurring stable isotopes of carbon (^13^C/^12^C ratio, expressed as δ13C) and of nitrogen (^15^N/^14^N ratio, expressed as δ15N) in biological fluids and tissues have been proposed as novel nutritional biomarkers of meat and fish intake. Such an approach was originally used in archeology and recently introduced also in nutritional science to support dietary assessment of animal proteins [79]. When animals consume plants, they incorporate carbon and nitrogen from plants into their own tissues. The abundance of δ13C depends on the photosynthetic process utilized by the plants consumed by the animals; hence, animal feeding represents the primary determinant of variation in animal δ13C values [79]. In particular, C4 plants (crabgrass, sugarcane, and corn) have δ13C values 12–13‰ higher than C3 plants (wheat, rice, and the majority of fruit and vegetables). Incorporation of ^15^N depends also on dietary sources. In particular, animals preferentially excrete ^14^N as waste nitrogen, leading to δ15N values that are 3‰ to 4‰ higher than in their diet [79]. Moreover, ^15^N abundance of tissue proteins increases in the food chain. As a consequence, animal-derived food proteins are expected to have a higher amount of ^15^N compared to plant-derived protein food. In particular, the δ15N value has been suggested as a particularly good marker for aquatic meat consumption, as commonly consumed fish, such as tuna, salmon, and cod, are predators and reside at the higher levels of the trophic chain [80].

In a randomized 4 × 8 days cross-over study, Kuhnle and coworkers [81] observed significantly different isotope ratios among different diets (meat, fish, half-meat–half-fish, and vegetarian) in feces and urine samples, but not in blood samples, possibly due to the slower protein turnover. Higher δ13C and δ15N were observed in urine and fecal samples following a fish diet, and lower following a vegetarian diet, where protein had been substituted by carbohydrates. It was not possible to distinguish between meat and half-meat–half-fish diets in any of the biofluids. The higher level of δ13C and δ15N after the fish diet may be ascribed to the high level of fish that the subjects consumed during the intervention period. These results suggest that urinary and fecal δ13C and δ15N may be suitable biomarkers to assess short-term meat and fish intake, while blood levels may reflect longer-term intake. This interpretation is supported by data from Patel et al. [82], who were able to distinguish vegetarian from non-vegetarian subjects by serum δ13C and δ15N abundance in a sub-cohort within the EPIC-Norfolk study (*n* = 1254). Serum δ13C and δ15N were positively associated (*P* < 0.001) with fish protein intake (assessed by FFQ) and increased with increased fish consumption, while animal proteins, including dairy and meat proteins, were significantly associated with δ15N. Serum δ15N increased also with total animal protein consumption. Overall fish intake is more clearly associated with the isotope ratios in populations with high fish intakes while the ratios cannot distinguish different sources of intake in populations with mixed diets of aquatic and terrestrial meats [80, 83–85].

To monitor the habitual intake of animal proteins, δ15N and δ13C were measured in hair and compared with the data collected from FFQ and dietary records in observational studies [86–89]. This is possible as hair keratin is not recycled in the body, and allows a reliable recording of dietary habits during the past months [86]. Overall δ15N and δ13C in hair are positively associated with total meat intake and no specific meat group seems to be dominant, except when either fish or terrestrial meats dominate the diet [86, 90].

In conclusion, δ15N alone or in combination with δ13C represent promising markers to assess animal protein as well as meat intake, both for short-term based on urine or feces and habitual intake based on blood and hair. As these ratios are affected by local diets and agriculture, they should be validated for each population before being applied.

#### TMAO

The intake of red meat has been mentioned as a cause of increased plasma or urine levels of TMAO in some observational studies [91, 92], while increased levels are characteristic of fish or shellfish in intervention studies. In cross-over randomized controlled trials (RCTs) comparing meals with beef, egg, fruit, and cod, urine or plasma levels increased only with cod [38, 93–95]. Plasma TMAO, trimethylamine (TMA), and dimethylamine increased quickly after cod with a peak at 2 h. Beef and egg slightly increased postprandial plasma TMAO and the volunteers segregated into TMAO producers and non-producers, based on their microbiota [94]. In an intervention study comparing 4-week intakes of red meat, poultry, or non-meat proteins with levels of TMAO in plasma and urine, TMAO excretion as well as plasma levels increased only with red meat. A subset of 13 volunteers received isotope-labeled carnitine and betaine but only carnitine increased circulating isotope-labeled TMAO [96]. In conclusion, TMAO in urine does not seem to be a marker of recent red meat intake but is increased postprandially with certain types of fish (see section below). The microbial formation of TMA and its hepatic oxidation to TMAO increase plasma levels by 2–4-fold in subjects with a pre-disposing microbiota after diets rich in TMA precursors, including carnitine. However, plasma TMAO cannot serve as a marker of red meat intake because the response varies between individuals and may also depend on the efficiency of the kidney in removing it from the circulation.

### Mammalian (red) meat

Red meat includes muscle meat from mammals, although the amount of color (heme) varies considerably between muscle tissues from different species. Sometimes, the term pink meat is used to include pork, which has lower contents of heme iron compared to beef, veal, and mutton. However, we have included pork with the mammalian red meats here. Although some non-muscle mammalian meats (e.g., liver) and some fish (e.g., tuna) and poultry (e.g., duck) have red meat, they are usually not included with the term and also not included in this category here. The primary literature search for mammalian red meat in the online databases (Web Of Science, Pubmed and Scopus) resulted in a total of 1516 articles after the removal of duplicates. The exclusion of animal studies, studies on nutrition, physiology, and food composition based on title, abstract, or full text reduced the number of articles to 49. After the evaluation of marker specificity in online databases (HMDB and FooDB) and from additional literature, 26 putative biomarkers (lead, indole propionate, xylitol, ethyl glucuronide, methyl-α-glucopyranoside, sorbitol, cinnamoylglycine, and a range of lipid species) were excluded from entering the candidate biomarker list (Additional file 2: Table S3) because they can also be retrieved in foodstuffs other than mammalian meat. Based on references retrieved in full text or secondary sources, three additional articles were added. As a result, 19 papers were included containing information on 31 putative biomarkers (including ten unknown metabolites) related to red meat intake.

Acylcarnitines, carnitine, 3-dehydrocarnitine, anserine, β-alanine, 4-hydroxyproline, histidine, ^13^C/^12^C, ^15^N/^14^N, carnosine, creatine, 1-MH, and 3-MH have been found to increase after red meat consumption (Additional file 2: Table S3), but these compounds or their precursors are also associated with the overall intake of any meat and they are therefore not necessarily resulting from intake of red meat. The products of cooking of meat mentioned in this review are also unspecific with respect to the type of meat that is cooked. Therefore, these compounds are not suited as red meat-specific biomarkers and have been discussed in the appropriate sections on biomarkers of general, cooked, and processed meat intake.

#### Compounds potentially specific to red meat intake

Only three candidate markers, i.e., ferritin, apparent total N-nitroso compounds (ATNCs) (a term that encompasses nitrosothiols, nitrosyl iron, and other N-nitroso compounds (NOCs)), and 1,4-dihydroxynonane mercapturic acid (DHN-MA), were found as potentially red meat specific intake biomarkers. This is because these compounds may be formed at a significantly increased level as a consequence of the heme iron content of consumed meat.

Ferritin is a protein that carries and stores iron in the body, and its abundance in serum is thus related to the dietary intake of iron. Serum ferritin is more strongly associated with heme iron than non-heme iron or total iron intake [97]. However, the influence of other dietary constituents on iron absorption and serum ferritin concentration makes it difficult to use this protein as a biomarker for an individual’s red meat consumption and associations are not consistent in all studies [98–100]. Serum ferritin, therefore, does not seem to be a useful marker of individual red meat intake.

Heme iron is known to catalyze the formation of nitrite-derived NOCs associated with the formation of ATNCs. Heme iron also catalyzes formation of lipid peroxidation products (e.g., 4-hydroxy-2-nonenal (4-HNE) [101, 102]. The electrophilic nature of 4-HNE causes its conjugation with glutathione and this adduct is further metabolized into DHN-MA. Several intervention studies confirm the positive association between ATNCs and DHN-MA on the one hand, and heme iron content and/or increasing doses of red meat on the other hand [101, 103–105]. However, validation of DHN-MA as biomarker for red meat intake remains insufficient since only eight male volunteers were included in an intervention study for DHN-MA [105]. Additional information concerning the presence of DHN-MA in a larger and more representative study (e.g., including female test subjects as well) is needed. Furthermore, the DHN-MA precursor, 4-HNE, has previously been detected in foodstuffs other than red meat (e.g., in fish, edible oils, and fried foods [106]), limiting the specificity of DHN-MA as a red meat intake biomarker. For ATNCs, three independent intervention studies have been performed (encompassing a larger group of volunteers, including female test subjects), confirming the link between red meat intake and higher ATNCs levels in stool [101, 103, 104]. However, ATNCs have also been detected in nitrate or nitrite (as precursors of ATNCs) containing foodstuffs, e.g., cured meat (see section on “processed meat”), some cheeses, and beer [107, 108], questioning the use of ATNCs as a qualitative biomarker for red meat consumption. In addition, the analysis of ATNCs has been performed by a method involving thermal release of nitrosamines. In an earlier publication, this heating process has been shown to lead to artefactual formation of nitrosamines while no formation of any simple volatile nitrosamines was detectable [109]. In a study examining the factors affecting ATNCs, all meats, meat iron, and nitrate in the food were directly associated with their formation while vitamin C and total energy intake were inversely associated [110]. While ATNCs apparently associate strongly with red meat intake, the marker still needs validation by another independent and direct analysis of nitrosamines in human feces without the use of thermal sample treatments. In conclusion, there are currently no reliable candidate biomarkers of red meat intake.

### Offal meat

As reported in Additional file 1: Table S2, the search for offal meat biomarkers was restricted by the use of “NOT”-terms to limit the number of irrelevant papers (the search without these NOT-terms yielded a total number of > 100,000 papers). With the use of the selected NOT-terms, the search for offal meat biomarker papers yielded 647 papers in PubMed and 265 in Web of Science. After combination of the results and elimination of duplicates, the remaining number of papers was 671. Only a single relevant paper [105] includes two putative biomarkers for the intake of blood sausage (and liver paté) in rats and humans: DHN-MA and 8-iso-prostaglandin-F2alpha (8-iso-PGF2A), which are both known end products of lipid peroxidation.

Pierre et al. [105] report that urinary levels of 8-iso-PGF2A and DHN-MA increased in rats after the consumption of blood sausage. An increased excretion of DHN-MA, but not 8-iso-PGF2A, was subsequently observed in humans [105]. The results of this small-scale human study also show a trend toward an increase in DHN-MA after the consumption of liver paté, although this effect did not appear to be significant; both blood sausage and liver pate contain pre-formed 4-hydroxynonenal, which may have further complicated the interpretation. The authors ascribed the observed increase of 8-iso-PGF2A and DHN-MA to the high heme content of blood sausage, corroborating the potential use of the latter as a candidate heme intake biomarker. Consequently, any heme-related BFI would be likely to rise after the consumption of all meat products rich in heme-iron, including red meat, blood products, and liver. Neither 8-iso-PGF2A nor DHN-MA is therefore specifically related to the consumption of offal meat, although the latter may be a lipid peroxidation product more abundant after heme-rich food consumption, as described above. In conclusion, no candidate biomarkers of offal meat intake were identified in the literature search.

### Poultry

Poultry meat intake includes mainly farmed chicken, duck, goose, and turkey; however, wild fowl is expected to have similar markers. The term “white meat” is often used interchangeably to cover either poultry only, or to include poultry as well as fish and shellfish. As already mentioned, some poultry (e.g., duck and fowl) has reddish muscle and this is also the case for several species of larger fish, including tuna. In this review, we have included the intake markers for the different meats with their biological class of origin and “white” meats in this section include only poultry.

The literature search performed identified a total of 2311 articles from the three databases. This was reduced to 2055 articles after removal of duplicates. Sixteen articles were identified after screening on the basis of title and abstract. Exclusion criteria for the remaining 2026 articles included effects on physiology; effects on drug metabolism; articles related to antioxidant markers, disease/health markers, or oxidative stress markers; and other articles not relevant to biomarkers of poultry intake by humans, such as animal studies. Full texts of the 16 papers were downloaded and assessed further for exclusion/inclusion criteria. Exclusion criteria at this stage included inappropriate study design, studies on whole diets or on food preparation, and articles not specific to poultry meat intake. Eight articles were retained, focused on poultry products. Additional file 2: Table S3 provides a summary of remaining candidate biomarkers for poultry meat. Only studies on chicken or turkey intake were identified through this search process.

#### Anserine and methylhistidines

As previously mentioned, we identified a certain degree of confusion surrounding the nomenclature of 1-MH and 3-MH. Based on IUPAC nomenclature, 3-MH and not 1-MH is of interest with respect to poultry consumption [46]. Dietary intake of poultry meat is consistently associated with urinary excretion of 3-MH [44, 111], although a low endogenous source of this compound (3-MH in the correct nomenclature, 1-MH in the article by Giesecke et al.) may also exist in mammals, including humans [56], leading to a low level of background 3-MH excretion in all studies. Excretion of 3-MH in urine [38, 40] as well as peak postprandial plasma levels show dose-response within normal levels of chicken intake and plasma 3-MH is still measurable after overnight fasting [46]. The reported levels vary between quantitative studies [40, 46] indicating analytical issues or instability, and inter-laboratory comparisons are therefore needed.

The dipeptide, anserine, was reported to increase in both plasma (*C*_max_ = 2.72 ± 1.08 μM at 100 min) and urine samples after ingestion of chicken or chicken broth in healthy women (*n* = 4) [37]. Plasma anserine has been associated with poultry intake in a dose-response fashion and high levels were especially associated with self-reported ingestion of turkey in a cross-sectional study from Bavaria [37]. Urinary anserine was particularly associated with chicken intake in high versus non-consumers in a small subset of EPIC participants [38]; however, no information exists on its excretion kinetics.

Guanidinoacetate, a precursor compound in the biosynthesis of creatine found to increase muscle creatine and muscle growth in chicken [112], has been found by NMR analysis in urine as a result of chicken consumption [46]. The compound was found to associate strongly with chicken intake in an intervention study in the UK and this finding was confirmed in a cross-sectional study from Ireland [46]. Guanidinoacetate in urine discriminated intake of chicken from intake of other meat sources and reflected chicken intake in a dose-response fashion. In a dietary study with mixed dairy and meat proteins, including poultry, provided to Danish boys, guanidinoacetate was also observed by NMR analysis [113]. Further validation of the marker is needed from other countries and for other sources of poultry, in particular to determine if the excretion of this metabolite is in any way a consequence of animal feeds [114]. Up until then, this marker seems particularly promising as a unique chicken intake marker.

Several markers were also reported as being associated with cooking methods such as fried and grilled chicken. The putative markers PhIP (2-amino-1-methyl-6-phenylimidazo[4,5-*b*]pyridine), MeIQx (2-amino-3,8-dimethylimidazol4,5-*f*]quinoxaline), and their metabolites are extensively covered in the next section of this review. Similarly, metabolites related to polycyclic aromatic hydrocarbons (PAHs) detected after consumption of grilled chicken are also reported there. Although levels of these metabolites are increased following consumption of cooked chicken, they are also found in other cooked foods such as fish and beef [115, 116], and consequently lack specificity. In conclusion, 3-MH and anserine in urine and plasma should be considered as candidate biomarkers of poultry intake and guanidinoacetate in urine as a highly promising marker of chicken intake. Anserine, in particular, will largely benefit from information on its kinetic profile and dose-response. Overall, additional studies with other poultry meats are needed for further confirmation and validation of all three markers.

### Intake biomarkers for heated meat

Most meat is ingested after cooking; frying, broiling, baking, and grilling are commonly reported as the most popular cooking methods for meat products [117]; however, actual frequencies of different cooking practices are not often reported. In the search for dietary exposure markers for fried, broiled, or grilled meat, 391 unique papers were identified, resulting in 26 selected papers after the screening process and additionally 13 from the secondary search and reference lists (Additional file 1: Figure S1). A total of 39 putative markers for cooked meet have been identified. All compounds belong to the families of heterocyclic aromatic amines (HAAs) and PAHs. One study, based on untargeted metabolomics, reported a series of pyrraline derivatives which were positively associated with the heating process, but the contribution of heated meat could not be separated from those of other cooked foods [118]. Therefore, such compounds were disregarded in the final table. The final putative biomarkers are reported in Additional file 2: Table S3.

#### Heterocyclic aromatic amines

HAAs may represent specific markers for the intake of intensively cooked meat, as they are produced during high-temperature cooking of foods containing creatinine, creatine, and amino acids, which is the case only for muscle tissue. So far, more than 25 different heterocyclic aromatic amines have been found in roasted and fried meat and fish [119, 120]. Their content depends on the cooking method, but it has been shown that in real-life conditions the most abundant HAAs are PhIP and MeIQx [120, 121]. Only a small fraction of the HAAs can be found intact in urine, while the rest undergo biotransformation, including phase I metabolism by CYP1A2 and phase II metabolism by acetylation, glucuronidation, and sulphation. As a result, bio-fluids and tissues (e.g., urine, stool, blood, or hair) may contain a complex metabolic pattern reflecting the exposure to specific HAAs [122, 123]. The most reactive metabolites can also covalently bind DNA or proteins, producing adducts, which can be detected in DNA or tissue as well as in urine after DNA-repair or protein degradation [122, 124]. The use of adduct markers is limited due to the low abundance of these compounds and the invasive techniques sometimes required to collect the relevant tissue. Indeed, the majority of these investigations have been conducted on biopsy samples of patients that were obtained during clinical diagnosis of cancer [122]. However, some studies also used blood protein adducts to albumin or erythrocyte globin [124].

Several studies have examined possible markers related to acute HAA exposure in humans after the intake of roasted, fried, or grilled beef, chicken, and/or fish, focusing on the analysis of both free and conjugated HAAs in urine. The most investigated compounds so far are PhIP and MeIQx, which have been quantified before and after enzymatic hydrolysis [121, 125–127] and as their phase I and II metabolites [128–130] (Additional file 2: Table S3). These studies show that PhIP and MeIQx are mainly transformed through hydroxylation followed by conjugation, with glucuronidation as the major urinary excretion pathway in humans. The biotransformation products are completely excreted within 24 h after single intakes of roasted meat [128, 130]. After volunteers had consumed identical amounts of charbroiled beef for four consecutive days, the excretion of PhIP in enzymatically hydrolyzed urine decreased almost to baseline levels 48–72 h later, with a significant correlation between the urinary levels of PhIP and the amount of charbroiled meat consumed [125].

The levels of MeIQx in hydrolyzed urine were higher than those of PhIP [121, 126], suggesting a higher degree of direct conjugation of MeIQx, or a relatively greater excretion of metabolized PhIP through feces, as shown by Vanhaecke et al. [131]. When urine was not hydrolyzed, only PhIP was detected in urine after the intake of a single meal of roasted chicken, whereas unconjugated MeIQx and 4,8-DiMeIQx, which were found in the fried chicken, remained undetected [132]. Although the amounts of HAA metabolites measured in urine were highly associated with the ingested dose of MeIQx and PhIP, high inter- [116, 133] and intra-individual [128] variability in metabolism of the HAAs was observed. Notably, differences between “fast” and “slow” excretors as well as between men and women were observed [128, 130].

In observational studies, MeIQx has been found to be a more reliable urinary marker to assess consumption of highly cooked or processed meat. The urinary levels of MeIQx [134] and PhIP [135] have been compared among black, Asian (Chinese or Japanese), and white men, and correlated with the results of a food frequency questionnaire. MeIQx levels were positively associated with the intake frequency of bacon, pork/ham, and sausage/luncheon meats [134] while urinary PhIP was not associated with intake frequencies of any cooked meat, suggesting that self-administered dietary questionnaires may not properly describe cooked meat preferences, frequency of intake, and cooking methods [135], or alternatively that unknown sources of PhIP affect total excretion.

Further markers related with dietary HAA exposure are the PhIP hydroxylation metabolites. 4′-OH-PhIP (2-amino-1-methyl-6-(4′-hydroxyphenyl)imidazo[4,5-b]pyridine) has been proposed as detoxification product, derived from the oxidation by several CYPs of the aromatic ring system. The metabolite, 5-OH-PhIP (2-amino-1-methyl-6-(5-hydroxy)phenylimidazo[4,5-*b*]pyridine), has been suggested as a marker of activation of PhIP [136] since it represents a rearranged product of N-hydroxylation of the exocyclic amino group by cytochrome P450 (CYP1A2). In several studies [121, 132, 137], 4′-OH-PhIP was found to be the most abundant urinary hydroxylated metabolite after the consumption of cooked chicken. This compound is already abundantly present in the heated chicken meat and only a small amount (11% after enzymatic hydrolysis and 0.66–1.3% in untreated urine samples) is derived from human PhIP metabolism [132, 137]. This suggests that 4′-OH-PhIP may represent a good marker for heated meat intake, although studies showing its presence and kinetics of formation in other meats than chicken are needed in order to validate its plausibility as a general marker of any fried or grilled meat.

High inter-individual differences were observed for fecal excretion with no differences in the percentage of the PhIP dose excreted [131]. Vanhaecke et al. [131] examined the urinary and fecal excretion in humans of a newly identified microbial PhIP metabolite, PhIP-M1 (7-hydroxy-5-methyl-3-phenyl-6,7,8,9-tetrahydropyrido[3′,2′:4,5]imidazo[1,2-*a*]pyrimidin-5-ium chloride) after the consumption of cooked chicken, containing a known amount of PhIP. Urinary PhIP-M1 increased over time with a peak between 48 and 72 h, showing that microbial metabolism appears later in the excretion profile of body fluids compared to the parent compound. The percentage of recovered PhIP-M1 was quite high in feces, with high variability among subjects, probably associated with differences in diet, digestion, metabolism, and microbial composition. There was no difference in the percentage of the PhIP dose excreted as PhIP-M1, indicating apparent dose-response. In conclusion, this additional microbial PhIP metabolite, PhIP-M1, adds to the complex metabolism of PhIP and may further complicate the use of any PhIP metabolite for monitoring exposures to heated meats.

Other compounds besides PhIP and MeIQx have been identified in urine after the intake of roasted meat (Additional file 2: Table S3). These include 9*H*-pyrido[3,4-*b*]indole (norharman) [132, 138], IQ (2-Amino-3-methylimidazo[4,5-f]quinoline), Trp-P-2 (3-amino-1-methyl-5*H*-pyrido[3,4-*b*]indole), Trp-P-1 (3-amino-1,4-dimethyl-5*H*-pyrido[3,4-*b*]indole), and AαC (2-amino-9*H*-pyrido[2,3-*b*]indole) and harman [138]. However, these molecules were found to be less reliable as markers of exposure to heated meat, when compared to PhIP and MeIQx. Their presence in body fluids or tissue may not be entirely due to meat consumption, but also to smoking habits [139–141], endogenous production, and the consumption of other foods derived from heating or fermentation, such as alcoholic drinks and coffee [142].

Markers related to the intake of high-intensity cooked meat have been investigated also in human hair, providing an average of the exposure over a period of several months [143–150]. Analysis of hair samples showing dose-response, a known dependence on melanin levels and independence of hair dying, and CYP1A2 phenotype, has been shown for measurement of PhIP after fried beef intake for 3–4 weeks [143–145]. The lack of dependence on metabolism is in accordance with findings showing that CYP1A2 phenotype over a 20-fold activity span is a poor predictor of HAA metabolite excretion in urine [150]. Turesky et al. [149] examined the levels of PhIP accumulating in the hair closest to the scalp of 44 volunteers after a 4-week semi-controlled diet of cooked meat containing known quantities of PhIP. The study showed a good correlation between the level of PhIP in hair and the dose of PhIP ingested. Kobayashi et al. [144] compared PhIP levels in the hair of seven volunteers with the results from dietary records over 28 consecutive days. PhIP levels in hair were found to be highly associated with the grilled/stir-fried meat intake but not with the grilled/stir-fried fish intake. The marker, therefore, seems to be useful in very different populations in terms of food culture and genetics. No other HAAs can be measured in hair and no, or only marginal, PhIP could be measured in vegetarians. These studies point to PhIP in hair as a promising biomarker for assessment of average intakes of heated terrestrial meats but further validation studies are needed to assess stability of the samples and influence of hair structures.

In conclusion, the studies reported so far identified and quantified specific carcinogenic HAAs in urine, feces, and hair after consumption of well-done, heated meat, showing that there is a significant correlation between the intake and the excretion of these compounds. In particular, the most promising exposure markers seem to be total PhIP and MeIQx in urine and PhIP in hair. The complexity of absorption, metabolism, and distribution of these compounds leads to very high inter- and intra-individual variation for some of the blood and urine metabolites and these compounds may therefore only work reliably as markers for comparison of groups rather than individuals. PhIP in hair seems a consistent candidate biomarker to estimate intakes of fried or grilled terrestrial meat.

#### Polycyclic aromatic hydrocarbons

PAHs are mainly produced from the incomplete combustion of organic matter. Therefore, the main human exposure sources are associated with environmental factors, such as tobacco smokes, emission of vehicles, and occupational exposure [151]. Anyway, it has been observed that in non-smoking subjects without occupational exposure, the main source of PAHs is represented by food [152, 153]. PAHs are present both in uncooked and cooked food. In the former, these compounds may originate from environmental contamination from soil, air, or water [154]. Cooked food, particularly meat grilled directly over an open flame, grilled over charcoal, or smoked, may contain measurable amounts of PAHs arising from fat pyrolysis or from the adsorption onto foods of PAHs emitted from the combustion process [155]. The most abundant PAHs reported in barbequed or grilled meat were naphthalene (NAP), phenanthrene (PHE), fluorine, and pyrene [152, 156].

Urinary 1-hydroxypyrene (1-OHP) and 1-hydroxypyrene-O-glucuronide (1-OHPG), two metabolites of pyrene, are considered the most relevant biomarkers for assessing individual exposure to PAHs [157–160]. Even though they have been observed to increase following the consumption of barbecued meat, a very high inter-individual variability has been observed, suggesting that these metabolites may not be suitable markers for individual assessment of the intakes of grilled, broiled, or roasted meat [158–160]. Furthermore, it has been reported that the main source of urinary 1-OHPG is cigarette smoke, while car vehicle exhaust contributed almost to the same extent as the diet in non-smoking subjects [161]. A considerable amount of 1-OHPG excretion is also attributed in populations using mate tea while fried or grilled meat was not significantly associated with 1-OHPG [162]. Cooking methods (baking bread at home and the intensity of food frying), passive smoking, and genetic polymorphisms affecting hydroxylation and conjugation, additionally highly contributed to the excretion of 1-OHPG [163]. Therefore, the background levels for 1-OHPG would be too variable in a real exposure setting and the contribution of dietary intake of high-intensity heated meat could not be distinguished from environmental factors, even in subjects who never smoked.

Further studies identified monohydroxylated metabolites of NAP and PHE in urine as possible markers of short-term dietary intake of PAHs after consumption of fried beef and chicken, particularly 2-hydroxy-NAP and 1-, 2-, 3-, 4-, and 9-hydroxy-PHE [152, 156]. These metabolites demonstrated a high correlation with the parent compound present in food in a highly controlled setting where the environmental confounders were minimized. Even though these metabolites show a higher robustness in relation to the other environmental factors, the existence of other background source acting as potential confounders cannot be overruled. NAP and PHE are well-known environmental contaminants causing aquatic toxicity and they are therefore likely to show environmental bias similar to other PAH related markers [164].

In conclusion, none of the markers of PAH intake are currently promising markers of charcoal-grilled meat intake.

### Processed meat

The search for papers on processed (not including heat processing) meat biomarkers delivered 2522 papers via PubMed and 2560 papers from Web of Science. This rendered 3772 unique papers, of which 38 were included after screening of the titles and abstracts, whereas only six relevant papers remained after assessment of the full text. Exclusion was based on a lack of focus on the assessment of dietary intake in general, and processed meat intake in particular. Four additional papers were retrieved after the secondary web search. In total, 12 relevant papers were found, reporting putative markers such as MeIQx, 1-OHP, thiobarbituric acid reactive substances (TBARS), ATNCs, nitrosoproline, glycerophosphatidylcholine (PC) C38:4 (PC38:4), acetylcarnitine, carnitine, and lathosterol. Out of these, MeIQx and 1-OHP are also markers of grilled and fried meat; ATCN and DHN-MA are potential markers of heme-containing foods; carnitine and acyl-carnitines are candidate markers of meat intake in general; and lathosterol, TBARS, and PC38:4 are unspecific to processed meat intake. These compounds were therefore disregarded as candidate markers of processed meat. However, they may still be useful as part of combined markers for processed meat intake assessment. This leaves N-nitrosoproline as the only candidate biomarker for this specific meat subgroup.

#### N-nitrosoproline

Decades ago, Stich and co-workers [165] investigated the interfering role of the consumption of nitrite-preserved meats on urinary N-nitrosoproline concentrations. The results demonstrated that N-nitrosoproline increased significantly after the consumption of, e.g., tinned ham, pork luncheon meat, and different types of sausages, although N-nitrosoproline can also be formed endogenously in the gastro-intestinal tract. The authors were able to directly link N-nitrosoproline levels in different processed meat types to higher levels of N-nitrosoproline in human urine. Moreover, endogenous N-nitrosoproline formation appeared to be negligible in comparison with the levels formed after intake of cured meats. N-nitrosoproline therefore demonstrated potential to serve as a candidate quantitative biomarker for the intake of nitrite-preserved meat. Later on, other investigations showed that nitrite-preserved meat is not the sole or even the main potential contributor to elevated urinary N-nitrosoproline [166, 167] since tobacco use is another important source, eliminating N-nitrosoproline as a robust, specific biomarker for nitrite-preserved meat intake. However, other sources may be identified with other biomarkers and combinations of markers may therefore be developed to assess intake of cured meat besides other environmental or food sources. Further studies of N-nitrosoproline in complex exposure scenarios are therefore warranted.

#### Unidentified potential markers of processed meats

In studies by Playdon et al. (2016) and Cheung et al. (2017), the occurrence of five unidentified compounds was associated with the intake of processed meat [38, 62]. Additional research is required to identify these molecules and assess their relevance as markers of processed meat intake.

In conclusion, only a limited amount of original research papers document the search for a processed meat biomarker. More importantly, up until now, none were able to identify a biomarker that is specific for processed meat intake.

### Biomarkers of fish, fish oil, and other seafood intake

Three parallel searches of the scientific literature have been carried out for markers of aquatic meats and oils: one search addressed the identification of markers related to fish and fish oil intake, another on seafood, and a third search was focused on naturally occurring environmental toxicants related to seafood consumption (Additional file 1: Table S2).

A number of 1340 unique articles were identified for the search on fish and fish oil biomarkers of which 1115 articles were discarded in the first screening based on title and abstract. The main reasons for exclusion were effects on physiology, bone health and calcium metabolism, macular degeneration, effects on drug metabolism, as well as studies on effects in fish. Out of the 225 papers included in the second screening for which the full-text was assessed, 55 were kept and are included in Additional file 2: Table S3 together with 19 papers identified from the secondary searches. Papers that were discarded dealt with irrelevant diets (pure n-3 fatty acids, not fish oil), arachidonic acids, seal, astaxanthin from microbes, etc.), outcomes (effects on cholesterol levels, 1-carbon metabolism, immunology, fatty acid levels in breast milk, neurological development of newborns, urinary iodine excretion), or were excluded due to poorly reported methodology or inappropriate study designs for BFI discovery. One proteomics study unrelated to biomarker discovery and four studies for which the access links were unavailable were also excluded. Several additional studies arose from the literature search within the general meat section, while one cross-sectional and one intervention study were found through the targeted searches on TMAO and CMPF (3-carboxy-4-methyl-5-propyl-2-furanpropionic acid), adding a total of 15 papers to the final list (*n* = 86). Among the papers included in Additional file 2: Table S3, 45 are relevant for fish consumption, 34 for fish oil supplementation, and five investigate both fish and fish oil mixed intakes.

The literature search for shellfish resulted in 443 papers, of which 33 were kept after screening abstracts and titles. The reasons for exclusion were investigation of contaminants in seafood and their effects on health, effects on physiology of aquatic food consumption, and food allergies. After accurate examination of the text, the remaining papers included 19 mentioning shellfish, 13 on mixed intakes together with fish, and six only on shellfish, see Additional file 1: Figure S1.

For the search on natural environmental contaminants associated with the intake of aquatic meats, only arseno-compounds have been considered. The literature search resulted in 100 papers, which were reduced to 14 after screening abstracts and titles. Generic markers for inorganic arsenic exposure were excluded. Other reasons for exclusion within this category were irrelevant dietary intervention (e.g., arseno-sugar and cod liver) and a lack of firm conclusions in associating specific foods to the markers of interest. Only eight papers measuring organo-arsenic species in body fluids and tissues in relation to fish, mixed fish, or shellfish intakes were included and these papers are therefore listed under the fish, or mixed fish and shellfish categories.

In Additional file 2: Table S3, the studies on intake biomarkers of fish, fish oil, shellfish, and mixed exposures of these are listed in separate sections; however, due to the large overlap in the compounds identified as markers, the foods are all discussed under one heading here and subdivided by chemical class.

Several biomarkers have been found to be associated with intake of fish and shellfish as reported in Additional file 2: Table S3. The majority of the studies concern consumption of marine foods and derived products aimed to increase the levels of omega-3 (n-3) long-chain polyunsaturated fatty acids (n-3 LCPUFAs) in biofluids and tissues, while more recently suggested markers include furan fatty acids, e.g., CMPF, TMAO, 1,2,3,4-tetrahydro-*β*-carboline-3-carboxylic acid (THCC), cetoleic acid, astaxanthin, and organoarsenic compounds, such as arsenobetaine. Fish and shellfish are reasonably high in the trophic chain and tend to accumulate compounds present in their local pray or foods. This accumulation depends on the biotope, e.g., marine or fresh waters, geographical differences, and also on the fat content of the fish or shellfish. A high abundance of LCPUFAs, including docosahexaenoic acid (DHA), exists in marine plankton [168] while freshwater microalgae are generally forming other fats and only little eicosapentaenoic acid (EPA) [169]. Most fatty acids and xanthophylls are deposited in fats and accumulate more in fatty fish while TMAO is produced in some species to regulate osmotic pressure in deeper marine waters. Biomarkers of fish and shellfish may therefore depend on the local environments where these foods come from.

#### n-3 LCPUFAs

The n-3 LCPUFAs, EPA and DHA, as well as their intermediate product, docosapentaenoic acid (DPA), are high in marine fatty fish and in cod liver. Two to 20 times lower levels are found in most other aquatic meats, including shellfish, lean fish, some freshwater fish, and farmed fish from coastal waters or ponds, while terrestrial meat are 20–100 times lower, depending on their feed [170]. The n-3 LCPUFAs in blood are well-established fish-related biomarkers, known for their high specificity to fish and shellfish. In particular, fatty fish such as salmon, tuna, herring, and mackerel are affecting blood n-3 LCPUFAs [171]. Although eggs and meats originating from livestock fed with diets high in n-3 may also provide some quantities of EPA and DHA (e.g., through their higher consumption rates in many populations), marine and freshwater foods represent by far the main dietary source of n-3 LCPUFAs [171, 172]. LCPUFAs can additionally be synthesized in humans by converting *α*-linolenic acid (ALA) through a well-documented series of elongations and desaturations [173]. However, the conversion rate of ALA from plant sources is limited [174] and should therefore not interfere with the net content provided by marine sources.

EPA and DHA have often been used to assess compliance to fish and fish oil intake in intervention studies [175–179], and have been extensively associated with self-reported fish intake, measured either through 24-h dietary recalls or FFQs in a wide range of cohort and cross-sectional studies carried out in different populations [45, 180–183]. Furthermore, EPA and DHA have been widely used to validate proposed FFQs for assessment of fish intake [83, 184–187]. DHA, DPA, and EPA were also shown to increase in a 12w parallel RCT in adolescents comparing fish meals (herring, salmon, mackerel) with meat (chicken, turkey, beef, lamb) and cheese [188].

Blood is the most extensively explored matrix for measuring PUFAs; however, blood components differ in their metabolism and incorporation of LCPUFAs [189], thereby greatly influencing the duration of consumption reflected and the selection of the adequate blood fraction to match the intended outcome.

Plasma and serum have been suggested to reflect shorter-term intakes, whereas erythrocyte membranes and adipose tissue are more reflective of long-term (habitual) consumption of fish [190]. In serum, EPA, DHA, and total n-3 fatty acids increase within 4 h after intake [191], whereas their levels in cholesteryl esters (CE) and red blood cells (RBC) were only apparent after 3 days of supplementation. Incorporation of EPA and DHA was also shown in PC after 1–2 weeks, whereas levels in platelets were affected after 3–4 weeks and 1–2 months, respectively [192]. The incorporation of DHA was further reported to be slower than that of EPA in CE, RBC membranes, and the adipose tissue of healthy adults [193].

Plasma phospholipids act as transporters and are therefore a more transient compartment than RBC membranes [190, 194], with equilibrium reached after 1 and 6 months, respectively, at fixed dietary intakes [195]. The clearance of EPA and DHA from plasma phospholipids was also shown to occur more rapidly than from RBC [196], emphasizing on the ability of plasma phospholipids to adequately reflect only short-term changes in intake (weeks). Esterified LCPUFAs in whole blood were associated as strongly as LCPUFAs in erythrocytes in a fish oil intervention with 0.25–1 g/day of EPA and DHA over 4 weeks [197], and were shown to have similar kinetics within RBCs and in plasma, while EPA has faster kinetics than DHA [198]. The sum of EPA + DHA in plasma PCs was suggested as a suitable biomarker for recent EPA + DHA intake, while platelet and mononuclear cell EPA + DHA may reflect habitual intakes, showing reasonable dose-response relationship [192]. PC-EPA was also reported in an untargeted metabolomics investigation with fish oil [199] as well as in a cross-sectional association with aquatic meats [200], both measured in serum. Fish studies targeting PC-EPA and PC-EPA + PC-DHA may thus potentially shed light upon more accurate assessment of short-term fish intake.

Adipose tissue is less sensitive to changes in dietary n-3 PUFA than other lipid pools, thus fatty acids content in adipose tissue reflects only longer-term intake of fish oil [193, 201].

In studies where similar content of EPA and DHA was provided as either fish oil capsules or fatty fish, differentiation by plasma markers between these intakes is not possible [189, 202], nor is it possible to reflect different composition of the fish and fish oil [203].

Only one intervention study investigated the potential dose-response relationship between LCPUFAs after fish consumption [204]. EPA measured in plasma phospholipids showed dose-response at doses of 180 g and 270 g of salmon consumed twice a week for 4 weeks, but no significant difference from control was observed at 90 g of salmon. In contrast, DHA seemed to reach a plateau in this study even after 90 g salmon consumed, without a subsequent increase at higher doses [204]. When added together, EPA + DHA showed a dose-response starting with the lowest level of fish intake [204]. Other studies that investigated dose-response only used fish oils. In blood samples, EPA and DHA displayed dose-response in some studies with fish oil [193, 205–207, 209, 210]. Ulven and colleagues compared the efficacy of fish or krill (*Euphausia superba*, a crustacean) oils on n-3 LCPUFA contents in fasting plasma samples of healthy volunteers. Despite lower n-3 contents in krill oil, effects comparable to fish oil were observed for EPA, DPA, and DHA, suggesting that bioavailability of n-3 PUFA from krill oil is equal or even more efficient than that of fish oil [211]. The different efficiency may be attributed to different n-3 containing fats in krill oil, mainly phospholipids, compared with fish oil, mainly triglycerides. In a 14-day dose-response study, Hudson et al. have shown that incorporation of EPA, DPA, and DHA in PC shows dose-response in the range from 0.3–4.5 g EPA + DHA/day so that contents in PC is a sensitive and dynamic measure of recent LCPUFA intakes [212].

The use of DHA and EPA as BFIs for fish are likely to reflect mainly marine fish and should therefore be validated separately for different populations, as always recommended for BFIs [24]. In experimental studies, lean fish only affect n-3 LCPUFAs to a very limited extent [213] while these markers in observational studies have less power to discriminate lean and fatty fish intakes [180, 214–216]. Shellfish have a composition similar to lean fish but most observational studies on EPA and DHA did not differentiate between fish and other shellfish [184, 216, 217]. When shellfish intake was evaluated separately from non-fried fish intake in 900 participants from the Multi-Ethnic Study of Atherosclerosis (MESA), no association was observed between the concentrations of EPA and DHA in plasma phospholipids and shellfish intake [218]. When looking into intakes of more specific shellfish such as oysters, clams, crabs, squid, mussels, and shrimp in normolipidemic men, all shellfish increased n-3 PUFAs in fasting plasma and erythrocytes, except for clams that only elevated them in plasma, and shrimps that only showed an effect on RBC [219]. The plasma levels increased five times more than those in RBC, confirming that the turnover rate in different compartments is affected similarly by fish and shellfish. Tropical shellfish, unlike those from temperate waters, are a source of both n-3 and n-6 PUFAs, as shown by increased plasma levels of arachidonic acid reported together with the regular EPA, DPA, and DHA [220]; this underlines once again that BFIs should always be validated for each specific population taking their food sources into consideration.

#### Ratios of n-3/n-6 fatty acids

Fish intake negatively affects the plasma levels of certain omega-6 PUFAs. Linoleic acid (LA), gamma-linolenic acid (GLA), dihomogammalinolenic acid (DGLA), and arachidonic acid (AA), all n-6 PUFAs, have often been reported in studies on fish intake, but are of limited interest as fish intake biomarkers since they do not reflect fish intake per se but rather a change in their equilibrium with the n-3 FAs. Consequently, FA ratios such as n-3/n-6, n-6/n-3, the omega-3 index, and EPA/AA may be of potential relevance for health outcomes related to fish consumption, but are not robust as BFIs, as they are highly influenced by other food sources, although they may be relevant in some specific studies [204, 214, 221–224]. The same applies to the reported sum of n-3 and sum of n-6 PUFAs, measured in a handful of studies reported in Additional file 2: Table S3 [176, 191, 201, 204, 213, 214, 225–233], as they can easily be affected by the intake of other food groups than aquatic or terrestrial meat, such as vegetable oils and nuts.

In conclusion, n-3 LCPUFA in different blood compartments seem to be valid biomarkers of fatty fish intake and also of total fish, fish oil, and shellfish intake in populations consuming a mixed diet. The ratios, EPA/AA, (EPA + DHA)/AA, and (n-6)/LCPUFA may only be useful in controlled studies comparing fish and meat since they are too sensitive to other n-6 FA sources to be useful as general fish intake biomarkers.

#### Furan fatty acids

In addition to DHA and EPA, furan fatty acids have been recently suggested as biomarkers for fish/fish oil intake. CMPF was identified as a biomarker for fish consumption in fasting plasma, as its level was associated with dietary fish intake after a 12-week intervention trial with a Nordic diet [234]. In addition, another dietary intervention study has reported increased furan fatty acids after increased protein intake from fish and meat, including circulating levels of CMPF following consumption of a Mediterranean-type diet [235]. Plasma, serum, and urine CMPF has been observed also in free living populations across the Northern Hemisphere, where levels were associated with habitual dietary intake of fish and shellfish in several populations in Europe, China, and the USA [62, 236, 237]. Plasma as well as urine CMPF has also been observed to increase in hyperlipidemic patients after 600 mg/day of fish oil for 4 weeks and levels were still increased after 4 weeks’ wash-out [238]. Besides CMPF, four other compounds putatively identified as furan fatty acid metabolites were increased in blood after a diet high in fish [235, 239].

#### Trimethylamine-*N*-oxide

TMAO is a naturally occurring metabolite abundant in certain fish or generated from nutritional compounds including choline and carnitine by a microbiota mediated conversion of betaines to trimethylamine and further oxidation by hepatic flavin monooxygenases to TMAO [240, 241]. Several studies have shown increased urinary excretion of TMAO related to consumption of fish: a high level of TMAO in morning urine was associated with intake of fish containing PUFA and n-3 PUFA on the previous day [242]. Increased urinary TMAO was also observed within 4.5 h after a salmon-containing breakfast [47]. In an intervention with fish or meat dishes, urinary TMAO increased substantially after fish intake but not after standardized meals containing chicken, red meat, or processed meat [38]. In another crossover meal study with three fish dishes, three meat dishes and three lacto-ovo vegetarian dishes, TMAO increased in urine only after fish intake, peaking at 3 h [93]. TMAO in urine seems to show good specificity as a potential biomarker of fish and shellfish intake, because only fish and other aquatic meat products contributed to elevated urinary excretion of trimethylamine and TMAO, among 46 investigated food groups [240]. TMAO levels in plasma showed a dose-response with increased intakes [38], but also increased postprandially after intake of a fish meal, thus proving that the TMAO present in fish can be readily absorbed without the involvement of gut microbiota, while TMAO is elevated much later when metabolic precursors in meat are converted to TMAO by the gut-host co-metabolism [94]. TMAO is particularly high in sharks, skates, cods, and other fish diving in temperate or cold sea waters, serving as a muscle osmolyte, and it is also found in crustaceans from the same biotopes [243]. It may therefore be a better biomarker for intake of this group of fish and shellfish, e.g., from the North Atlantic Ocean. Plasma TMAO has recently evoked attention as potential risk factor for cardiovascular diseases [244]. However, this risk seems to be partially related to reduced kidney function [245] and also depends on the composition of the gut microbiota [94]. Therefore, the potential implication of TMAO from aquatic meat in disease development still needs scrutiny. In conclusion, TMAO in urine is a candidate short-term biomarker of intake for fish and shellfish from deeper water layers, at least in the North Atlantic Ocean, while studies of fish and shellfish from most other biotopes are still missing.

#### 1,2,3,4-Tetrahydro-*β*-carboline-3-carboxylic acid

In a randomized, single-blinded, crossover meal trial, metabolomics analysis was used to investigate plasma and urinary excretion following consumption of cod protein compared to other protein sources, such as whey, casein, or gluten. The tryptophan-derivative THCC was elevated in plasma and urine after cod protein intake [95]. Further studies to evaluate its specificity are needed, given the potential presence of THCC at considerable levels in cocoa products and in some fruit juices, jams, and fermented food products [246]. THCC is therefore not considered further as a fish intake biomarker.

#### Amino acids

Increased urinary excretion of taurine and 3-methylhistidine have been observed after fish and shellfish intake but have also been associated with animal protein consumption [247]. Though being less specific than TMAO, plasma levels of taurine, methylhistidines, and N6,N6,N6-trimethyl-lysine were also elevated following the consumption of cod protein [95]. Anserine, 1- and 3-methylhistidine were also reported in urine after a smoked salmon-containing breakfast [47]. As methylhistidines and taurine may be derived from several different sources of animal muscle, they are not specific to fish or shellfish and have therefore been discussed in the section on general markers of meat intake. However, in combined biomarker approaches, they may serve to distinguish between intake of fish (as part of “white meat”) from intake of mammalian red meats but this approach will need additional study.

#### Other lipids

Other lipids have been suggested as possible biomarkers of fish intake. In particular, a lipidomics investigation from O’Gorman et al. [248] demonstrated that a combination of specific lipid patterns had a reasonably good ability to discriminate between low and high dietary fish intake (AUC = 0.76) in 34 volunteers from the Metabolic Challenge Study (MECHE), with lysophosphatidylethanolamine (LPE) acyl C18:2 (LPE (18:2)) and phosphatidylethanolamine diacyl C38:4 (PE (C38:4)) identified as potential biomarkers in serum. Since C18:2 (LA) is an (n-6) fatty acids unrelated to fish intake, the association may be caused by confounding with, e.g., oils used for frying the fish. In another randomized controlled trial, a fatty fish-based diet produced an increase in the levels of lysoPC (20:5) and LPEs (20:5) and (20:6), which are very likely derived directly from the fatty fish [234]. Plasma phospholipid-LCPUFAs may serve as BFIs for shorter term or recent fatty fish intake in analogy with free LCPUFAs as already discussed.

In a study on plasma lipids in overweight men following meals with baked herring or baked beef, cetoleic acid (22:1) increased after herring intake, while 2-aminoadipic acid, leucine, *β*-alanine, and 4-hydroxyproline reflected beef intake [57]. Additional studies of cetoleic acid as potentially specific to intake of herring or of fish in general is needed for confirmation.

#### Astaxanthin

Astaxanthin is a natural lipophilic compound, which belongs to the xanthophylls and its bioavailability is enhanced by dietary lipids [249]. While it is found in a variety of aquatic organisms, including algae, krill, crayfish, salmon, salmon roe, shrimp, and crab, particularly high amounts are found in the flesh of salmon and in the carapace of crustaceans [250, 251]. No studies exist to evaluate the main food sources of plasma astaxanthin in different populations but in the Western World, salmon and shellfish are likely to be the major sources. Astaxanthin cannot be synthesized by animals or humans, but it is essential for the growth of Atlantic salmon alevins [252] and its presence therefore indicates accumulation from food. Astaxanthin isomers, preferentially absorbed and concentrated into VLDL in humans, contain cis-double bonds, whereas the all-trans astaxanthin is most accumulated in salmonoids [253, 254]. The biokinetics of astaxanthin have only been studied after supplementation. The maximal concentration of astaxanthin in serum is found at 12 h reaching C_max_ of 50–280 μg/L after intake of doses of 10–100 mg given as supplements and is then declining with a half-life of around 12–21 h [253–255]. Accumulation of astaxanthin has been indicated from doses of 1–3 mg over 4–12 weeks showing dose- and time-dependent increases up to final levels of 11 μg/L after 1 mg/day and 35 μg/L after 3 mg/day at 12 weeks [256]. Daily doses of 4 mg astaxanthin over 3 months were found to provide final plasma levels of around 30 μg/L in reasonable agreement with other data [257]. Feeding of hens with algae, specific yeasts, shellfish waste, or even synthetic astaxanthin has been shown to increase its presence in egg yolk, providing the basis for commercial “golden” eggs [258]. Other food sources in the human diet besides supplements are food colorants, e.g., E161. However, they are likely to be minor in populations ingesting aquatic meats. Background levels, presumably from aquatic meat intake, in middle-aged Japanese males were found to be 0.1–0.5 μg/L while background levels in Norwegian, German, or Dutch men and in Korean women were reported as non-detectable [254, 256, 259–261]. In groups of English and Russian subjects abstaining from aquatic meat for 10 days, levels were 0.25 μg/L [262]. In a study with 28 German men, recruited to consume 250 g wild or aqua-cultured salmon providing 5 μg astaxanthin/g salmon flesh in a randomized and double-blind trial over 4 weeks, plasma astaxanthin concentrations of around 25 μg/L were detected [261]. The bioavailability from salmon seems therefore comparable to or higher than what was observed with the supplements.

In conclusion, astaxanthin may not be specific to fish and shellfish but in populations with low intakes of other sources of astaxanthin, it can be considered a candidate biomarker of these foods. Since there is good concordance between plasma levels measured in different laboratories, astaxanthin is likely to have good reproducibility. However, inter-individual response to the same dose of astaxanthin was quite large in some of the studies [256, 261].

#### Organoarsenic compounds

It has been observed that many persistent organic pollutants (e.g., dioxin and organochlorine pesticides) and inorganic pollutants (e.g., mercury) are highly associated with fish consumption [263]. However, the presence of such compounds and elements in several other food matrices, as well as their differential distribution worldwide, create highly variable background levels, which do not allow the use of such compounds or their metabolites as reliable fish intake biomarkers [264]. Thus, for the search on environmental contaminants associated with the intake of aquatic meat, only arseno-compounds have been considered, given their high specificity to marine foods.

Due to the bioaccumulation of arseno-compounds along the aquatic food chain, fish and shellfish contain significant amounts of arsenic (As). In marine organisms, inorganic As present in ocean water is transformed into unique organic compounds by methylation or esterification with sugar (arsenosugars), lipid (arsenolipids), betaine (arsenobetaine, AsB), or choline (arsenocholine) [265]. AsB and methylated As (dimethylarsinate or DMAs, monomethylarsonate or MMAs) constitute the biggest proportion of As in fish and shellfish, while inorganic As(III) and As(V) are the least abundant metabolites [266]. Urinary profiles of As metabolites thus showed a positive dose-response relationship with recent fish or shellfish intake, despite differences based on species [267] and preparation method, since the water-soluble As metabolites are easily leaking from the meat when boiled [268].

Urinary excretion of DMAs 13–24 h after aquatic meat intake [266, 269] shows that As methylation is one of the As detoxification pathways in the human body [267, 268]. Urinary level of DMAs can thus also reflect As exposure from any other sources, such as seaweed [270], rice, mushroom [271], drinking water [269], or inhalation [272]. Consequently, DMAs and MMAs have low specificity as potential biomarkers of fish intake and are not considered further here.

Another major As metabolite, AsB, was rapidly excreted after fish and shellfish intake [95, 266, 268], showing limited biotransformation and accumulation. Formation of AsB from other As metabolites takes place in humans and AsB also influences urinary DMAs excretion [267, 269]. The extent of these processes may be limited since AsB was not associated with As content in drinking water [269] or inhaled air [272], and was only weakly associated with rice intake [269]. In addition, urinary AsB is less susceptible to inter-individual variation than other organoarsenic species, especially when expressed as concentration rather than as μg/g creatinine [266]. Adjustment for AsB or stratified analysis in some subjects with undetectable urinary AsB nullified the association between aquatic meat intake and urinary concentrations of total As or DMAs [270]. This finding supports the specificity of AsB as a biomarker of total fish and shellfish intake and AsB seems to be a promising candidate biomarker for these foods.

The most commonly investigated biospecimen for AsB quantification is urine [266, 269, 270]. We only found one intervention study by Stanstrup and colleagues [95] that examined the biomarkers of acute fish intake in both plasma and urine samples, however without comparing the performance of the two. We did not find any article discussing the measurement of plasma AsB as a potential indicator of habitual fish intake. Hence, current evidence support the usage of plasma AsB to reflect only recent intakes, and urinary AsB as an indicator of both recent (within 3 days) [269] and habitual fish and shellfish intake [270]. The kinetics of AsB varied across intervention studies, peaking in plasma within 4–14 h and returning to baseline after 8–50 h for intakes of cod [95, 267] and for urinary excretion after intake of crabs, wolfish, and lemon soles [272]. An observational study showed an association between urinary AsB and fish and shellfish intake within the last 72 h [266]. Association between urinary AsB and habitual aquatic meat consumption in the past year [270] was also observed at the group level but suggests no retention or tissue accumulation of AsB. Furthermore, as a natural, widespread water pollutant metabolite, AsB levels in fish or shellfish may vary across species and location [267]. Current evidence indicates that AsB may serve as a promising qualitative biomarker of fish and shellfish intake but its use for quantitative purposes needs further investigation. Studies so far were conducted in Caucasian populations in Europe [95, 266, 267, 272] and the US [270]; studies in other populations are therefore warranted. Other As-compounds such as arsenolipids and arsenosugars did not show enough potential as fish intake biomarkers because the biotransformation to larger organoarsenic metabolites does not always occur in the aquatic food chain. Moreover, in the human body, arsenosugars and arsenolipids are metabolized by the liver to thioarsenic [273] and As-containing fatty acids [274], respectively, rather than to DMAs or other smaller As-metabolites [267]. Inter-individual variability in arsenosugar metabolism has also been previously reported [273]. These As compounds are therefore unlikely to be good biomarker with specificity to fish or shellfish intake.

In conclusion, n-3 LCPUFA in blood are valid BFI for the intake of fatty fish and fish oil at the individual level and would therefore reflect compliance to these foods in experimental studies, with measurements in different blood compartments reflecting the average intakes over different time periods. Marine lean fish and shellfish also contribute to their levels, while the contribution from freshwater fish depends on their feed. Total n-3 LCPUFA in most blood compartments should therefore reflect overall fish and shellfish intakes in populations with mixed diets. CMPF and related furan fatty acid metabolites in blood or urine are not sufficiently investigated; they may reflect more recent intakes of fish, fish oil, and shellfish but might also reflect habitual intakes over the last 4 weeks or more, and may depend on the origins of these foods. TMAO in urine is a shorter-term marker of intake for certain marine fish and shellfish, especially those from cold-temperate, deeper sea levels. Additional biomarkers of mainly marine fish and shellfish may include arseno-betaines in urine and possibly plasma, but further validation is needed for these markers, as well as for CMPF and TMAO. A biomarker reflecting intake of lean freshwater fish has not been found in this literature review. No specific biomarkers arose from our separate search on shellfish.

### Validation of candidate biomarkers

The literature search outlined in the previous sections points to several putative biomarkers which may potentially be used to assess intake of animal protein, meat or fish and shellfish. However, several of these putative markers were not considered suitable as candidate BFIs for any of these foods. The reason for inclusion or exclusion of each compound from the list of putative markers is reported in Additional file 3: Table S4. The resulting list of candidate biomarkers have been further evaluated to assess their usefulness as BFIs through the eight validation criteria, previously proposed by the FoodBAll consortium [24] as reported in Table 1.

**Table 1:** Validation of candidate biomarkers for intake of meat and fish

Food item	Metabolites	Biofluid locations	Validation criteria								
			1. Plausibility	2. Dose-response	3a. Time-response (single meal)^a^	3b. Time-response (repeated intakes)^b^	4. Robustness	5. Reliability	6. Chemical and biological stability	7. Analytical performance	8. Reproducibility
Animal (protein)	δ15N + δ13C	Feces	Y	U	Y	Y	U	U	Y	Y	Y
		Urine	Y	U	Y	Y	U	U	Y	Y	Y
		Hair	Y	Y	N	Y	Y	Y	Y	Y	Y
		Blood	Y	Y	N	U	Y	Y	Y	Y	Y
All meat	Anserine	Urine	Y	U	U	U	Y	U	U	Y	U
	Creatine	Urine	Y	U	U	U	Y	U	U	Y	U
		Blood	Y	Y	U	U	Y	U	U	Y	U
	Hyp	Blood	Y	Y	U	U	Y	U	U	U	U
	3-MH	Urine	Y	Y	Y	U	Y	U	U	Y	U
		Plasma	Y	Y	U	U	U	U	Y	U	U
Terrestrial^a^ meat	Carnosine	Urine	Y	Y	Y	N	Y	U	U	Y	U
	3-MH + 1-MH + Carnosine	Urine	Y	U	Y	U	U	U	U	Y	U
Poultry	3-MH	Urine	Y	Y	Y	U	U	U	Y	Y	U
		Plasma	Y	Y	U	U	U	U	Y	Y	U
	Anserine	Urine	Y	U	U	U	U	U	U	Y	U
		Plasma	Y	Y	U	U	U	U	Y	Y	U
	Guanidinoacetate	Urine	Y	Y	Y	Y	Y	Y	U	Y	U
Heated meat	PhIP (free)	Hair	Y	Y	N	Y	U	Y	U	Y	Y
	MeIQx^b^	Urine	Y	Y	Y	Y	Y	Y	U	Y	Y
	PhIP^#^	Urine	Y	Y	Y	Y	N	N	U	Y	Y
	4′-OH-PhIP	Urine	Y	U	Y	U	U	U	U	Y	U
Fish and shellfish	δ15N + δ13C	Blood	Y	Y	N	Y	N	Y	Y	Y	Y
Fish (fatty)	EPA	Blood	Y	Y	U	Y	Y	Y	Y	Y	Y
	DHA^c^	Blood	Y	Y	Y	Y	Y	Y	Y	Y	Y
	EPA + DHA^c^	Serum, esterified	Y	Y	U	Y	Y	Y	Y	Y	Y
	EP + DHA^c^	RBC^d^	Y	Y	N	Y	Y	Y	Y	Y	U
	EPA/AA	Plasma phospholipids	Y	Y	U	Y	N	Y	Y	Y	N
	n-6/n-3 LCPUFAs	Plasma	Y	Y	U	Y	N	U	Y	U	Y
	TMAO	Urine	Y	Y	Y	U	Y	Y	Y	Y	Y
	CMPF	Urine	Y	Y	U	Y	Y	Y	Y	Y	Y
		Plasma	Y	Y	U	Y	Y	Y	Y	Y	Y
	Astaxanthin	Plasma	Y	Y	Y	Y	U	U	U	Y	Y
	AsB	Urine	Y	U	Y	U	Y	Y	Y	U	Y
		Plasma	Y	U	Y	U	U	U	U	U	U

*Y* yes, the criterion is fulfilled for at least some use of the biomarker; *N* no, the criterion has been investigated and not fulfilled; *U* unknown, the criterion has not been investigated or data is not available

^a^Mammals and poultry

^b^After enzymatic hydrolysis

^c^DHA and EPA work both singly and together

^d^Erythrocytes

All the candidate markers in Table 1 are plausible (question 1) in the sense that their origin or presence in the food is well known or explained and also reasonably unique for the food group. Regarding the ability to quantitatively estimate the intake of a certain food, most of the candidate BFIs seem valid at the population level; even though a good dose-response relationship has been reported for several of the BFIs at the individual or group level (question 2), we still have little evidence for exact quantitation of individual food intake in samples from observational studies. Variation may be high due to the high variability in the concentration of precursors in different foods, to differences in absorption and metabolism, and to matrix effects caused by other dietary factors. The use of BFIs for quantitative assessment of food intakes should therefore be carefully considered for each specific study population, sample type, and for each specific intake assessment. For the remaining questions, the available evidence depends on the marker and its application as outlined below.

None of the meat exposures can be assessed quantitatively and without limitations by any candidate BFI. In general, the development of meat biomarkers faces significant challenges, such as (i) the overlap between meat-related metabolites and products of human muscle catabolism, (ii) variations in raw meat caused by variation between and possibly also within species, and (iii) the influence of production systems and feeding regimes. In spite of this, some BFIs are promising candidates and can be used under some circumstances for each of the exposures; these are outlined and discussed below, in light of the eight validation criteria. Table 1 should be used as an indicator of lacking evidence for each candidate BFI that needs to be addressed in further research.

Animal protein intake may be determined by the changes in isotope ratios, i.e., *δ15N* and/or *δ13C* in urine, stool, hair or blood. Dose-response for both ratios has been observed for hair and whole blood. The repeated-dose time-response was established for hair samples for a single individual indicating that more than 5 months may be needed to change levels in hair close to the scalp. The time-course for blood has not been established, except that 1–4 weeks of dietary change seems insufficient. Time-response for urine and stool has not been established for single meals but for repeated intakes of diets based on animal or plant protein, and it was shown that 8 days of change was enough to alter the isotope ratios. The ratios in hair and blood are robust since they have been successfully applied in experimental as well as observational settings and they are reliable since they have repeatedly been validated against other intake measures, including dietary records and markers of aquatic meat intake. Robustness and reliability have not yet been established for the shorter-term markers in urine or stool. The ratios are chemically and biologically stable since stable isotopes cannot change with storage or handling and since they are known to work also in archeology. Moreover, their analytical performance is well established, their precision and accuracy is high, and inter-individual variability low, which has been observed in several laboratories, indicating also good reproducibility in terms of absolute values. The major weakness is the variation between populations due to food matrix effects and the lack of knowledge about the ability of the marker to pick up smaller differences in the average intakes of animal protein in individuals. Overall, the isotope ratios are excellent for comparing longer-term overall animal protein intakes within a population but need further refinement for measurements of shorter-term intakes or for estimating intakes accurately.

Total meat intake, including terrestrial and aquatic meats, may be assessed at the group level by 3-MH in blood or urine and potentially by anserine due to their presence in poultry and/or fish; the most promising markers of total meat intake at the individual level may be *creatine in blood or urine* or *Hyp in blood*. The latter (Hyp) may be a general marker of all meat since it reflects intake of cartilage, present also in offal. Dose-response has been shown only for the markers in blood. The measurements in blood seem robust and have been shown so far to associate with habitual intakes at the group level. Plasma Hyp has also been associated with meat intake in a few observational studies. Analytical performance seems adequate for creatine, while Hyp exists both free and as part of dipeptides and better validation is needed for each of these or for their combination. There are sparse data for validation of the individual variability, shorter- and longer-term time-response, reliability, and reproducibility of these markers. *Urinary creatine* has been proposed as a marker of habitual meat intake, with good robustness shown also in observational studies. Further validation studies are needed to evaluate reliability of these markers when different sources of meat are consumed.

Terrestrial meat (muscle) intake, including meat from mammals and birds, may be assessed by quantifying *carnosine in urine*. Carnosine seems to reflect only terrestrial meat intake, as it is absent in almost all aquatic meats. The marker has been reported to show dose-response and single meal time-response with no apparent accumulation upon repeated daily intakes; thus it is only reflecting recent meat intakes. The marker also seems robust in studies with a complex diet and it is strongly associated with other dietary assessment, i.e., FFQs at the group level, although there is still a paucity regarding the reliability at the individual level. Chemical and biological stability in urine and inter-laboratory reproducibility studies are also lacking while analytical performance seems adequate with LC-MS-based analytical platforms available for accurate measurements. Carnosine in combination with 1- and 3-MH, provided a better estimate of meat intake than each of the compounds alone [275]. Even though this model needs to be validated for reliability in observational studies, it underlines that measuring a combination of markers could optimize dietary assessment of meat intake.

Intake of white meat, i.e., poultry, but also some species of fish, may be assessed by the *urinary excretion of 3*-*MH or anserine*, *or by 3*-*MH in plasma*. Anserine is present in hen and turkey muscle and to a lesser extent in meat from several species of fish, including cod, salmon and tuna; excretion in humans may therefore mainly reflect poultry intake, while more studies are needed to assess the influence of fish on this marker. Human excretion of anserine has been shown to be strongly associated only with chicken intake and not with fish at the group level (high vs. non-consumers) in a small European sub-study [38]. Anserine excretion after dietary intake has acceptable analytical performance in several analytical systems, stability after longer-term storage, and robustness; but experimental evidence for dose-response and time-response is still missing, including for experimental studies with fish intake. On the other hand, the validation of *3*-*MH in urine* shows reproducible dose-, and time-response in experimental studies, although it is strongly associated with the intake of both bird and fish meat in observational studies. Increased excretion is also observed after red meat intake, albeit less strongly [276]; even so, experimental studies showing time- and dose-response of 3-MH excretion have been observed after intake of beef [43]. Inter-individual variation seems limited and background excretion rates with lacto-ovo-vegetarian diets are low. Stability is excellent and analytical performance is good in systems able to discriminate 1- and 3-MH [277, 278], but more data are needed regarding relative excretion levels after different sources of meat. Postprandial *plasma 3*-*MH* has been shown to respond stronger to chicken than to fish (haddock) with clear dose-response but with only marginal increases after high intakes of beef compared with other sources [38]. Plasma or serum 3-MH has not been extensively used as a marker in observational studies and most aspects of validation are therefore still missing, needing further research. From the scoring pattern, 3-MH in urine can be considered a valid compliance marker of poultry intake but may reflect general meat intake more broadly in samples from observational studies. Further validation studies are needed to study the relationship of 3-MH excretion with different kinds of aquatic meats.

Intake of cooked chicken may be quantified reliably by *urinary levels of guanidinoacetate*, which shows good dose-response, robustness, and analytical performance in a recent study from UK and Ireland [46] using NMR; guanidinoacetate in urine has also been observed in Denmark after a diet with mixed foods, including chicken [113]; however, further validation is needed in relation to other poultry meats and to potential geographical variation in the contents of this compound in chicken meat.

Average intake of heated muscle food may be assessed by the level of *PhIP in hair* while *urinary excretion of PhIP*, *4*-*OH*-*PhIP or MeIQx* after hydrolysis of conjugates may be markers of recent intakes. All markers can be quantified reliably, while sample pretreatment (hydrolysis) strongly affects the measured concentrations. Dose-response was shown experimentally for PhIP in urine during a 4-day intervention showing little tendency for metabolite accumulation. However, urinary markers show large inter-individual variation. Only measurements of MeIQx in urine showed some agreement with FFQ-based estimations of cooked meat intakes in an observational study and MeIQx therefore seems as the more robust and reliable of the two. 4′-OH-PhIP may also be useful as a urinary marker of fried meat but lacks most aspects of validation, thus needing further research. *Hair levels of PhIP* show promise for estimating average intakes of fried and grilled meat over several months confirming levels in experimental diets with evidence of dose-response; however, robustness and reliability in observational studies have not yet been documented.

Habitual intake of aquatic meats, including fish and shellfish, or of fish oil, may be assessed by *n*-*3 LCPUFA in whole blood*, *RBC*, *plasma or plasma phospholipids*. Among the metabolites associated with repeated or habitual fish intake, EPA, DHA, and total n-3 LCPUFA have shown reasonable ability to discriminate between terrestrial meat and fish intake or to estimate consumption of fish, shellfish, and fish oil supplements. EPA and DHA levels in various fractions in blood have mainly been investigated after repetitive intakes, showing clear dose-response with reported intakes; DHA was shown also to increase in plasma after a single meal in studies with cod or fish oil [95, 189], peaking at 2–4 h. In experimental dietary studies, the time to reach steady state of LCPUFAs after fish, shellfish, or fish oil was 2–6 weeks for whole blood, plasma, and plasma lipids; 8 weeks for serum; and 4 weeks to 6 months for RBC. Total plasma free or esterified EPA and DHA were proved to be sufficiently robust and all analytical criteria are fulfilled.

Recent intake of aquatic meats, including fish and shellfish, may be assessed by several markers, including *CMPF*, *AsB*, or *TMAO in urine*, and *CMPF or LCPUFA in plasma*. CMPF and TMAO respond postprandially to meals with fish, showing dose-response and time-response. Complete excretion of TMAO within 24 h is observed in subjects with normal renal function. CMPF has a longer half-life in blood and is not completely excreted in 24 h; in some groups of patients, the half-life may reach 4 weeks but better kinetic data are needed from experiments in healthy subjects. CMPF in blood seems to be observed in most cross-sectional studies of fish and shellfish intake while TMAO is not always associated with these intakes when cod or crustaceans are not an important part of the diets. CMPF seems therefore more reliable as a general fish marker in areas where furan fatty acids are present in the food chain but studies from additional areas of the world are needed to explore whether this is common everywhere. Both CMPF and TMAO are stable during storage, easily quantified but excretion is affected by kidney function, potentially making them reliable only in healthy subjects. Inter-laboratory comparison studies are not yet published but reported levels are comparable between laboratories, indicating fair reproducibility. *AsB* has known short-term kinetics in plasma and urine but repeated-dose experimental studies are still lacking. The compound is likely to vary with geographical region and cooking practices but has been shown to be reliable in some regions, reflecting habitual intakes at the group level measured by dietary records. The analytical performance is sufficiently good to show clear kinetics by untargeted measurements but formal analytical validation is lacking for urine as well as for plasma. *Plasma AsB* has been determined in a single experimental study. Much additional validation is therefore required for the use of AsB as a biomarker of aquatic meat intake.

## Conclusion

A range of biomarkers have potential to estimate meat and fish intake. The δ15N and/or δ13C ratios in hair are good determinants of long-term animal protein. Blood levels of creatine, hydroxyproline, and 3-MH are promising markers of total meat while carnosine works for terrestrial meats. However, more studies are needed to validate these markers, both alone and in combinations. The best validated markers are DHA and EPA in blood, serum, and blood lipids, clearly recognized as markers of habitual oily fish consumption. CMPF in blood and urine and TMAO in urine may also be useful markers to measure fish intake but depend on dietary habits. Overall, fish markers need validation in different populations. 3-MH seems to be a good biomarker of poultry intake showing dose-response, time-response, and good analytical performance but its robustness may be questioned because intake of some fish and possibly other meats are likely to interfere. Anserine is particularly good at characterizing poultry in both intervention and observational settings, but studies on time-response and dose-response are lacking. Guanidinoacetate excretion in urine is a promising new biomarker of chicken intake showing dose-response and robustness. No robust markers have been recognized so far to specifically assess red meat intake. BFIs able to discriminate between red and processed meats are also lacking. For fried and grilled meats, MeIQx in hydrolyzed urine and PhIP in hair are candidate biomarkers but need further work for their full validation. Additional studies on BFI discovery and validation are therefore needed before we can reliably assess or quantify intakes of all meat and most meat subgroups, except for aquatic n-3 LCPUFA.

## Supplementary information

**Additional file 1: TableS1.** Common keywords. **TableS2.** Specific keywords for each food group. **Figure S1.** Flow diagram of study selections describing each individual food group

**Additional file 2: Table S3.** Summary of the human studies reporting biomarkers positively related to intake of different kinds of meat, found in the systematic literature search. The studies are grouped and classified according to the category for which the biomarkers have been discussed in the main text.

**Additional file 3: Table S4.** Summary of the selected candidate biomarkers of meat and seafood intake and the discarded markers. For each marker, a brief explanation for inclusion or exclusion from the list of candidate FIBs is reported.

## Data Availability

Not applicable.

## Abbreviations

1-OHP: 1-Hydroxypyrene
1-OHPG: 1-Hydroxypyrene-O-glucuronide
4′-OH-PhIP: 2-Amino-1-methyl-6-(4′-hydroxyphenyl)imidazo[4,5-b]pyridine
4-HNE: 4-Hydroxy-2-nonenal
5-OH-PhIP: 2-Amino-1-methyl-6-(5-hydroxy)phenylimidazo[4,5-b]pyridine
8-iso-PGF2A: 8-Iso-prostaglandin-F2alpha
AA: Arachidonic acid
ALA: α-Linolenic acid
As: Arsenic
AsB: Arsenobetaine
ATNC: Apparent total N-nitroso compounds
BFIs: Biomarker for food intake
CE: Cholesteryl esters
CMPF: 3-Carboxy-4-methyl-5-propyl-2-furanpropionic acid
CVD: Cardiovascular disease
DHA: Docosahexaenoic acid
DHN-MA: 1,4-Dihydroxynonane mercapturic acid
DMA: Dimethylarsenate
DPA: Docosapentaenoic acid
EPA: Eicosapentaenoic acid
EPIC: European Prospective Investigation into Cancer and Nutrition
FFQ: Food frequency questionnaires
HAAs: Heterocyclic aromatic amines
Hyp: Hydroxyproline
LA: Linoleic acid
LCPUFAs: Long-chain polyunsaturated fatty acids
LPE: Lysophosphatidylethanolamine
MeIQx: 2-Amino-3,8-dimethylimidazol4,5-f]quinoxaline
MH: Methylhistidine
MMA: Monomethylarsenate
n-3: Omega-3
n-6: Omega-6
NAP: Naphthalene
NOC: N-nitroso compounds
PAHs: Polycyclic aromatic hydrocarbons
PC: Glycerophosphatidylcholine
PC: Phosphatidylcholine
PHE: Phenanthrene
PhIP: 2-Amino-1-methyl-6-phenylimidazo[4,5-b]pyridine
PhIP-M1: 7-Hydroxy-5-methyl-3-phenyl-6,7,8,9-tetrahydropyrido[3′,2′:4,5]imidazo[1,2-a]pyrimidin-5-ium chloride
Pro-Hyp: Prolyl-hydroxyproline
PUFA: Polyunsaturated fatty acids
RBC: Red blood cells
RCT: Randomized controlled trial
TBARS: Thiobarbituric acid reactive substances
THCC: 1,2,3,4-Tetrahydro-β-carboline-3-carboxylic acid
TMA: Trimethylamine
TMAO: Trimethylamine oxide
