# Supplementary material for: Biomarkers of meat and seafood intake: an extensive literature review
Source: Genes Nutr. 2019 Dec 30;14:35. doi: 10.1186/s12263-019-0656-4 (PMC6937850; doi:10.1186/s12263-019-0656-4)

Additional File 1. Table S1, Table S2 and Figure S1, describing the literature search criteria and the flow diagram of study selection.

TableS1. Common keywords

**Operator**	**Database**	**Field**	**Keywords**
	Pubmed	*All Fields*	biomarker* OR marker* OR metabolite* OR biokinetics OR biotransformation OR biomonit*#
	Web of Science	*Topic*	
	Scopus	*Article Title/ Abstract/ Keywords*	
**AND**	***Pubmed***	*All Fields*	trial OR experiment OR study OR intervention OR cohort[¥]
	Web of Science	*Topic*	
	Scopus	*Article Title/ Abstract/ Keywords*	
**AND**	***Pubmed***	*All Fields*	human* OR men OR women OR patient* OR volunteer* OR participant* OR individuals[§] OR subjects[¥]
	Web of Science	*Topic*	
	Scopus	*Article Title/ Abstract/ Keywords*	
**AND**	***Pubmed***	*All Fields*	urine OR plasma OR serum OR blood OR excretion OR hair[¥] OR toenail[¥]
	Web of Science	*Topic*	
	Scopus	*Article Title/ Abstract/ Keywords*	
**AND**	***Pubmed***	*All Fields*	intake OR meal OR diet OR ingestion OR consumption OR eating OR drink* OR administration
	Web of Science	*Topic*	
	Scopus	*Article Title/ Abstract/ Keywords*	

* wildcard; [¥] only for the category "meat in general" and "fish contaminants"; [§] only for the category "meat in general" ; # only for the categories "fried and processed meat".

TableS2. Specific keywords for each food group

**Fresh meat**			
**Overall meat intake**			
**Operator**	**Database**	**Field**	**Keywords**
**AND**	***Web of Science***	*Topic*	"Meat"
	Scopus	*Article Title/ Abstract/ Keywords*	
**Red meat**			
**Operator**	**Database**	**Field**	**Keywords**
**AND**	***Pubmed***	*All Fields*	"Red meat" OR "Dark meat" OR "Bush meat" OR "Wild Meat" OR Beef OR Pork OR "Horse meat" OR Mutton OR Goat OR Veal OR Lamb OR Venison OR "Wild boar" OR whale.
	Web of Science	*Topic*	
	Scopus	*Article Title/ Abstract/ Keywords*	

White meat			
**Operator**	**Database**	**Field**	**Keywords**
**AND**	***Pubmed***	*All Fields*	White meat OR Light meat OR Poultry OR Chicken OR “Turkey meat” OR Duck OR Goose OR “Game meat” OR Quail OR Pigeon OR Pheasant OR “Guinea fowl” OR Rabbit
	Web of Science	*Topic*	
	Scopus	*Article Title/ Abstract/ Keywords*	
**NOT**	***Pubmed***	*All Fields*	antioxidant* OR disease* OR health OR weight status OR oxidative stress OR toxin* OR carcinogen* OR allergen* OR carbon OR nitrogen OR inflammation OR environmental OR Antibody OR drug* OR pharmacokinetic* OR inhibitor* OR toxicity OR pesticide* OR cancer* OR treatment* OR pathogen* OR carcinoma OR ELISA
	Web of Science	*Topic*	
	Scopus	*Article Title/ Abstract/ Keywords*	
**Fish and Fish oil**			
**Operator**	**Database**	**Field**	**Keywords**
**AND**	***Pubmed***	*All Fields*	fish OR salmon OR herring OR trout OR perch OR pike OR pikeperch OR zander OR vendace OR cod OR haddock OR plaice OR shark OR hake OR mackerel OR anchovy OR sardine OR tuna OR swordfish OR seabream OR seabass OR eel OR chub OR barbel OR carp OR roach OR bluefish OR catfish OR mullet OR sole OR croaker OR flounder OR porgie OR whiting
	Web of Science	*Topic*	
	Scopus	*Article Title/ Abstract/ Keywords*	
**NOT**	***Pubmed***	*All Fields*	"polychlorinated biphenyls" OR organochlorine OR pesticides OR contaminant* OR pollutant* OR lead OR plumb* OR cadmium OR *brominated OR insecticide OR pesticide* OR herbicide* OR fungicide* OR toxin* OR neurotoxicant OR Perfluoroalkyl OR polycyclic aromatic hydrocarbons OR PAHs OR manganese OR selenium OR zinc OR copper OR Arsenic OR arseno* OR arsenate OR arsenite OR arsenate OR arsinic OR mercury OR Methylmercury OR methyl-mercury
	Web of Science	*Topic*	
	Scopus	*Article Title/ Abstract/ Keywords*	
**NOT**	***Pubmed***	*All Fields*	allergy OR allergic OR allergenic OR thyroid OR colitis
	Web of Science	*Topic*	
	Scopus	*Article Title/ Abstract/ Keywords*	
**NOT**	***Pubmed***	*All Fields*	rat OR rats OR mice OR mouse OR dog OR dogs OR rabbit OR rabbits OR monkey OR monkeys OR pig OR pigs OR piglet OR piglets OR buffalo OR buffaloes OR pigeon OR pigeons
	Web of Science	*Topic*	
	Scopus	*Article Title/ Abstract/ Keywords*	

**Fish contaminants**			
**Operator**	**Database**	**Field**	**Keywords**
**AND**	***Web of Science***	*Topic*	fish OR seafood OR shellfish OR mollusks OR crustaceans OR "marine food" OR sole OR mussels
	Scopus	*Article Title/ Abstract/ Keywords*	
**AND**	***Web of Science***	*Topic*	Arsenic OR arseno* OR arsenate OR arsenite OR arsinic
	Scopus	*Article Title/ Abstract/ Keywords*	
**NOT**	***Web of Science***	*Topic*	allergy OR allergic OR allergenic OR thyroid OR colitis
	Scopus	*Article Title/ Abstract/ Keywords*	
**NOT**	***Web of Science***	*Topic*	rat OR rats OR mice OR mouse OR dog OR dogs OR rabbit OR rabbits OR monkey OR monkeys OR pig OR pigs OR piglet OR piglets OR buffalo OR buffaloes OR pigeon OR pigeons
	Scopus	*Article Title/ Abstract/ Keywords*	
**Other Seafood**			
**Operator**	**Database**	**Field**	**Keywords**
**AND**	***Pubmed***	*All Fields*	seafood OR shellfish OR molluscs OR bivalves OR clams OR oysters OR scallops OR mussels OR cockles OR octopus OR squid OR cuttlefish OR calamari OR crustaceans OR Shrimps OR prawns OR crabs OR lobsters OR Crayfish OR urchin
	Web of Science	*Topic*	
	Scopus	*Article Title/ Abstract/ Keywords*	
**NOT**	***Pubmed***	*All Fields*	"polychlorinated biphenyls" OR "organochlorine pesticides" OR lead OR cadmium OR mercury OR Arsenic OR Methylmercury OR *brominated OR insecticide OR pesticide OR toxin* OR neurotoxicant
	Web of Science	*Topic*	
	Scopus	*Article Title/ Abstract/ Keywords*	
**NOT**	***Pubmed***	*All Fields*	allergy OR allergic OR allergenic OR "oxidative stress" OR inflammation
**Processed and highly heated meat**			
**Processed meat**			
**Operator**	**Database**	**Field**	**Keywords**
**AND**	***Pubmed***	*All Fields*	(salted OR cured OR smoked OR canned OR corned OR dried) AND (beef OR pork OR lamb OR chicken OR turkey OR horse OR mutton OR veal OR meat)) OR (ham OR bacon OR sausage OR patty OR salami OR jerky OR “hot dog” OR hotdog OR “meat loaf” OR “cold cut” OR “lunch meat” OR “luncheon

			meat”)
	Web of Science	*Topic*	(salted OR cured OR smoked OR canned OR corned OR dried) AND (beef OR pork OR lamb OR chicken OR turkey OR horse OR mutton OR veal OR meat)) OR (ham OR bacon OR sausage OR patt* OR salam* OR jerky OR “hot dog” OR hotdog OR “meat loaf” OR “cold cut” OR “lunch meat*” OR “luncheon meat*”
**Heated meat**			
**Operator**	**Database**	**Field**	**Keywords**
**AND**	***Pubmed***	*All Fields*	"done meat" OR heated OR roasted OR broiled OR grilled OR fried OR cooked OR "high temperature" OR baked OR Maillard OR Strecker OR "advanced glycation end products" OR "Polycyclic aromatic hydrocarbons" OR "heterocyclic amines" OR "advanced lipid peroxidation end products" OR “advanced glycation end products”
	Web of Science	*Topic*	"done meat" OR heated OR roasted OR broiled OR grilled OR fried OR cooked OR "high temperature" OR baked OR Maillard OR Stecker OR "advanced glycation end product*" OR "Polycyclic aromatic hydrocarbon*" OR "heterocyclic amine*" OR "Advanced lipid peroxidation end product*" OR “advanced glycation end products”
	Scopus	*Article Title/ Abstract/ Keywords*	
**Offal meat and other organs**			
**Operator**	**Database**	**Field**	**Keywords**
**AND**	***Pubmed***	*All Fields*	“organ meat” OR “variety meat” OR offal OR “foie gras” OR chitterling OR chitlin OR gizzard OR haggis OR tripe OR maws OR “chicken gizzard” OR “prairie oyster” OR kishka OR faggot OR “blood sausage” OR “black pudding” OR “black sausage” OR sweetbread OR “lamb fries” OR “calf fries” OR “rocky mountain oyster” OR pâté OR trotter OR brawn OR liver OR kidney OR spleen OR udder OR intestine OR heart OR lung OR bladder OR stomach OR cheek OR tongue OR thymus OR “parotid gland” OR “sublingual gland*” OR pancreas OR testicle OR brain OR head OR snout OR eyes OR ears OR lips

	Web of Science	*Topic*	“organ meat” OR “variety meat” OR offal OR “foie gras” OR chitterling* OR chitlin* OR gizzard OR haggis OR tripe OR maws OR “chicken gizzard” OR “prairie oyster” OR kishka OR faggot OR “blood sausage” OR “black pudding” OR “black sausage” OR sweetbread OR “lamb fries” OR “calf fries” OR “rocky mountain oyster” OR Pâté OR trotter* OR brawn OR liver OR kidney OR spleen OR udder OR intestine OR heart OR lung OR bladder OR stomach OR cheek OR tongue OR thymus OR “parotid gland*” OR “sublingual gland*” OR pancreas OR testicle* OR brain OR head OR snout OR eyes OR ears OR lips
**NOT**	***Pubmed***	*All Fields*	disease OR tumor OR tumour OR carcinoma OR treatment OR drug OR damage OR syndrome OR dose OR failure OR diagnosis OR symptom OR “blood pressure” OR “blood cell” OR “blood flow” OR dysfunction OR injury OR disorder OR hepatic OR renal OR cerebrospinal OR gastric OR myocard OR newborn OR child OR fetal OR pregnant OR cancer OR risk OR contaminant OR alcohol OR diabetes OR Colon OR plant OR exercise OR smoking OR coffee OR tea OR medicine OR stress OR bone OR gut OR fruit OR juice OR inflammation OR erythrocyte
	Web of Science	*Topic*	disease OR tumor OR tumour OR carcinoma OR treatment OR drug* OR damage OR syndrome OR dose OR failure OR diagnos* OR symptom* OR “blood pressure” OR “blood cell*” OR “blood flow” OR dysfunction OR injury OR disorder OR hepatic* OR renal OR cerebrospinal OR gastric OR myocard* OR newborn OR child OR fetal OR pregnan* OR cancer OR risk OR contaminant OR alcohol OR diabetes OR Colon* OR plant* OR exercise OR smoking OR coffee OR tea OR medicine OR stress OR bone OR gut OR fruit* OR juice OR inflammation OR erythrocyt*

Figure S1. Flow diagram of study selections describing each individual food group

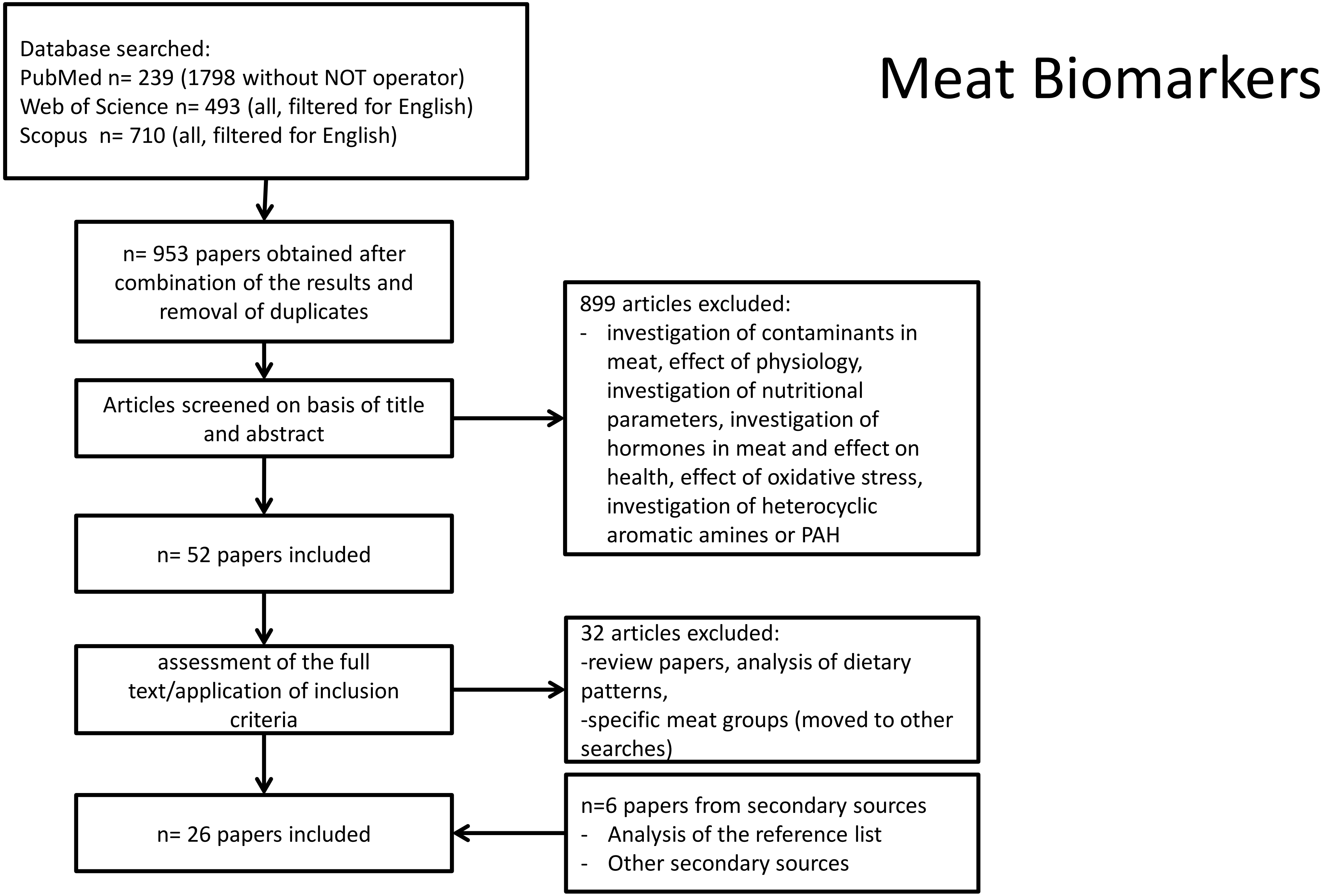

Mammalian (Red) Meat Biomarkers

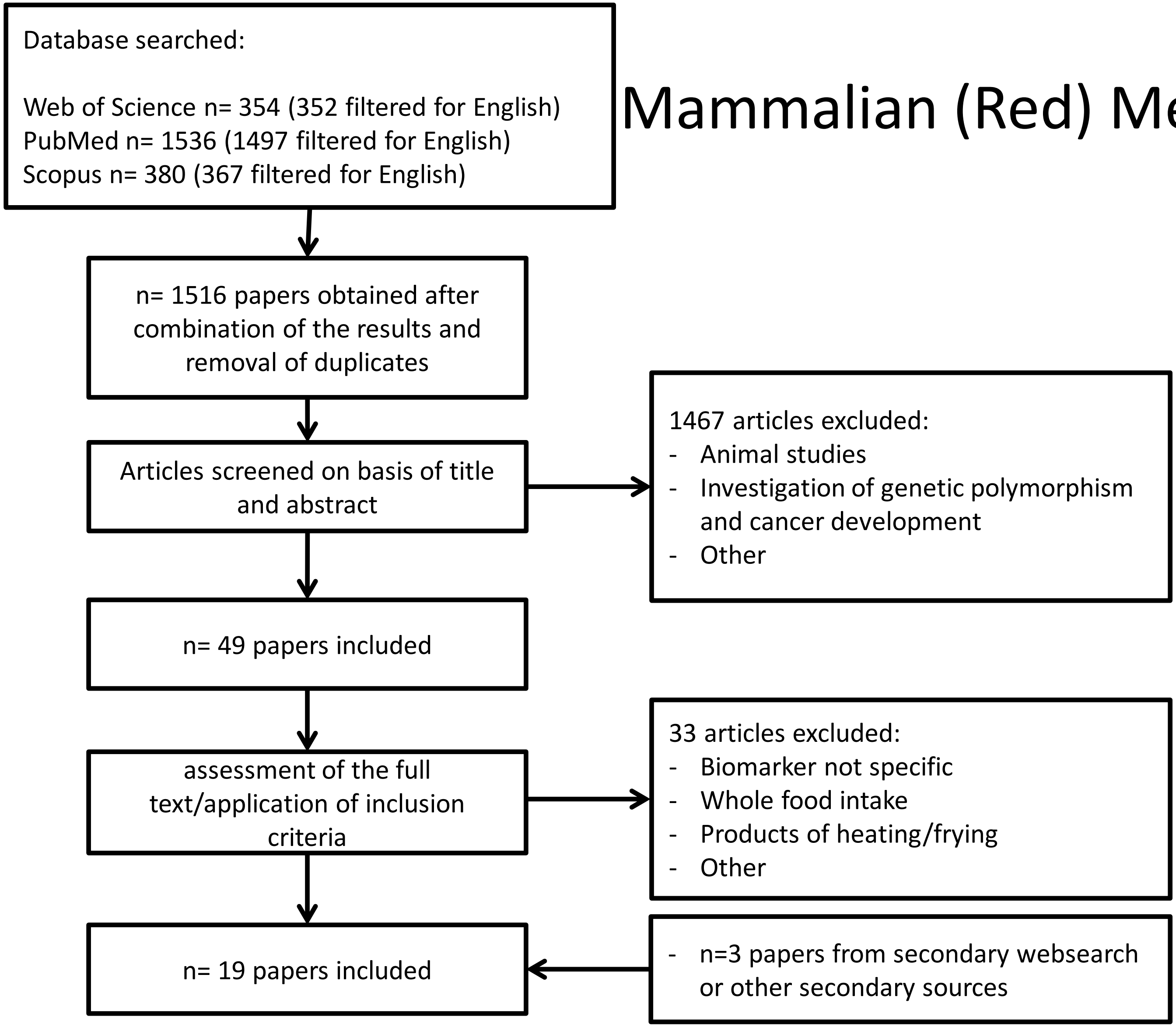

Offal Meat Biomarkers

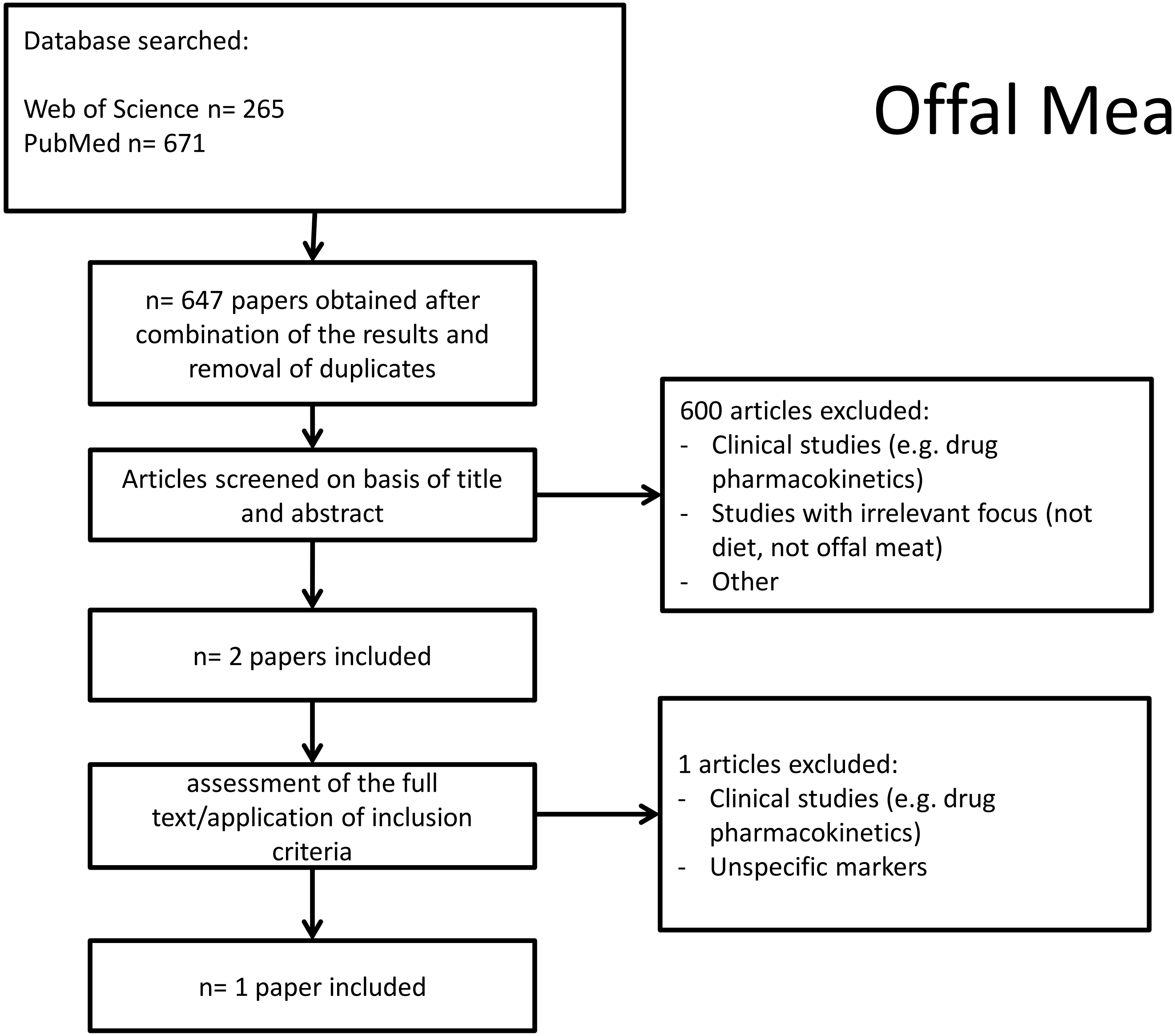

Poultry (white) Meat Biomarkers

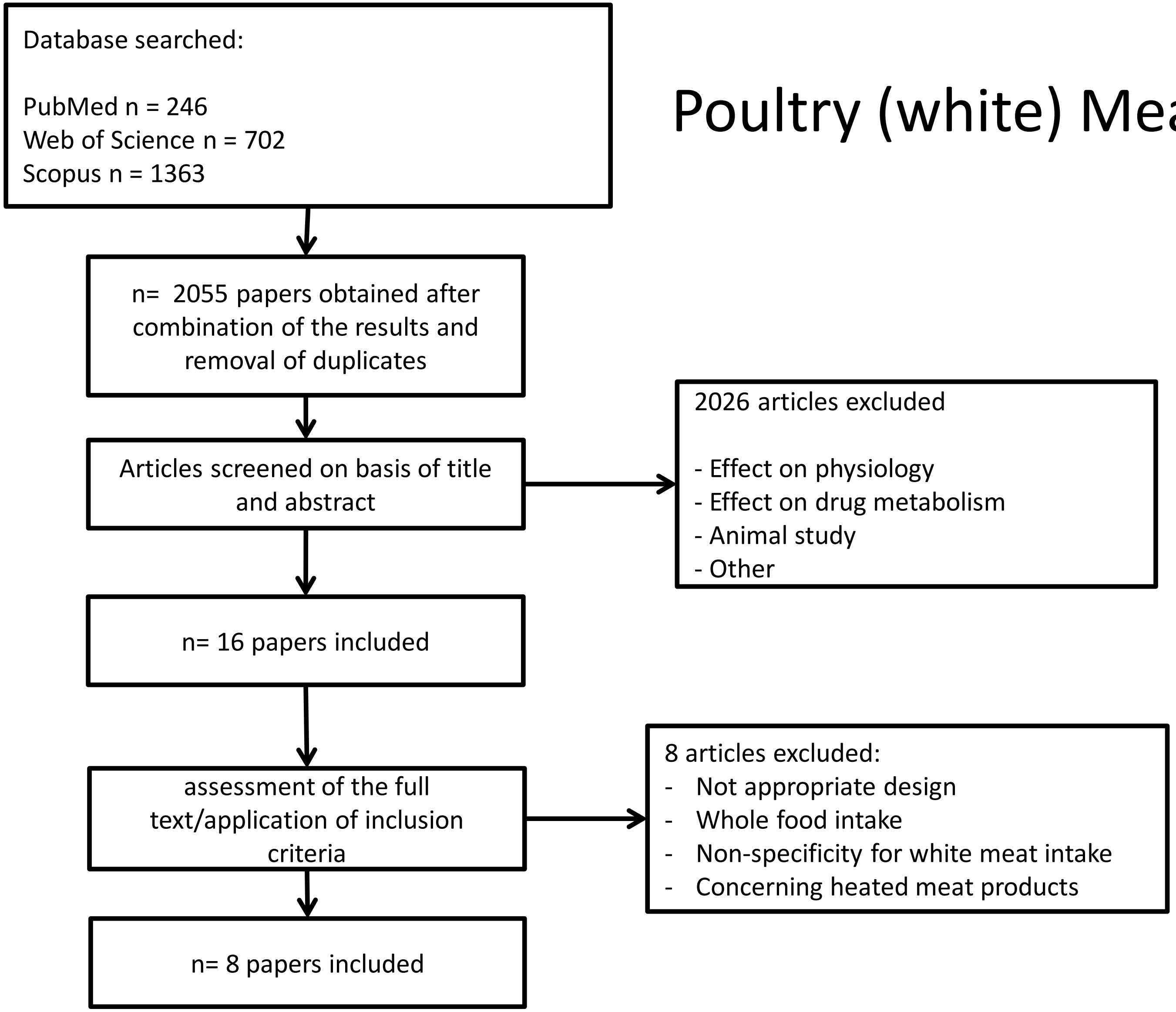

Heated Meat Biomarkers

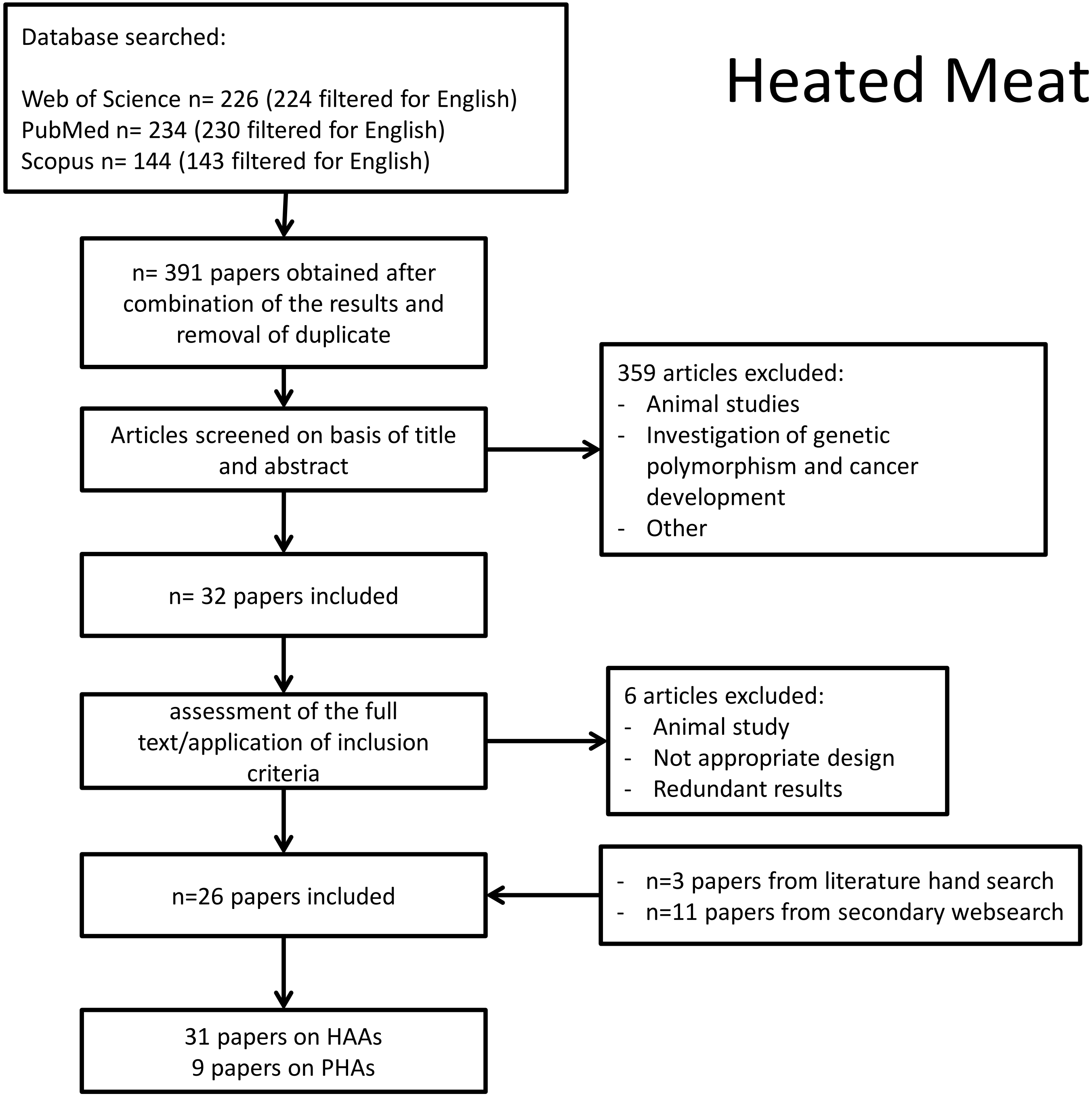

Processed Meat Biomarkers

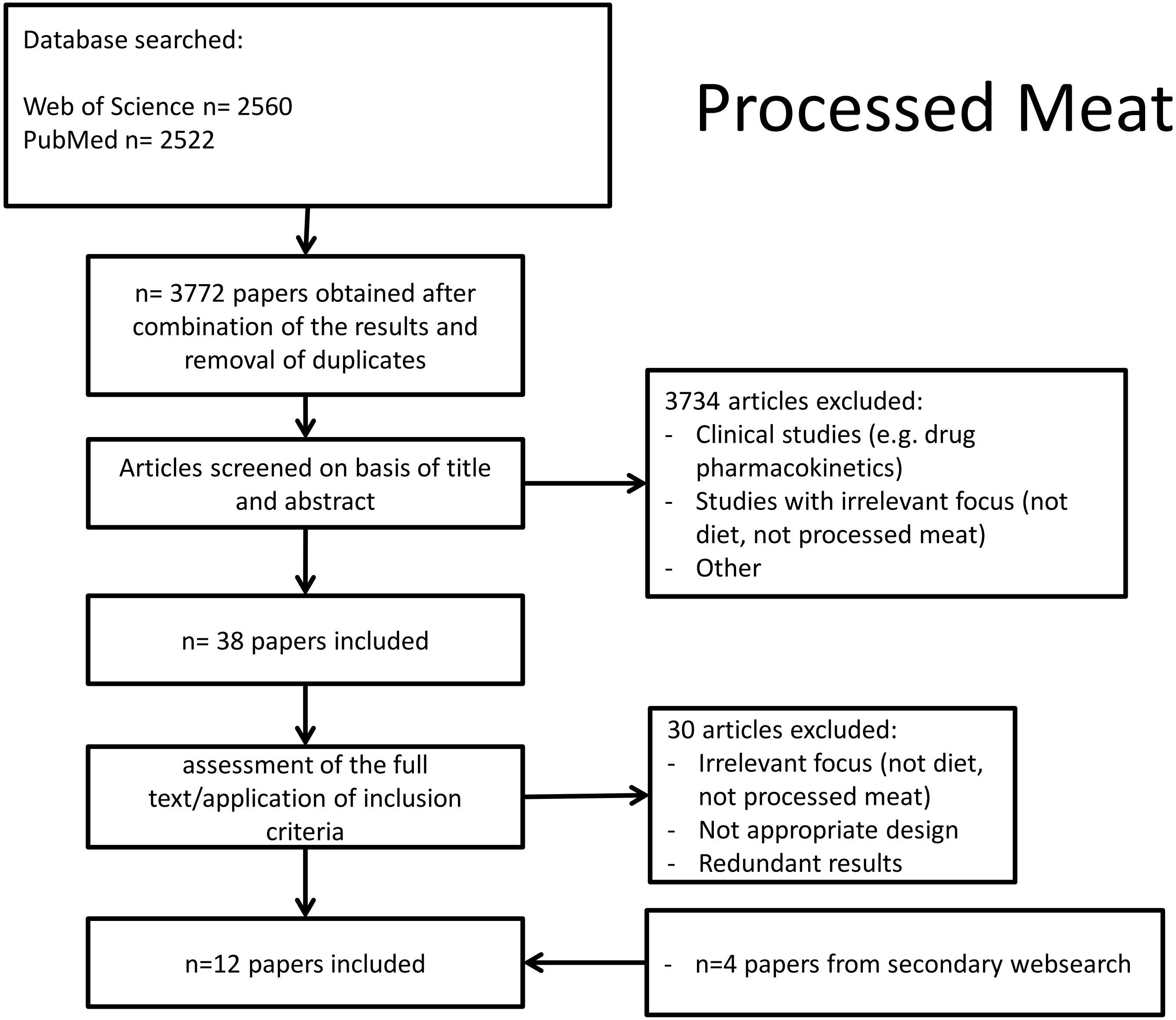

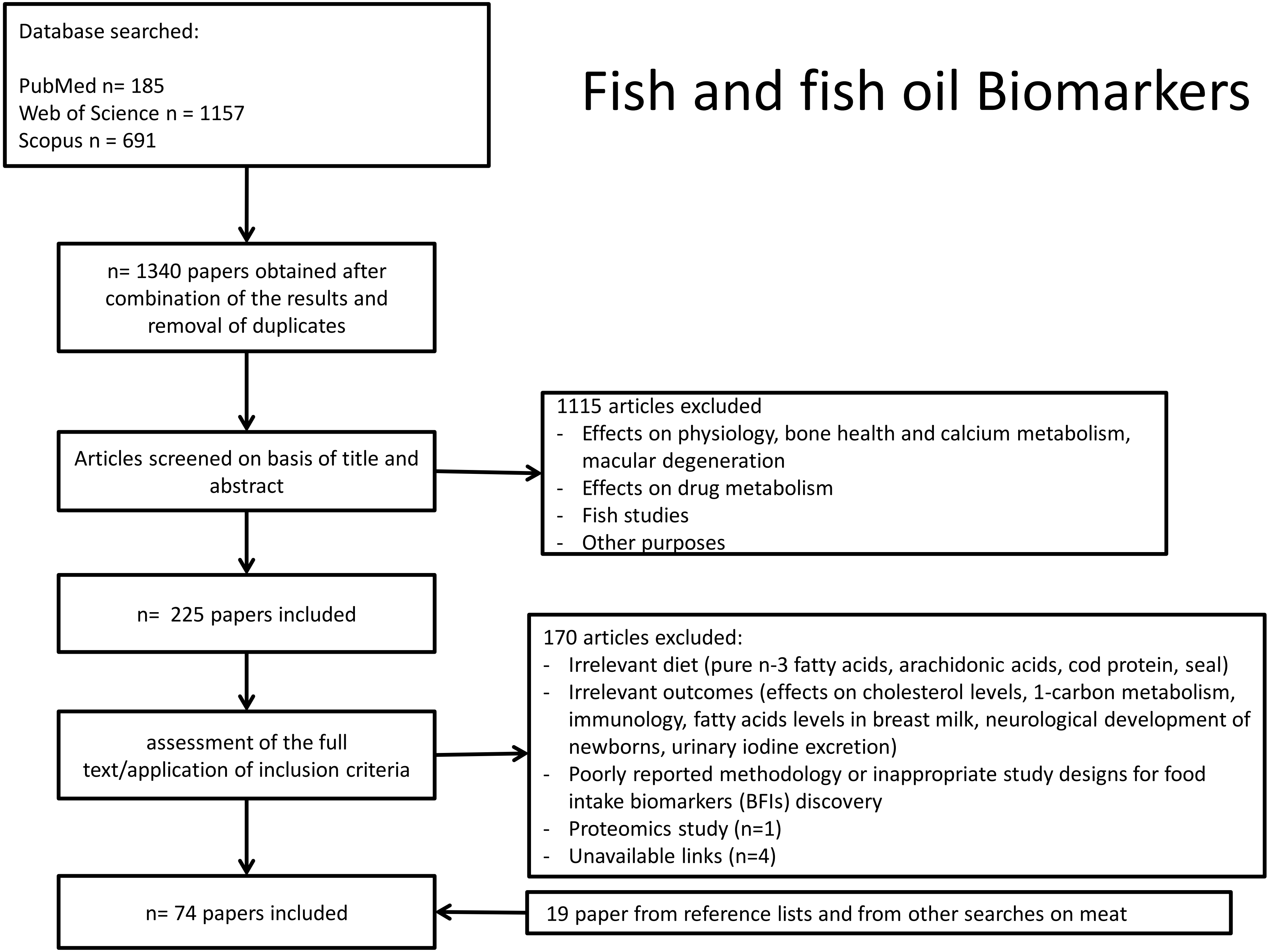
Fish and fish oil Biomarkers
Database searched:
PubMed n= 185
Web of Science n = 1157
Scopus n = 691
n= 1340 papers obtained after combination of the results and removal of duplicates
Articles screened on basis of title and abstract
1115 articles excluded
- Effects on physiology, bone health and calcium metabolism, macular degeneration
- Effects on drug metabolism
- Fish studies
- Other purposes
n= 225 papers included
assessment of the full text/application of inclusion criteria
170 articles excluded:
- Irrelevant diet (pure n-3 fatty acids, arachidonic acids, cod protein, seal)
- Irrelevant outcomes (effects on cholesterol levels, 1-carbon metabolism, immunology, fatty acids levels in breast milk, neurological development of newborns, urinary iodine excretion)
- Poorly reported methodology or inappropriate study designs for food intake biomarkers (BFIs) discovery
- Proteomics study (n=1)
- Unavailable links (n=4)
n= 74 papers included
19 paper from reference lists and from other searches on meat

Fish Contaminants Biomarkers

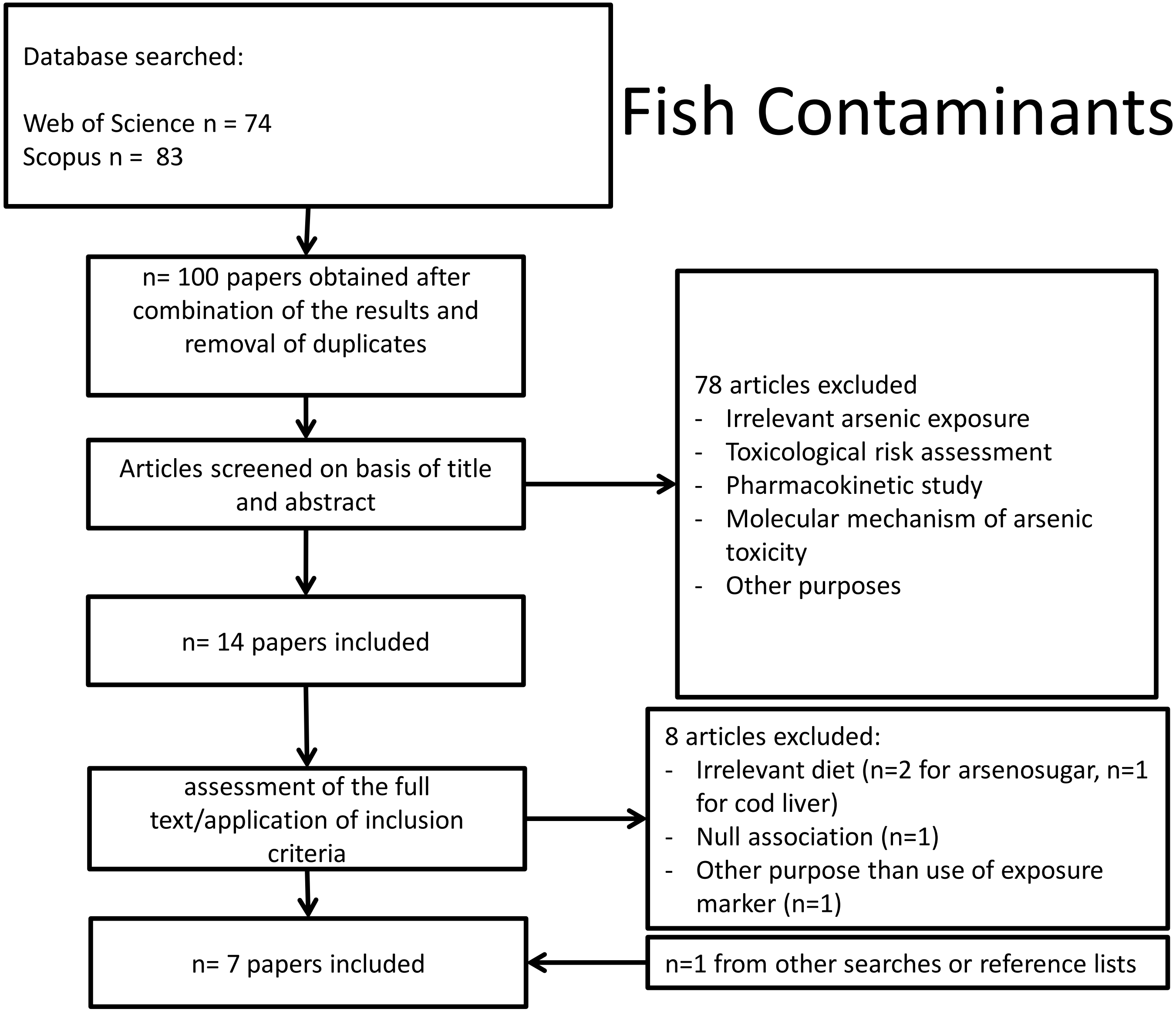

Other Seafood Biomarkers

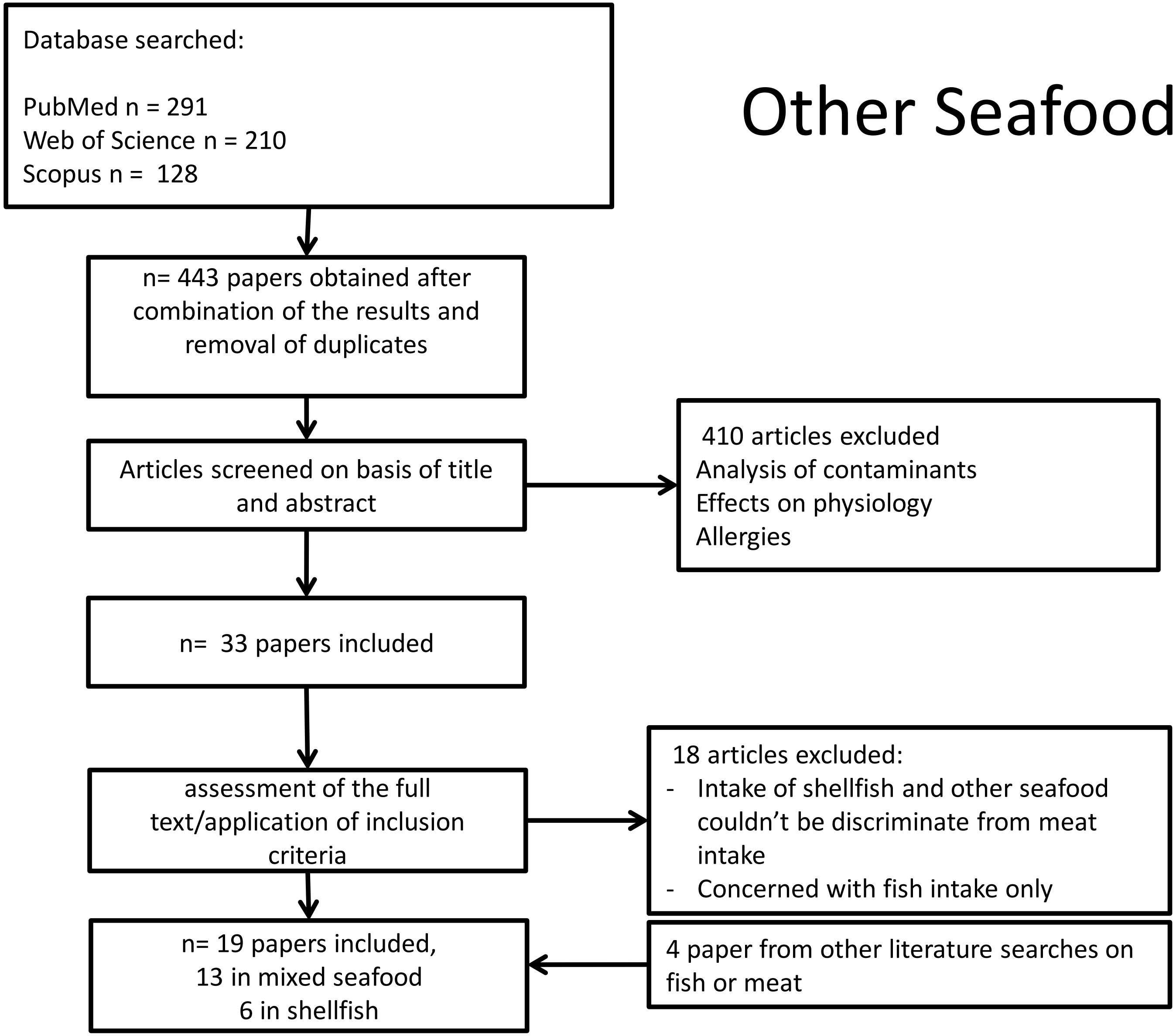

Supplement: Supplementary file 1 — Additional file 1: TableS1. Common keywords. TableS2. Specific keywords for each food group. Figure S1. Flow diagram of study selections describing each individual food group [file 12263_2019_656_MOESM1_ESM.pdf]
