# Supplementary material for: Biomarkers of meat and seafood intake: an extensive literature review
Source: Genes Nutr. 2019 Dec 30;14:35. doi: 10.1186/s12263-019-0656-4 (PMC6937850; doi:10.1186/s12263-019-0656-4)
Supplement: Supplementary file 2 — Additional file 2: Table S3. Summary of the human studies reporting biomarkers positively related to intake of different kinds of meat, found in the systematic literature search. The studies are grouped and classified according to the category for which the biomarkers have been discussed in the main text. [file 12263_2019_656_MOESM2_ESM.docx]

**Table S3.** Summary of the human studies reporting biomarkers positively related to intake of different kinds of meat, found in the systematic literature search. The studies are grouped and classified according to the category for which the biomarkers have been discussed in the main text.

| Dietary factor | Study  design | | Number  of  subjects | Analytical  platform | Sample type | Discriminating metabolites / Candidate biomarkers | Primary Reference(s) |
| --- | --- | --- | --- | --- | --- | --- | --- |
| General biomarkers of all meat intake | | | | | | | |
| Meat (vs. dairy and grain) | 3 x 1w crossover RCT with 18E% protein. | | 47 young men and women | LC-MS | 24-hour urine | Carnosine + 1-MH + 3-MH | [[1](#_ENREF_1)] |
| Terrestrial meats (beef, pork chicken, turkey) and fish oil vs. mixed lean seafood | 2 x 4w crossover RCT | | 20 healthy subjects (7 men), average age 51y | NMR | Morning spot urine | Guanidinoacetate  2,6-dimethylheptanoylcarnitine  Carnitine  N-methyl-2-pyridone-5-carboxamide  3-MH | [[2](#_ENREF_2)] |
| Mixed pork and chicken contrast with herring | 2 x 4w crossover intervention | | 15 obese men and women, 24-70y | GC-MS/MS profiling | Fasting plasma | Agmatine  x-MH | [[3](#_ENREF_3)] |
| Meat and seafood | 3 x 8d crossover RCT | | 14 young adults (6 men) | EA-IRMS | 24-hour urine  and stool | ^13^C/^12^C  ^15^N/^14^N | [[4](#_ENREF_4)] |
| Meat (high, low or none) | 3 x 15d crossover RCT with 7d washout | | 12 men (25-74y) | NMR | 24-hours urine | Creatine  Carnitine  Acetylcarnitine  TMAO  Taurine  1- and 3-Methylhistidine | [[5](#_ENREF_5)] |
| Meat (vs. dairy) | 7d parallel meal study | | 24 boys | NMR | Urine | Creatine  Histidine  Guanidinoacetate | [[6](#_ENREF_6)] |
| Meat vs. milk and bread | 7-10 day parallel study | | 4 young men one young woman | IEC | Urine | 1-Methylhistidine  3-Methylhistidine  Carnosine  Anserine | [[7](#_ENREF_7)] |
| Meat vs. Sustacal | 4d + 7d sequential study | | 14 young men | Amino acid analyzer | 24-hours urine | 1-Methylhistidine | [[8](#_ENREF_8)] |
| Meat and seafood | 5d sequential study | | 5  (2 men), 22-30y | Amino acid analyzer | Urine | 1-Methylhistidine | [[9](#_ENREF_9)] |
| Meat and other protein | 2 x 4d sequential | | 4 adult males | IEC | 24-hour urine | Taurine | [[10](#_ENREF_10)] |
| Meat (rabbit) | Single meal study | | 2 subjects (no details) | paper chromatography | Sequential urine samples | Anserine  Methylhistidines  β-alanine | [[11](#_ENREF_11)] |
| Meat (red, fried and processed meat, fish, shellfish and poultry) | Cross-sectional | | 3559 female twins, 18-84y | LC-MS/MS | Fasting blood (serum and plasma) | Creatine  Pyroglutamine  Trans-4-hydroxyproline | [[12](#_ENREF_12)] |
| Meat (red and processed) | Cross-sectional association with validation group | | 1491 (11% male) | LC-MS/MS | Fasting blood | Trans-4-hydroxyproline  Creatine  Pyroglutamine | [[13](#_ENREF_13)] |
| Meat and processed meat (as component of various diet scores) | Cross-sectional (Baseline of ATBC prospective study) | | 1336 men, 50-69y | LC-MS | Serum | trans-4-hydroxyproline  Ergothioneine  + one unknown | [[14](#_ENREF_14)] |
| Meat or fish (dietary abundance) | Cross-sectional | | 1254 men and women, 40-80y | EA-IRMS | Serum | ^15^N/^14^N  ^13^C/^12^C | [[15](#_ENREF_15)] |
| Meat (total) | Cross-sectional | | 1192 children, 6-11y | LC-MS and NMR | Urine  Serum | Creatine  PCs | [[16](#_ENREF_16)] |
| Meat (total) | Cross-sectional | | 909 (36% male) | LC-MS/MS | Overnight urine | 3-Methylhistidine | [[17](#_ENREF_17)] |
| Meat-based diet | Cross-sectional (baseline of prospective study, EPIC-Oxford) | | 379 men | LC-MS | Plasma | Acylcarnitines (C-0, C-4, and C-5)  Acylcarnitines (C-3, C-16)  Creatinine | [[18](#_ENREF_18)] |
| Meat or fish | Cross-sectional | | 297 (58% men) | NMR | Plasma | TMAO | [[19](#_ENREF_19)] |
| Meat (total intakes) | Cross-sectional | | 294 (101 men), 36-63y | LC-MS | Plasma | Anserine  Carnosine  3-MH | [[20](#_ENREF_20)] |
| Omnivorous diet | Cross-sectional study in vegetarians and omnivores | | 161 (83 men), 18-55y | NMR | Overnight urine | TMAO  Taurine  1- and 3-Methylhistidine | [[21](#_ENREF_21)] |
| Omnivorous diet | Cross-sectional study in vegetarians and omnivores | | 159  (80 men) | Colorimetry | Serum  Urine  Erythrocytes | Creatine  Creatinine (only in men)  Carnitine  Creatinine  Creatine | [[22](#_ENREF_22)] |
| Meat (high vs. low) | Cross-sectional | | 127 men and women, 20-68y | EA-IRMS | Hair | ^13^C/^12^C  ^15^N/^14^N | [[23](#_ENREF_23)] |
| All meat vs. vegetarian | Cross-sectional study in vegetarians and omnivores | | 126 middle-aged  (46 men) | IEC | Overnight urine | 3-Methylhistidine | [[24](#_ENREF_24)] |
| Meat (omnivores vs. vegetarians) | Cross-sectional | | 121 men and women, 17-68y | EA-IRMS | Hair | ^13^C/^12^C  ^15^N/^14^N | [[25](#_ENREF_25)] |
| Omnivorous diet | Cross-sectional study in vegetarians and omnivores | | 41  (22 men) | EA-IRMS | Hair | ^15^N/^14^N  ^13^C/^12^C  ^34^S/^32^S | [[26](#_ENREF_26)] |
| Omnivorous diet | Cross-sectional study in vegetarians and omnivores | | 30 (details not provided) | EA-IRMS | Hair keratin | ^15^N/^14^N  ^13^C/^12^C | [[27](#_ENREF_27)] |
| Omnivorous diet | Cross-sectional study in vegetarians and omnivores | | 161 (83 men), 18-55y | NMR | Overnight urine | TMAO  Taurine  1- and 3-Methylhistidine | [[21](#_ENREF_21)] |
| Biomarkers of mammalian (red and offal) meat intake | | | | | | | |
| Red meat discontinued intake | 4w Crossover RCT | | 113  (44 men), 21-65y | LC-MS/MS | Blood plasma and urine | TMA and TMAO | [[28](#_ENREF_28)] |
| Red meat (pork and beef) | 3 or 15d crossover in metabolic ward | | 18 males, 24-74y | TEA | Faeces | ATNCs | [[29](#_ENREF_29)] |
| Red meat (pork and beef) | 3 or 15d crossover in metabolic ward | | 17 males, 24-74y | IEC | 24-hour urine | 1-MH  3-MH | [[30](#_ENREF_30)] |
| Pork vs no meat | 2 x 4w RCT | | 14 women, 20-30y | EA-IRMS | Hair  Plasma  Urine | ^13^C/^12^C  ^15^N/^14^N | [[31](#_ENREF_31)] |
| Beef vs. fish | Crossover meal study | | 40 men, 21-50y | LC-MS/MS | fasting and postprandial plasma | TMAO  TMA  DMA | [[32](#_ENREF_32)] |
| Pork (ham) vs. non-meat | Crossover meal-study | | 24 (12 men), 18-65y | NMR | postprandial urine | Creatine | [[33](#_ENREF_33)] |
| Beef vs fish | Crossover meal-study | | 17 men, 41-67y | GC-MS | 0-7h postprandial blood plasma | β-alanine  4-hydroxyproline | [[34](#_ENREF_34)] |
| Beef vs chicken | Crossover meal-study | | 4 women (no details) | LC–MS/MS | Pre- and post-prandial urine | Anserine  Carnosine | [[35](#_ENREF_35)] |
| Red meat and  Offal meat | 15d sequential meals in metabolic ward | | 8 subjects, 24-74y | Competitive enzyme immunoassay | Urine | DHN-MA | [[36](#_ENREF_36)] |
| Red meat (fried beef and pork) vs- dairy and fish | 4-5d sequential meal studies with washout | | 10 adults, (5 men) | Amino acid analyzer | 24hr urine | 1-MH  3-MH | [[37](#_ENREF_37)] |
| Beef vs. no meat | Single meal study | | 18  (9 men), 18-25y | HPLC | Plasma (pre- and post-prandial) | Carnosine | [[38](#_ENREF_38)] |
| Beef | Single meal sequential study | | 7 (4 men), 25-60y | Amino acid analyzer | Urine (pre- and post-prandial) | 1-MH | [[39](#_ENREF_39)] |
| Pork | Single meal sequential study | | 1 male, 45y | HPLC | Urine (pre- and post-prandial) | Carnosine | [[40](#_ENREF_40)] |
| Beef | Single meal sequential study | | 1 male, 45y | HPLC | Urine (pre- and post-prandial) | Carnosine | [[40](#_ENREF_40)] |
| Red meat (beef steak and pork chops) | Cross-sectional study | | 3559 females | LC-MS/MS | Fasting blood (serum and plasma) | Trans-4-hydroxyproline  Pyroglutamine  Creatine | [[12](#_ENREF_12)] |
| Red meat | Cross-sectional study | | 1369 non-smoking women | LC-MS/MS | Urine | PE(P-18:0/20:4)  PE (P-18:0/18:1)  + one unknown | [[41](#_ENREF_41)] |
| Total red meat | Cross-sectional study | | 294 (101 men), 36-63y | LC-MS | Plasma | Carnosine  3-MH | [[20](#_ENREF_20)] |
| Red Meat | Cross-sectionally at baseline in a case-control colorectal cancer study (pearson correlations, FDR<0.1) | | 253 subjects, 125 cases and 128 controls (77 men), average age 18-74y | LC-MS or  GC-MS | 12 h overnight urine (no fasting) | Acetylcarnitine  Xylitol  3-dehydrocarnitine  Ethyl glucuronide  Carnitine  Cinnamoylglycine  Methyl-alpha-glucopyranoside  Sorbitol  + 10 unknowns | [[42](#_ENREF_42)] |
| Red meat | Cross-sectional at RCT baseline | | 125 (53 men), adults | NMR | Plasma and overnight urine | Acetylcarnitine | [[43](#_ENREF_43)] |
| Poultry |  | |  |  |  |  |  |
| Chicken | Parallel RCT meal study with 5 different meats | | 50 (5 x 10) subjects, 50% men, 51-64y | HILIC LC-MS | 24-hour urine | 3-MH  Anserine  + one unknown (pos; m/z=212.0914) | [[44](#_ENREF_44)] |
| Chicken | Crossover meal-study | | 4 women (no details) | LC–MS/MS | 6hr postprandial plasma  7hr postprandial urine | Anserine  Anserine  Carnosine | [[35](#_ENREF_35)] |
| Chicken vs. vegetarian | 24d sequential intervention and time-course study | | 35  (5 men), 20-30y | LC-MS | Plasma | 3-MH | [[45](#_ENREF_45)] |
| Chicken | 3w sequential dose-increase intervention study | | 10 (5 men), ~60y | NMR  LC-MS | Urine  Plasma | Guanidinoacetate  3-MH  1-MH | [[46](#_ENREF_46)] |
| Chicken | Single meal sequential study | | 1 male, 45y | HPLC | Urine (pre- and post-prandial) | Anserine  3-MH  Carnosine | [[40](#_ENREF_40)] |
| Poultry | Cross-sectional | | 3559 female twins, 18-84y | LC-MS/MS | Fasting blood (serum and plasma) | Creatine | [[12](#_ENREF_12)] |
| Poultry | Cross-sectional study | | 1369 non-smoking women, mean age 68y | LC-MS/MS | Urine | 3-MH  + one unknown | [[41](#_ENREF_41)] |
| Chicken | Cross-sectional study | | 565,  50% women, 18-90y | NMR | Fasting urine | Guanidinoacetate | [[46](#_ENREF_46)] |
| Poultry  Chicken  Turkey | Cross-sectional study | | 294  (101 men), 36-63y | LC-MS | Plasma | 3-MH  Carnosine  Anserine | [[20](#_ENREF_20)] |
| Poultry intakes | Cross-sectional study in vegetarians and omnivores | | 126 middle-aged subjects  (46 men) | IEC | Overnight urine | 3-MH | [[24](#_ENREF_24)] |
| Chicken vs. other meats | Cross-sectional study | | 46 (14 men), 40-70y | HILIC LC-MS | 24-hour urine | Seven unknowns (POS m/z= 178.0145,  255.0858, 259.1647, 124.0638,  240.1226, 282.1337, 185.0801) | [[44](#_ENREF_44)] |
| Biomarkers of highly heated meat intake | | | | | | | |
| Beef (fried), high/low doneness | 2 x 4w crossover intervention study | | 41 non-smokers (32 men),  18-63y | LC-MS/MS | Hair samples | PhIP (total) | [[47](#_ENREF_47)] |
| Beef (fried) vs no fried meat | 7w sequential diet intervention study w/o fried beef | | 44 ((36 men), >18y | LC-MS/MS | Hair samples | PhIP (total) | [[48](#_ENREF_48)] |
| Beef (fried) vs no fried meat | 7w sequential diet intervention study w/o fried beef | | 44 ((36 men), >18y | LC-MS/MS | 0-12h postprandially | PhIP  MeIQx  N(2)-hydroxy-PhIP-N2-glucuronide  N(2)-hydroxy-PhIP-N3-glucuronide  2-amino-3-methylimidazo-[4,5-f]quinoxaline-8-carboxylic acid (IQx-8-COOH)  2-amino-8-(hydroxymethyl)-3-methylimidazo[4,5-f]quinoxaline (8-CH2OH-IQx) | [[49](#_ENREF_49)] |
| Beef (fried) | 7w sequential diet intervention study and hair dying | | 14 non-smokers, >18y | LC-MS/MS | Hair samples | PhIP (total) | [[50](#_ENREF_50)] |
| Beef (charbroiled) | 19d sequential diet intervention study | | 10 non-smoking men, 25-45y | GC-MS | Postprandial urine at intervals from 0-72h | N(2)-hydroxy-PhIP-N2-glucuronide  N(2)-hydroxy-PhIP-N3-glucuronide  PhIP-4’-sulphate | [[51](#_ENREF_51)] |
| Beef (charbroiled) | 19d sequential diet intervention study | | 10 non-smoking men, 25-45y | GS-MS | First morning voided urine samples | PhIP (total) | [[52](#_ENREF_52)] |
| Beef (charbroiled) | 19d sequential diet intervention study | | 10 non-smoking men, 25-45y | IAC- SFS | First morning voided urine samples | 1-OHPG | [[53](#_ENREF_53)] |
| Beef (fried) vs. no fried food | Sequential meal study study | | 66 non-smokers  (33 men), 27-62y | HPLC-MS | Post-prandial 24-hour urine | total MeIQx and PhIP | [[54](#_ENREF_54)] |
| Beef (fried) vs. no fried food | Sequential meal study study | | 66 non-smokers  (33 men), 27-62y | GC-MS | Post-prandial 24-hour urine | N-OH-MeIQx-N2-glucuronide | [[55](#_ENREF_55)] |
| Beef (fried) vs. no fried food | Sequential meal study study | | 66 non-smokers  (33 men), 27-62y | GC-MS | Post-prandial 24-hour urine | N-OH-MeIQx-N2-glucuronide | [[56](#_ENREF_56)] |
| Beef (fried) vs. no fried food | Sequential meal study | | 66 non-smokers  (33 men), 27-62y | GC-MS | Post-prandial 24-hour urine | N-OH-PhIP-N2-glucuronide | [[57](#_ENREF_57)] |
| Beef (fried) vs. no fried food | Sequential meal study | | 66 non-smokers  (33 men), 27-62y | HPLC | Pre and post-prandial 24-hour urine | PhIP | [[58](#_ENREF_58)] |
| Beef (fried) w/o broccoli diet | Sequential meal study, 12 days washout. | | 20 non-smoking men | LC-MS/MS | Postprandial 0-48h urine | N(2)-hydroxy-PhIP-N2-glucuronide  N(2)-hydroxy-PhIP-N3-glucuronide  PhIP (total)  MeIQx (total) | [[59](#_ENREF_59)]  [[60](#_ENREF_60)] |
| Beef (fried) vs. no meat | Sequential meal study study | | 8 men, 40-57y | GC-MS | Pre and post-prandial 12-hour urine | PhIP (total)  MeIQx (total)  4'-OH-PhIP (total) | [[61](#_ENREF_61)] |
| Beef (fried) w/o broccoli | Sequential meal intervention study | | 6 women (no details) | LC-MS | Pre and post-prandial 12-hour urine | N-2-OH-PhIP-N-2-glucuronide,  PhIP-N-2-glucuronide,  4'-PhIP-glucuronide  N-2-OH-PhIP-N3-glucuronide | [[62](#_ENREF_62)] |
| Beef (fried) vs. no fried food | Sequential meal study | | non-smokers (details not provided) | LC–MS/MS | Post-prandial 48-hour urine | IQ  MeIQx  Trp-P-2  Trp-P-1  PhIP  AαC  Norharman  Harman | [[63](#_ENREF_63)] |
| Meat (cooked) | Single meal study | | 100 nonsmoking men, 18-34y | LC-MS | 12h pre- and post-meal | PhIP (free) | [[64](#_ENREF_64)] |
| Chicken (Fried) | Single meal study | | 12 non-smoking males | LC-MS/MS | Pre and post-prandial 24-hour urine | N2-OH-PhIP-N2-glucuronide  N2-PhIP glucuronide  N2-OH-PhIP-N3-glucuronide  4'-PhIP-sulfate | [[65](#_ENREF_65)] |
| Lamb kebab | Single meal study | | 12 non-smoking students (6 men) | HPLC | Urine | 13 different monohydroxy PAH metabolites (OHPAHs) | [[66](#_ENREF_66)] |
| Chicken (Fried) | Single meal study | | 11 men and women (no details) | LC-MS | Spot urine and post-prandial urine | PhIP  4′-OH-PhIP  5-OH-PhIP | [[67](#_ENREF_67)] |
| Chicken (Fried) | Single meal study | | 10 men and women, 25-45y | LC-MS/MS | Pre and post-prandial 24-hour urine | N2-OH-PhIP-N2-glucuronide  N2-PhIP- glucuronide  N2-OH-PhIP-N3-glucuronide  4’-PhIP-Sulfate | [[68](#_ENREF_68)] |
| Beef (fried), high/low intensity | Two single-meal studies | | 9+6 non-smokers | ^32^P-postlabelling | 24h urine samples | 1-OHP | [[69](#_ENREF_69)] |
| Chicken (barbequed) | Single meal study | | 9 non-smokers  (5 men),  23-61y | GC/MS | Urine | 1- and 2-hydroxy-NAP  2-, 3-, and 9-hydroxy-FLU  1-, 2-, 3-, and 4-hydroxy-PHE  1-OHP | [[70](#_ENREF_70)] |
| Pork (charcoal-barbecued) | Two single meal studies with different PAH dose | | 8 (3 men) + 5 (2 men) nonsmoking students | HPLC-FLD | Pre and post-prandial 12-hour urine | 1-OHP | [[71](#_ENREF_71)] |
| Pork (charcoal-barbecued) | Two single meal studies with different PAH dose | | 8 (3 men) + 5 (2 men) nonsmoking students | HPLC-FLD | Pre and post-prandial 12-hour urine | 3-OHBaP | [[72](#_ENREF_72)] |
| Chicken (Fried) | Single meal study | | 8 non-smokers  (4 men),  28-59y | LC-MS/MS | Post-prandial 12-hour urine | PhIP  4′-OH-PhIP  5-OH-PhIP  Norharman | [[73](#_ENREF_73)] |
| Chicken (Fried) | Single meal study | | 8 non-smoking healthy females | LC-MS/MS | Pre and post-prandial 24-hour urine | N2-OH-PhIP-N3-glucuronide,  PhIP-N2-glucuronide  4'-PhIP-sulfate (minor)  N2-OH-PhIP-N3-glucuronide (minor) | [[74](#_ENREF_74)] |
| Meat (fried beef or fish) | Single meal study | | 7 volunteers (no details) | GC-MS | Post-prandial 12-hour urine | MeIQx free and conjugated | [[75](#_ENREF_75)] |
| Chicken (Fried) | Single meal study | | 6 male non-smokers, 20-30y | LC-MS/MS | Urine and fecal samples up to 72 h after the meal | PhIP  PhIP-MI | [[76](#_ENREF_76)] |
| Meat (barbequed) | Single meal study | | 5 non-smokers (3 men), 25-53y | HPLC | daily 8-h urine | 1-OHP | [[77](#_ENREF_77)] |
| Meat (grilled, roasted, or broiled) | Cross-sectional study | | 304 women, 27-80y | IAC- SFS | 24-hour urine | 1-OHPG | [[78](#_ENREF_78)] |
| Fried meats vs. no fried food | Cross-sectional  study | | 129 male non-smokers, >35y | GC-MS | 24h urine | MeIQx (free and acid labile) | [[79](#_ENREF_79)] |
| Fried meats vs. no fried food | Cross-sectional  study | | 129 male non-smokers, >35y | LC-MS  GC-MS | 24h urine | PhIP (free and acid libile)  MeIQx (free and acid labile) | [[80](#_ENREF_80)] |
| Red (fried) and processed meat | Repeated cross-sectional study | | 111 women, 40-75y | IAC- SFS | Single spot urine sample in two seasons | 1-OHPG | [[81](#_ENREF_81)] |
| Meat (fried) vs. vegetarian | Cross-sectional study | | 35 smoking or non-smoking subjects (no other details) | GC-MS | blood albumin  erythrocyte globin | PhIP adducts | [[82](#_ENREF_82)] |
| Beef (fried) | Cross-sectional study | | 20  (7 men), 25-57y | LC-MS | Hair samples collected twice | PhIP (total)  MeIQx (total) | [[83](#_ENREF_83)] |
| Meat (grilled/stir-fried) | Cross-sectional FFQ validation study | | 20  (7 men), 25-57y | LC-MS | Hair samples collected twice | PhIP | [[84](#_ENREF_84)] |
| Meat (fried/grilled) | Cross-sectional study | | 14 non-smokers (6 men), 21-51y | GC/MS | Hair | PhIP | [[85](#_ENREF_85)] |
| Meat (Cooked) vs. no meat | Cross-sectional study | | 12 non-smokers, 6 meat-eaters, 6 vegetarians | LC-MS/MS | Hair samples | PhIP | [[86](#_ENREF_86)] |
| Processed meat | | | | | | | |
| Processed meat | 2 x 14 days crossover RCT study in metabolic suite | | 16 non-smokers (5 men), 20-85y | TEA | Fecal sample | ATNC | [[87](#_ENREF_87)] |
| Ham (Cooked) w/o Ca and vit E | 2 x 4d cross-over intervention study | | 17 men, 40-75y | TEA (for ANTC)  UV-VIS spectrometry (for TBARS and heme) | Fecal sample | ATCN  TBARS  heme | [[88](#_ENREF_88)] |
| Processed meat vs. other meats | Parallel RCT meal study with 5 different meats | | 50 (5 x 10) subjects, 50% men, 51-64y | HILIC LC-MS | 24-hour urine | Two unknowns (POS m/z=240.1226,  160.0849) | [[44](#_ENREF_44)] |
| Nitrite-preserved meats vs. vegetarian | Sequential dietary change intervention study | | 6 (4 male, 2 female), 37-55y; 22 vegans, 18-65y and 14 vegetarians, 26-36y | GC-TEA | 4-day 24-hour urine collections | N-nitrosoproline | [[89](#_ENREF_89)] |
| Smoked food | Single-meal study | | 13 smokers and non-smokers (no details) | HPLC-FLD | Urine collections over 24 hours | 1-OHP | [[90](#_ENREF_90)] |
| Sausage and bacon | Cross-sectional study | | 3559 female twins, 18-84y | LC-MS/MS | Fasting blood (serum and plasma) | Pyroglutamine  Creatine | [[12](#_ENREF_12)] |
| Processed meat | Cross-sectional study | | 1369 non-smoking women | LC-MS/MS | Urine | X-18922 | [[41](#_ENREF_41)] |
| Processed meat | Cross-sectional analysis of nested case-control study | | 502 CRC cases and controls (281 men), 55-74y | LC-MS/MS  GC-MS | Serum | Lathosterol | [[91](#_ENREF_91)] |
| Processed meat | Cross-sectional | | 294 (101 men), 36-63y | LC-MS | Plasma | Anserine | [[20](#_ENREF_20)] |
| Processed meat | Cross-sectionally at baseline in a case-control colorectal cancer study (pearson correlations, FDR<0.1) | | 253 subjects, 125 cases and 128 controls (77 men), average age 18-74y | LC-MS or  GC-MS | 12 h overnight urine (no fasting) | Acetylcarnitine  Carnitine  + 3 unknowns | [[42](#_ENREF_42)] |
| Processed meat | Cross-sectional study | | 239 males, 55-79y | ESI-MS/MS | Blood sample | PC(38:4) | [[92](#_ENREF_92)] |
| Processed meat (bacon, pork/ham and sausage/  luncheon meats) | Cross-sectional study | | 131 subjects (61 smokers), >35y | GC-MS | Overnight urine | MeIQx (total) | [[93](#_ENREF_93)] |
| Processed meat | Cross-secttional study | | 46 (14 men), 40-70y | HILIC LC-MS | 24-hour urine | Carnosine  2-Methylbutyrylcarnitine  Propionylcarnitine  1-Methylhistidine  3-Methylhistidine  + two unknowns (POS m/z=240.1226,  160.0849) | [[44](#_ENREF_44)] |
| Biomarkers of fish intake | | | | | | | |
| New Nordic Diet (fish component) | | 3 mo. Crossover RCT | 834 school children, (434 boys) 9-11y | GC | Fasting whole blood | EPA  DHA | [[94](#_ENREF_94)] |
| Fish vs. meat | | 12w Parallel RCT | 415 adolescents (195 boys), 14-15y | GC | serum | EPA  DHA  DPA  omega3 index (n-3 PUFA/total FA) | [[95](#_ENREF_95)] |
| New Nordic Diet (fish component >43g/10MJ) vs. average Danish diet | | 6 mo. Parallel RCT | 181 centrally obese (52 men), 18-65y | LC-MS | Urine | TMAO | [[96](#_ENREF_96)] |
| Nordic diet with high fish vs. habitual | | 18-24w Parallel RCT | 166 middle-aged overweight subjects (63 men) | GC | Fasting blood samples | EPA  DHA | [[97](#_ENREF_97)] |
| Fatty fish vs. other meat  Salmon  Herring | | 8w Parallel RCT | 126 women, 35-70y | Capillary-GC | Fasting blood samples at baseline and after 8wks | DHA  EPA+DHA  n-3 FA  n-6/n-3 PUFA ratio  EPA  EPA | [[98](#_ENREF_98)] |
| Nordic diet (fish component) | | 12w Parallel RCT | 106 with metabolic syndrome (age and gender not provided) | HPLC-MS | Fasting blood plasma samples | CMPF | [[99](#_ENREF_99)] |
| High-protein Mediterranean-style diet vs. AHA control | | 6 mo Parallel RCT | 96 overweight subjects (52 men) average age 49y |  | plasma | CMPF  EPA | [[100](#_ENREF_100)] |
| Salmon Atlantic Farmed, 3 servings/wk vs control | | 6mo Parallel RCT | 95 male sexual offenders in custody, 21-60y | GLC | Fasting blood samples  (erythrocytes) | EPA+DHA | [[101](#_ENREF_101)] |
| Salmon (Atlantic) vs. lean fish | | 8-week Parallel RCT | 92 dyslipidemic men, 35-70y | GC | Fasting blood samples | EPA  DHA  DPA  n-6/n-3 PUFA ratio  n-3 PUFA | [[102](#_ENREF_102)] |
| Fatty fish vs. lean fish | | 12w Parallel RCT | 79 pre-diabetic subjects (40 men), 43-72y | GLC | Esterified FA in plasma | EPA  DPA  DHA | [[103](#_ENREF_103)] |
| Herring (5 meals/week) vs. chicken and pork | | 2 x 6w crossover RCT | 35 overweight men, 35-60y | GC | Whole blood | (EPA + DHA):AA  EPA  DHA | [[104](#_ENREF_104)] |
| Cod and haddock | | Parallel RCT meal study with five different meats | 46 (14 men), 40-70y | HILIC LC-MS | 24h-urine samples  Fasting plasma samples | 3-MH  TMAO  (no markers) | [[44](#_ENREF_44)] |
| Wild or farmed salmon | | 4w parallel double-blinded RCT | 28 healthy men, 20-49y | HPLC | Plasma on days 0, 3, 6, 10, 14 and 28 | Astaxanthin | [[105](#_ENREF_105)] |
| Mackerel | | 4w Parallel RCT | 28 men, 21-28y | Capillary GC | Plasma | n-3 FA  EPA  DHA | [[106](#_ENREF_106)] |
| Salmon | | 3 x 4w crossover RCT | 25 subjects (14m, 11f) | GC | Erythrocyte membranes | EPA  DHA | [[107](#_ENREF_107)] |
| Fatty fish (2x per week) | | 4w Crossover RCT | 25 (14 men), 23-65y | Capillary-GC | Fasting plasma PL | EPA  DHA | [[108](#_ENREF_108)] |
| Salmon (smoked) vs. non-meat meals | | 4 x Cross-over (latin squares) meal study | 24 healthy volunteers (sex and age not provided for all), >18y | FIE-MS and GS-MS | 0, 1.5, 3 and 4.5h urine samples | TMAO  Anserine  x-MH  + one unknown | [[109](#_ENREF_109)] |
| Fish (high vs. low) | | 24w parallel RCT | 22 (10 men), >40y | GC-MS | 12-h fasting blood samples | EPA  DHA | [[110](#_ENREF_110)] |
| Salmon (Atlantic farmed), 180, 360 or 540g/w | | 3 x 4w crossover RCT, 4-8w washout | 19 healthy men and women (numbers not provided), 40-65y | GC | Fasting plasma PL | EPA  DHA  n-3 PUFA  EPA+DHA  n-6/n-3 PUFA ratio | [[111](#_ENREF_111)] |
| Herring vs. pork and chicken | | 2 x 4w crossover RCT, 2w washout | 13 overweight or obese subjects, 24-70y | Capillary-GC | Fasting plasma samples | EPA/AA ratio  EPA  DHA  n-6/LCPUFA  n-6/n-3 | [[112](#_ENREF_112)] |
| Lean fish (cod) vs. beef | | Crossover meal study | 40 men, 21-50y | LC-MS/MS | fasting and postprandial plasma  Urine | TMAO  MA  DMA  TMAO | [[32](#_ENREF_32)] |
| Salmon (smoked) vs. non-fish meals | | Cross-over meal study | 24 healthy volunteers (gender not provided), >18y | FIE-MS, GS-MS | 1.5-, 3-,  and 4.5-h postprandial urine | x-MH  Anserine  TMAO  Unknown (m/z= 221.06445) | [[113](#_ENREF_113)] |
| Fish vs. animal or vegetable protein | | Crossover meal study (9 meals) | 17 (4 men), 20-30y | LC-MS | 24-hour urine | TMAO | [[114](#_ENREF_114)] |
| Lean fish (cod) | | Crossover RCT meal study | 11 obese pre-diabetics (3 men), 40-68y | GLC | Plasma | TMAO  N,N,N-trimethyllysine  1,2,3,4-Tetrahydro-β-carboline-3-carboxylic acid  Arsenobetaine (AsB)  Methylhistidines | [[115](#_ENREF_115)] |
| Salmon (Atlantic) vs. meat or vegetarian | | 3 x 3w sequential study with 3w washout | 29 healthy subjects (14 men), 22-52y | Capillary-GC | Fasting Serum  Blood phospholipids  Platelet phospholipid | DHA  EPA  EPA  DPA  DHA  AA/EPA  DHA  EPA  AA/EPA | [[116](#_ENREF_116)] |
| Fish vs other meals | | Sequential meal study | 8 volunters (no details) | NMR | Urine | TMAO | [[117](#_ENREF_117)] |
| Fish vs. other proteins | | Six Sequential meal studies | 7 (1 man), 28-45y | NMR | Urine samples (4 per day) | TMAO  Creatine  3-Methylhistidine | [[118](#_ENREF_118)] |
| Fish | | Sequantial meal study | 6 healthy non-smoking men, average 32y | GC | Urine | TMAO | [[119](#_ENREF_119)] |
| Eel | | Single meal sequential study | 1 man, 45y | HPLC | Urine (pre- and post-prandial) | Carnosine | [[40](#_ENREF_40)] |
| Tuna | | Single meal sequential study | 1 man, 45y | HPLC | Urine (pre- and post-prandial) | Anserine  1-Methylhistidine  Carnosine | [[40](#_ENREF_40)] |
| Fish (Total lean + fatty) | | 3y prospective study | 214 diabetics (112 men), >20y | GC | Plasma phospholipids (fasting at baseline, non-fasting at follow-up) | EPA  DPA  DHA  Sum n-3  Sum n-6  PLN3-index  n-6/n-3 LC-PUFA | [[120](#_ENREF_120)] |
| Fatty Fish | | 3y Prospective study | 214 diabetics (112 men), >20y | GLC | Plasma phospholipids (fasting at baseline, non-fasting at follow-up)) | EPA  DHA  n-3 FA  n-6 FA  Ratio of n-6/n-3 PUFA | [[120](#_ENREF_120)] |
| Lean Fish | | 3y prospective study | 214 diabetics (112 men), >20y | GC | Plasma phospholipids (fasting at baseline, non-fasting at follow-up) | EPA  DHA  n-3 PUFA  n-6 PUFA | [[120](#_ENREF_120)] |
| Fish intake level | | 9 mo. Prospective study | 90 mothers, 63 infants (1 mo.) | GC | Fasting serum | DHA  EPA  n-6/n-3 PUFA ratio | [[121](#_ENREF_121)] |
| Fatty fish  Lean fish Total fish | | Cross-sectional study | 3003 (1421 men), 45-64y | GLC | Plasma Phospholipids | DHA  EPA  DHA  DHA | [[122](#_ENREF_122)] |
| Fish | | Cross-sectional study | 1369 non-smoking women | LC-MS/MS | Urine | CMPF  DHA  EPA  Choline(C22:6)  Choline(C20:5)  MG(22:6/0:0/0:0)  +1 unknown | [[41](#_ENREF_41)] |
| Dark fish | | Cross-sectional study | 1369 non-smoking women | LC-MS/MS | Urine | CMPF  DHA  EPA  DPA  Choline(C22:6)  Choline(C20:5)  MG(22:6/0:0/0:0)  SM(d18:2/18:1)  +2 unknowns | [[41](#_ENREF_41)] |
| Fish (as component of certain diet scores) | | Cross-sectional (Baseline of ATBC prospective study) | 1336 men, 50-69y | LC-MS | Serum | CMPF  DHA  DPA  EPA  PC(34:1)  MG(22:6/0:0/0:0)  FA(18:4n–3)  LPC(22:6)  N-acetyl-3-methylhistidine  3-Methylhistidine  Ergothioneine  Creatine | [[14](#_ENREF_14)] |
| Fish protein | | Cross-sectional | 1254 men and women, 40-80y | EA-IRMS | Serum | ^15^N/^14^N  ^13^C/^12^C | [[15](#_ENREF_15)] |
| Fish-based diet (oily fish) | | Cross-sectional study | 44 Yup’ik Eskimos (14 men), >14y | EA-IRMS | Hair | ^15^N/^14^N  ^13^C/^12^C | [[123](#_ENREF_123)] |
| Fish-based diet (oily fish) | | Cross-sectional study | 496 Yup’ik Eskimos (208 men), >14y | EA-IRMS | RBC | ^15^N/^14^N | [[124](#_ENREF_124)] |
| Fish (marine foods) vs. meat | | Cross-sectional study | 270 Chinese (135 men), average age ~51±12y | LC-MS, GC-MS | Fasting plasma | EPA DHA  CMPF  Hydroxyproline  PE(p36:5)  PC(36:5)  LPC(22:6)  LPE(22:6)  DHA-containing PCs, PEs and plasmalogens | [[125](#_ENREF_125)] |
| Fish-based diet (oily fish) | | Cross-sectional study | 230 Alaska natives (97 men), >14y | EA-IRMS | RBC | ^15^N/^14^N | [[126](#_ENREF_126)] |
| Dark fish | | Cross-sectional study | 1369 non-smoking women | LC-MS/MS | Urine | CMPF  DHA  EPA  DPA  Choline(C22:6)  Choline(C20:5)  MG(22:6/0:0/0:0)  SM(d18:2/18:1)  +2 unknowns | [[41](#_ENREF_41)] |
| Fish | | Cross-sectional study | 34 (16 men), 18-60y | ESI-MS/MS | Fasting serum and plasma | LPE(C18:2)  PE(C38:4) | [[127](#_ENREF_127)] |
| Fish intake (association) | | Cross-sectional (baseline in a case-control colorectal cancer study; Pearson correlations, FDR<0.1) | 253 subjects, 125 cases and 128 controls (77 men), average age 18-74y | LC-MS or  GC-MS  LC-MS | Serum  12h non-fasting urine | X - 02269 (X - 11469) (m/z= 255.1)  CMPF  DHA  X – 12644 (m/z= 524.3)  CMPF | [[42](#_ENREF_42)] |
| Salmon (frequency of intake) | | Cross-sectional study | 68 middle-aged Northumberland residents (33 men), | FIE-MS | 24-hour urine | 1-Methylhistidine  3-Methylhistidine  Creatine | [[128](#_ENREF_128)] |
| Fish-based diet (oily fish) | | Cross-sectional study | 44 Yup’ik Eskimos (14 men), >14y | EA-IRMS | Hair | ^15^N/^14^N  ^13^C/^12^C | [[123](#_ENREF_123)] |
| Biomarkers of mixed fish and fish oil intake | | | | | | | |
| Salmon fed fish oil or rape seed oil | | 6w parallel RCT | 58 CHD patients (50 men), 46-75y | GC | Fasting blood samples at baseline and after 6wk | n-3 PUFA  DHA  EPA  Ratio (n-3/n-6)PUFA | [[129](#_ENREF_129)] |
| Salmon vs fish oil | | 8w Crossover RCT with 6 mo washout | 33 healthy Serbians (18 men), 44-64y | Capillary GC | Fasting platelets and RBCs | EPA  DHA | [[130](#_ENREF_130)] |
| Fish free diet, fish diet, fish diet+fish oil  Fish oil | | 3 x 6w crossover RCT with 6w waskout  6w parallel trial | 12 healthy men, 18-58y  11 healthy men, 18-58y | Flame ionization capillary-GC | Fasting blood samples | EPA  DPA  DHA  EPA  DPA  DHA | [[131](#_ENREF_131)] |
| Fish diet followed by fish oil | | 2 x 6 mo. sequential study | 191 patients (110 men), >50y | Not specified | Plasma samples | EPA  EPA:AA | [[132](#_ENREF_132)] |
| Fish spread | | 3y prospective study | 214 diabetics (112 men), >20y | GC | Plasma phospholipids (fasting at baseline, non-fasting at follow-up) | EPA  DPA  DHA  n-3 PUFA  n-6 PUFA | [[120](#_ENREF_120)] |
| Biomarkers of fish oil intake | | | | | | | |
| Fish oil 8-10g vs. placebo pills before surgery | | 2-10d Parallel RCT | 564 cardiac surgery patients (406 men), average age 63y | GC | Plasma PL | EPA  DPA  DHA  n-3 PUFA | [[133](#_ENREF_133)] |
| Fish oil (0.33-4.5g/d) vs. flaxseed oil | | 14d Parallel RCT and dose-response | 303 young women | GLC | Plasma PC | DHA  EPA | [[134](#_ENREF_134)] |
| Fish oil, 2.2g/d vs olive oil | | 12w Parallel RCT | 141 healthy, middle-aged | Capillary GC | Fasting erythrocyte PL | EPA  DPA | [[135](#_ENREF_135)] |
| Fish oil equaling 1, 2 or 4 fish servings/w | | 12mo Parallel RCT | 128 (79 men), 20-80 with no habitual fish intake | GC | Fasting blood, (red blood cells, mononuclear cells, platelets, plasma phosphatidylcholine, triglycerides, cholesteryl esters, and nonesterified fatty acids), buccal cell, abdominal subcutaneous adipose tissue samples | EPA  DHA | [[136](#_ENREF_136)] |
| Fish oil with 5g DHA + EPA vs. corn oil | | 4mo Parallel RCT | 124 psoriasis patients (80 men), aged 19-74y | GC | Serum PL | EPA  DPA  DHA  Sum n-3 PUFA  Sum n-6 PUFA  AA:EPA  n-6 PUFA /n-3 PUFA | [[137](#_ENREF_137)] |
| Fish oil, 0-1800mg DHA+EPA | | 5 mo Parallel RCT | 115 healthy subjects (60 men), 20-45y | GC | Erythrocyte membrane | EPA  DPA  DHA | [[138](#_ENREF_138), [139](#_ENREF_139)] |
| Fish oil (1.8g/d) vs. krill oil (1.8g/d) and controls (none) | | 4w Parallel RCT | 113 healthy subjects, (36 men), average age 40. | GC-MS | Plasma | EPA  DHA  DPA | [[140](#_ENREF_140)] |
| Fish oil vs. flaxseed, hempseed, or sunflower oil (all 2g/d). | | 12w Parallel RCT | 86 healthy adults (34 men), 30-35y | GLC | Fasting plasma | EPA  DHA | [[141](#_ENREF_141)] |
| Fish oil 1.5g/d vs. corn oil | | 8w Crossover RCT with 8w washout | 84 subjects (29 men), divided by a polymorphism in eNOS (40 wt) | GC | Fasting plasma and PL | EPA  DPA | [[142](#_ENREF_142)] |
| Fish oil, 125mg/kg/d | | 90d Parallel intervention study | 65 kids with phenylketonuria and 30 healthy controls (17 boys), 1-11y | Capillary-GLC | Blood plasma samples (4-hour fasting) at baseline and after intervention: plasma phospholipid fatty acid | EPA  DPA  DHA  n-3 PUFA  n-6 PUFA  n-3 PUFA /n-6 PUFA | [[143](#_ENREF_143)] |
| Fish oil (0.6-3.6 g/d) vs. flaxseed oil (1g/d) | | 12w Parallel RCT | 62 healthy male fire-fighters, around 30-50y | GLC | Plasma and erythrocyte PL | EPA  DPA  DHA | [[144](#_ENREF_144)] |
| Fish oil (4g EPA + DPA/d) vs soybean oil control | | 12w Parallel, blinded trial | 60 subjects (46 men), >18y; treatment/ control 45/15) | Capillary-GC | Plasma (RBC) | Omega-3 index (for compliance) | [[145](#_ENREF_145)] |
| Fish oil, 0-9g/d | | 12 mo Parallel RCT + 6 mo washout | 58 male monks, around 40-70y | GLC | Fasting serum cholesteryl esters, erythrocytes, and subcutaneous adipose tissue | EPA  DPA  DHA | [[146](#_ENREF_146)] |
| Fish Oil, flaxseed oil, or corn oil | | 180d 3-arm Parallel RCT | 53 T2D patients (19 men), average age 63y | GC-MS and LC-MS | Fasting serum  Fasting serum | CMPF  PC-EPA  EPA  CMPF  EPA  PC-EPA  DHA  DPA | [[147](#_ENREF_147)] |
| Fish oil vs. olive oil, 2g/d | | 6w Parallel RCT | 50 overweight subjects, (24 men), 30-75y | GC | Fasting granulocytes and subcutaneous adipose tissue samples | EPA  DPA  DHA  Sn-3 PUFA  n-3 PUFA /n-6 PUFA | [[148](#_ENREF_148)] |
| Fish oil, 1.7g/d vs. safflower oil. | | 4w Parallel RCT | 41 healthy men, BMI>23., 18-30y | GC | Plasma PL and RBC | EPA  DHA  EPA+DHA | [[149](#_ENREF_149)] |
| Tuna oil, 0.44-1.9g/d, linseed oil, or placebo | | 12w 5-arm Parallel RCT | 40 healthy men, 18-39y | GC | Fasting plasma PL | EPA  DHA | [[150](#_ENREF_150)] |
| Fish oil ± vit. B12 | | 8w Parallel RCT | 30 healthy adults (sex unknown), 20-26y | TLC (for PL) and GLC (for FA methyl esters) | PL and plasma | EPA  DHA  n-3 PUFA  n-6 PUFA  n-3 PUFA /n-6 PUFA | [[151](#_ENREF_151)] |
| Fish oil (5g) vs. EPA (3g) | | 3w Parallel RCT | 29 healthy low-fish consumers (18 men), around 30y | TLC and GC | Serum and PL | EPA  DHA | [[152](#_ENREF_152)] |
| Fish oil (600mg/d) vs. olive oil (600mg/d) | | 2 x 4w Cross-over trial | 24 hyperlipoproteinemic patients (no details) | GC-MS | plasma  urine | CMPF  CMPF | [[153](#_ENREF_153)] |
| Fish oil (1.3g EPA + DHA/d) | | 12w Parallel RCT | 23 middle-aged hemodialysis patients (18 men) | GC | Fasting plasma  RBC | EPA  DPA  DHA  n-6/n-3  EPA+DHA  n-6 PUFA | [[154](#_ENREF_154)] |
| Fish Oil (EPA + DHA at 0, 0.25, 0.5 or 1g/d) | | Parallel intervention study, partially sequential. | 20 subjects (11 men), aged 18-35y | GC | Fingerprick whole blood  Erythrocytes,  PL, WB | DHA  DPA  EPA  % EPA+DHA  % n-3 HUFA/total HUFA  DHA:EPA | [[155](#_ENREF_155)] |
| Fish oil with 380mg n-3 LC-PUFAs | | 4w Parallel RCT | 17 healthy subjects (sex unknown), 25-69y | GLC | Plasma PL | EPA  DPA  n-3 PUFA | [[156](#_ENREF_156)] |
| Fish oil (2.8 g EPA and 1.4 g DHA) vs. corn oil ± isoflavones | | RCT meal study | 10 overweight men, >45y | GLC | Serum | EPA  DHA  n-3 PUFA | [[157](#_ENREF_157)] |
| Fish oil, 3g/d, no control group | | 12w Sequential study | 27 hemo-dialysis patients (13 men), average age 61y | GC | Non-fasting plasma | EPA  DPA | [[158](#_ENREF_158)] |
| Fish oil, 6g/d | | 3w Sequential intervention study | 19 healthy subjects (2 men), 22-53y | GLC | Fasting plasma and plasma PL | EPA  DPA  DHA | [[159](#_ENREF_159)] |
| Fish Oil (1.2g EPA, 0.8g DHA) | | 20w sequential intervention study (12w suppl., 8w washout) | 12 men, 18-25y | GC | Serum and RBC | EPA  DHA  DPA | [[160](#_ENREF_160)] |
| Fish Oil (2g EPA + 1g DHA/d), no controls | | 3mo Sequential intervention study | 10 healthy men, average age 23y | GC | Fasted serum samples | EPA  DPA  DHA | [[161](#_ENREF_161)] |
| Fish oil, 5g/d (1g n-3 PUFA) | | Sequential intervention study, duration not provided. | 10 neurologically disabled children (9 boys), 2-13y | GLC | Serum and PL | EPA  DHA  n-3 PUFA | [[162](#_ENREF_162)] |
| Cod liver oil, 10-40ml/d | | 20w Sequential study with dose increase for 12w, then decrease for 8w | 6 healthy men, 26-36y | GLC | Plasma free FAs at multiple time points  Plasma PL  Erythrocyte membranes | EPA  DHA  EPA  DHA  EPA  DHA | [[163](#_ENREF_163)] |
| Cod-liver oil vs. none | | 3y Prospective cohort study | 214 diabetics (112 men), >20y | GC | Plasma PL (fasting at baseline, non-fasting at follow-up) | EPA  DPA  DHA  n-3 PUFA | [[120](#_ENREF_120)] |
| Mixed seafood markers (including As-compounds) | | | | | | | |
| Lean seafood and fish (cod, Pollack, scallop) vs. non-seafood diet with fish oil | | 2 x 4w crossover RCT | 20 healthy subjects (7 men), average age 51y | NMR | Morning spot urine  Fasting serum | TMAO  DMA  TMAO | [[2](#_ENREF_2)] |
| Seafood (cod, farmed salmon, or blue mussels) vs potato | | RCT meal study | 39 healthy volunteers (10 men), 20-40y | HPLC-ICPMS | Urine over 72 hours | Total As  iAs  DMA  AsB  Non-AsB As | [[164](#_ENREF_164)] |
| Mixed seafood compared with other protein sources | | 2w sequential study (before-after) | 153 postmenopausal women, around 75y | isotope ratio MS | Fasting serum sample | ^15^N/^14^N  ^13^C/^12^C  ^34^S/^32^S | [[165](#_ENREF_165)] |
| Seafood (Tropical) vs. no fish | | 2w sequential intervention study | 12 healthy Australian natives (2 men), mean age 24y | Capillary-GLC | Plasma fatty acids | DHA  DPA  EPA  AA | [[166](#_ENREF_166)] |
| Seafood (lean): lemon sole, wolfish, crabs | | Single meal sequential intervention study after 4 days without fish or shellfish | 3 healthy volunteers (1 man), 23-50y | HPLC-ICPMS | Urine | Total As  AsB  DMA | [[167](#_ENREF_167)] |
| Seafood (comparing also environmental exposures and other food sources) | | 5mo Cohort study with weekly urine collections | 6 healthy Italian men | HPLC-ICPMS | Morning urine | AsB  iAs  DMA  MMA | [[168](#_ENREF_168)] |
| Seafood (fish, shellfish) intake estimates | | Bladder cancer case-control study (retrospective) in Michigan, USA | 343 local participants, 151 cases and 192 controls, (242 men) | HPLC-ICPMS | Spot urine | AsB | [[169](#_ENREF_169)] |
| Seafood and plant protein (as component of various diet scores) | | Cross-sectional (Baseline of ATBC prospective study) | 1336 men, 50-69y | LC-MS | Serum | CMPF  DHA  DPA  EPA  PC(34:1)  MG(22:6/0:0/0:0)  FA(18:4n–3)  LPC(22:6)  N-acetyl-3-methylhistidine  3-Methylhistidine  Creatine  Ergothioneine | [[14](#_ENREF_14)] |
| Seafood (fried and non-fried fish, raw shellfish) | | Cross-sectional study (baseline from MESA cohort) | 900 participants (387 men), average age ~60y | GC | Plasma PL | EPA  DHA  DHA+ EPA | [[170](#_ENREF_170)] |
| Seafood (fish, raw oysters, shellfish) | | Cross-sectional study (baseline of NHANES 2003-2004) | 788 participants (417 men), >20y | HPLC-ICP-DRC-MS | Spot urine | Total As  DMA  AsB  Non-AsB As | [[171](#_ENREF_171)] |
| Mixed fish and shellfish | | Cross-sectional study | 270 Chinese (135 men), average age ~51±12y | - LC-MS, GC-MS | Fasting plasma | Hydroxyproline  Valine  Lysine  EPA DHA  CMPF  PE(P-36:5)  PC(36:5)  LPC(22:6)  LPE(22:6)  DHA-containing PCs, PEs, and plasmalogens | [[125](#_ENREF_125)] |
| Japanese coastal diet baseline values | | Cross-sectional study (baseline of astaxanthin intervention) | 20-30 healthy male subjects, 40-69y | LC-MS | Fasting heparin plasma (20 subjects) and erythrocytes (30 subjects) | Astaxanthin | [[172](#_ENREF_172), [173](#_ENREF_173)] |
| Shellfish markers (including As-compounds) | | | | | | | |
| Shellfish (oysters, clams, crabs, mussels, squid and shrimps) | | 3 x 21w Parallel RCT with sequential diets where each period contained only one shellfish | 18 men, 23-38y | GC | Plasma and erythrocyte membrane | EPA  DHA | [[174](#_ENREF_174)] |
| Blue mussels | | Single meal intervention study | 4 men, 5 women | HPLC-ICPMS | Urine over 72 hr | AsB  DMA+As(V)  As sugars  Sum of unknowns As compounds | [[175](#_ENREF_175)] |
| Shellfish | | Cross-sectional study | 1369 non-smoking women | LC-MS/MS | Urine | CMPF | [[41](#_ENREF_41)] |
| Shellfish | | Cross-sectional analysis of nested case-control study | 502 CRC cases and controls (281 men), 55-74y | LC-MS/MS  GC-MS | Serum | CMPF | [[91](#_ENREF_91)] |
| Shellfish | | Cross-sectional study | 270 Chinese (135 men), average age 51±12y | - LC-MS, GC-MS | Fasting plasma | PE(P-36:4) | [[125](#_ENREF_125)] |
| Shellfish | | Cross-sectional analysis of samples from a case-control colorectal cancer study | 253 subjects, 125 cases and 128 controls (77 men), 18-77y | LC-MS  GC-MS   - LC-MS or GC-MS | Serum  12h non-fasting overnight urine | CMPF  + one unknown (POS m/z= 255.1)  Lysine  2-aminoethylphosphonate  2-hydroxybutyrate  Creatine  3-hydroxybutyrate  Taurine  N-acetylglycine  Alpha-hydroxyisovalerate  Sulforaphane-cysteine | [[42](#_ENREF_42)] |

Abbreviations: 1-OHP, 1-hydroxypyrene; 1-OHPG, 1-hydroxypyrene glucuronide; 1-MH, 1-methylhistidine (π-methylhistidine); 3-MH, 3-methylhistidine (τ-methylhistidine) ; AA, arachidonic acid; AαC, 2-amino-9H-pyrido[2,3-b]indole; AsB, arsenobetaine; ATNC, apparent total nitroso compounds; CMPF, 3-​carboxy-​4-​methyl-​5-​propyl-​2-​furanepropanoic acid; DHA, docosahexaenoic acid; DMA, dimethylamine; DHM-MA, ; DPA, docosapentaenoic acid; EA-IRMS: elemental analyzer coupled online via a conflow interface with an isotope ratio MS; EPA, eicosapentaenoic acid; FA, fatty acid; FIE-MS: flow infusion electrospray–ionization mass spectrometry; FLD: fluorence detector; FLU, fluoranthene; GC-MS: gas chromatography coupled with mass spectrometry; HPLC: high pressure liquid chromatography; IAC: immunoaffinity chromatography; iAs, inorganic As; IEC: ion-exchange chromatography; LC-MS: liquid chromatography coupled with mass spectrometry; LPC(x:y), lysophosphatidylcholine with one esterified fatty acid having in total x carbons and y double bonds; LPE(x:y), phosphatidylethanolamine with one esterified fatty acid having in total x carbons and y double bonds; MeIQx, 2-amino-3,8-dimethylimidazo[4,5-f]quinoxaline; MG(x:y/0:0,0:0), monoacylglycerol with a fatty acid of x carbons and y double bonds in position 1; MMA, monomethylamine; NAP, naphtalene; NMR: nuclear magnetic resonance spectrometry; PC(x:y), phosphatidylcholine with two esterified fatty acids having in total x carbons and y double bonds; PC-EPA, PC-containing esterified EPA; PE(x:y), phosphatidylethanolamine with two esterified fatty acids having in total x carbons and y double bonds; PHE, phenanthrene; PhIP, 2‑Amino-1-methyl-6-phenylimidazo[4,5‑b]pyridine; PL, phospholipids; PUFA, polyunsaturated fatty acids (n-3 and/or n-6); SFS : synchronous fluorescence spectroscopy; SM(x:y), sphingomyelin with two esterified fatty acids having in total x carbons and y double bonds; TEA: thermal energy analyzer; TLC: thin layer chromatography; TMA, trimethyl amine; TMAO, trimethylamineoxide; Trp-P-1, 3-amino-1,4-dimethyl-5H-pyrido[3,4-b]indole; Trp-P-2, 3-amino-1-methyl-5H-pyrido[3,4-b]indole; WB, whole blood; * free and conjugated metabolites

1. Altorf-van der Kuil W, Brink EJ, Boetje M, Siebelink E, Bijlsma S, Engberink MF, et al. Identification of biomarkers for intake of protein from meat, dairy products and grains: a controlled dietary intervention study. British Journal of Nutrition. 2013;110(5):810-22.

2. Schmedes M, Aadland EK, Sundekilde UK, Jacques H, Lavigne C, Graff IE, et al. Lean-seafood intake decreases urinary markers of mitochondrial lipid and energy metabolism in healthy subjects: Metabolomics results from a randomized crossover intervention study. Molecular nutrition & food research. 2016;60(7):1661-72.

3. Vincent A, Savolainen OI, Sen P, Carlsson NG, Almgren A, Lindqvist H, et al. Herring and chicken/pork meals lead to differences in plasma levels of TCA intermediates and arginine metabolites in overweight and obese men and women. Molecular nutrition & food research. 2017;61(3).

4. Kuhnle GGC, Joosen A, Kneale CJ, O'Connell TC. Carbon and nitrogen isotopic ratios of urine and faeces as novel nutritional biomarkers of meat and fish intake. European Journal of Nutrition. 2013;52(1):389-95.

5. Stella C, Beckwith-Hall B, Cloarec O, Holmes E, Lindon JC, Powell J, et al. Susceptibility of Human Metabolic Phenotypes to Dietary Modulation. Journal of Proteome Research. 2006;5(10):2780-8..

6. Bertram HC, Hoppe C, Petersen BO, Duus JØ, Mølgaard C, Michaelsen KF. An NMR-based metabonomic investigation on effects of milk and meat protein diets given to 8-year-old boys. British Journal of Nutrition. 2007;97(4):758-63.

7. Block WD, Hubbard RW, Steele BF. Excretion of Histidine and Histidine Derivatives by Human Subjects Ingesting Protein from Different Sources. The Journal of Nutrition. 1965;85(4):419-25.

8. Lukaski HC, Mendez J, Buskirk ER, Cohn SH. Relationship between endogenous 3-methylhistidine excretion and body composition. The American journal of physiology. 1981;240(3):E302-7.

9. Elia M, Carter A, Bacon S, Smith R. The Effect of 3-Methylhistidine in Food on its Urinary Excretion in Man. Clinical Science. 1980;59(6):509-11.

10. Evered DF, Harvey MS, Luck LJ, Solari ME. The relationship between urinary taurine excretion and the intake of protein-rich foods. Life Sciences. 1969;8(12):601-5.

11. Datta SP, Harris H. Dietary origin of urinary methylhistidine. Nature. 1951;168(4268):296-7. PubMed PMID: 14875074.

12. Pallister T, Jennings A, Mohney RP, Yarand D, Mangino M, Cassidy A, et al. Characterizing blood metabolomics profiles associated with self-reported food intakes in female twins. PLoS ONE. 2016;11(6).

13. Pallister T, Sharafi M, Lachance G, Pirastu N, Mohney RP, MacGregor A, et al. Food Preference Patterns in a UK Twin Cohort. Twin Research and Human Genetics. 2015;18(6):793-805.

14. Playdon MC, Moore SC, Derkach A, Reedy J, Subar AF, Sampson JN, et al. Identifying biomarkers of dietary patterns by using metabolomics. The American journal of clinical nutrition. 2017;105(2):450-65.

15. Patel PS, Cooper AJM, O'Connell TC, Kuhnle GGC, Kneale CK, Mulligan AM, et al. Serum carbon and nitrogen stable isotopes as potential biomarkers of dietary intake and their relation with incident type 2 diabetes: The EPIC-Norfolk study. American Journal of Clinical Nutrition. 2014;100(2):708-18.

16. Lau CE, Siskos AP, Maitre L, Robinson O, Athersuch TJ, Want EJ, et al. Determinants of the urinary and serum metabolome in children from six European populations. BMC medicine. 2018;16(1):202. Epub 2018/11/09.

17. Fraser GE, Jaceldo-Siegl K, Henning SM, Fan J, Knutsen SF, Haddad EH, et al. Biomarkers of Dietary Intake Are Correlated with Corresponding Measures from Repeated Dietary Recalls and Food-Frequency Questionnaires in the Adventist Health Study-2. Journal of Nutrition. 2016;146(3):586-94.

18. Schmidt JA, Rinaldi S, Ferrari P, Carayol M, Achaintre D, Scalbert A, et al. Metabolic profiles of male meat eaters, fish eaters, vegetarians, and vegans from the EPIC-Oxford cohort. American Journal of Clinical Nutrition. 2015;102(6):1518-26.

19. Kruger R, Merz B, Rist MJ, Ferrario PG, Bub A, Kulling SE, et al. Associations of current diet with plasma and urine TMAO in the KarMeN study: direct and indirect contributions. Molecular nutrition & food research. 2017;61(11).

20. Mitry P, Wawro N, Rohrmann S, Giesbertz P, Daniel H, Linseisen J. Plasma concentrations of anserine, carnosine and pi-methylhistidine as biomarkers of habitual meat consumption. European journal of clinical nutrition. 2018. Epub 2018/07/19.

21. Xu J, Yang S, Cai S, Dong J, Li X, Chen Z. Identification of biochemical changes in lactovegetarian urine using 1H NMR spectroscopy and pattern recognition. Analytical and Bioanalytical Chemistry. 2010;396(4):1451-63.

22. Delanghe J, De Slypere JP, De Buyzere M, Robbrecht J, Wieme R, Vermeulen A. Normal reference values for creatine, creatinine, and carnitine are lower in vegetarians. Clinical Chemistry. 1989;35(8):1802.

23. Petzke KJ, Boeing H, Klaus S, Metges CC. Carbon and nitrogen stable isotopic composition of hair protein and amino acids can be used as biomarkers for animal-derived dietary protein intake in humans. Journal of Nutrition. 2005;135(6):1515-20.

24. Myint T, Fraser GE, Lindsted KD, Knutsen SF, Hubbard RW, Bennett HW. Urinary 1-methylhistidine is a marker of meat consumption in Black and in White California Seventh-day Adventists. American Journal of Epidemiology. 2000;152(8):752-5.

25. Petzke KJ, Boeing H, Metges CC. Choice of dietary protein of vegetarians and omnivores is reflected in their hair protein 13C and 15N abundance. Rapid Communications in Mass Spectrometry. 2005;19(11):1392-400.

26. Bol R, Pflieger C. Stable isotope (13C, 15N and 34S) analysis of the hair of modern humans and their domestic animals. Rapid Communications in Mass Spectrometry. 2002;16(23):2195-200.

27. O'Connell TC, Hedges REM. Investigations into the effect of diet on modern human hair isotopic values. American Journal of Physical Anthropology. 1999;108(4):409-25.

28. Wang Z, Bergeron N, Levison BS, Li XS, Chiu S, Jia X, et al. Impact of chronic dietary red meat, white meat, or non-meat protein on trimethylamine N-oxide metabolism and renal excretion in healthy men and women. European heart journal. 2018.

29. Bingham SA, Hughes R, Cross AJ. Effect of White Versus Red Meat on Endogenous N-Nitrosation in the Human Colon and Further Evidence of a Dose Response. The Journal of Nutrition. 2002;132(11):3522S-5S.

30. Cross AJ, Major JM, Sinha R. Urinary biomarkers of meat consumption. Cancer Epidemiology Biomarkers and Prevention. 2011;20(6):1107-11.

31. Petzke KJ, Lemke S. Hair protein and amino acid C-13 and N-15 abundances take more than 4 weeks to clearly prove influences of animal protein intake in young women with a habitual daily protein consumption of more than 1 g per kg body weight. Rapid Communications in Mass Spectrometry. 2009;23(16):2411-20.

32. Cho CE, Taesuwan S, Malysheva OV, Bender E, Tulchinsky NF, Yan J, et al. Trimethylamine-N-oxide (TMAO) response to animal source foods varies among healthy young men and is influenced by their gut microbiota composition: A randomized controlled trial. Mol Nutr Food Res. 2017;61(1). Epub 2016/07/06.

33. Rådjursöga M, Karlsson GB, Lindqvist HM, Pedersen A, Persson C, Pinto RC, et al. Metabolic profiles from two different breakfast meals characterized by 1H NMR-based metabolomics. Food chemistry. 2017;231:267-74.

34. Ross AB, Svelander C, Undeland I, Pinto R, Sandberg A-S. Herring and Beef Meals Lead to Differences in Plasma 2-Aminoadipic Acid, β-Alanine, 4-Hydroxyproline, Cetoleic Acid, and Docosahexaenoic Acid Concentrations in Overweight Men. The Journal of Nutrition. 2015;145(11):2456-63.

35. Yeum K-J, Orioli M, Regazzoni L, Carini M, Rasmussen H, Russell RM, et al. Profiling histidine dipeptides in plasma and urine after ingesting beef, chicken or chicken broth in humans. Amino Acids. 2010;38(3):847-58.

36. Pierre F, Peiro G, Tache S, Cross AJ, Bingham SA, Gasc N, et al. New marker of colon cancer risk associated with heme intake: 1,4-dihydroxynonane mercapturic acid. Cancer epidemiology, biomarkers & prevention : a publication of the American Association for Cancer Research, cosponsored by the American Society of Preventive Oncology. 2006;15(11):2274-9.

37. Sjolin J, Hjort G, Friman G, Hambraeus L. Urinary excretion of 1-methylhistidine: a qualitative indicator of exogenous 3-methylhistidine and intake of meats from various sources. Metabolism: clinical and experimental. 1987;36(12):1175-84.

38. Park YJ, Volpe SL, Decker EA. Quantitation of Carnosine in Humans Plasma after Dietary Consumption of Beef. Journal of Agricultural and Food Chemistry. 2005;53(12):4736-9.

39. Huszar G, Golenwsky G, Maiocco J, Davis E. Urinary 3-methylhistidine excretion in man: the role of protein-bound and soluble 3-methylhistidine. The British journal of nutrition. 1983;49(3):287-94.

40. Abe H, Emiko O, Hideo S, Akio M, Shohei Y. Human urinary excretion of l-histidine-related compounds after ingestion of several meats and fish muscle. International Journal of Biochemistry. 1993;25(9):1245-9.

41. Wang Y, Gapstur SM, Carter BD, Hartman TJ, Stevens VL, Gaudet MM, et al. Untargeted metabolomics identifies novel potential biomarkers of habitual food intake in a cross-sectional study of postmenopausal women. Journal of Nutrition. 2018;148(6):932-43.

42. Playdon MC, Sampson JN, Cross AJ, Sinha R, Guertin KA, Moy KA, et al. Comparing metabolite profiles of habitual diet in serum and urine. American Journal of Clinical Nutrition. 2016;104(3):776-89.

43. O'Sullivan A, Gibney MJ, Brennan L. Dietary intake patterns are reflected in metabolomic profiles: Potential role in dietary assessment studies. American Journal of Clinical Nutrition. 2011;93(2):314-21.

44. Cheung W, Keski-Rahkonen P, Assi N, Ferrari P, Freisling H, Rinaldi S, et al. A metabolomic study of biomarkers of meat and fish intake. American Journal of Clinical Nutrition. 2017;105(3):600-8.

45. Kochlik B, Gerbracht C, Grune T, Weber D. The Influence of Dietary Habits and Meat Consumption on Plasma 3-Methylhistidine-A Potential Marker for Muscle Protein Turnover. Molecular nutrition & food research. 2018;62(9):e1701062.

46. Yin X, Gibbons H, Rundle M, Frost G, McNulty BA, Nugent AP, et al. Estimation of Chicken Intake by Adults Using Metabolomics-Derived Markers. J Nutr. 2017. Epub 2017/08/11. doi: 10.3945/jn.117.252197. PubMed PMID: 28794208.

47. Le Marchand L, Yonemori K, White KK, Franke AA, Wilkens LR, Turesky RJ. Dose validation of PhIP hair level as a biomarker of heterocyclic aromatic amines exposure: a feeding study. Carcinogenesis. 2016;37(7):685-91.

48. Turesky RJ, Liu L, Gu D, Yonemori KM, White KK, Wilkens LR, et al. Biomonitoring the Cooked Meat Carcinogen 2-Amino-1-Methyl-6-Phenylimidazo 4,5-b Pyridine in Hair: Impact of Exposure, Hair Pigmentation, and Cytochrome P450 1A2 Phenotype. Cancer Epidemiology Biomarkers & Prevention. 2013;22(3):356-64.

49. Turesky RJ, White KK, Wilkens LR, Le ML. Caffeine Cytochrome P450 1A2 Metabolic Phenotype Does Not Predict the Metabolism of Heterocyclic Aromatic Amines in Humans. ChemResToxicol. 2015;28(8):1603-15.

50. Guo J, Yonemori K, Le ML, Turesky RJ. Method to Biomonitor the Cooked Meat Carcinogen 2-Amino-1-methyl-6-phenylimidazo[4,5-b]pyridine in Dyed Hair by Ultra-Performance Liquid Chromatography-Orbitrap High Resolution Multistage Mass Spectrometry. AnalChem. 2015;87(12):5872-7.

51. Strickland PT, Qian Z, Friesen MD, Rothman N, Sinha R. Metabolites of 2-amino-1-methyl-6-phenylimidazo(4,5-b)pyridine (PhIP) in human urine after consumption of charbroiled or fried beef. MutatRes. 2002;506-507:163-73.

52. Friesen MD, Rothman N, Strickland PT. Concentration of 2-amino-1-methyl-6-phenylimidazo(4,5-b)pyridine (PhIP) in urine and alkali-hydrolyzed urine after consumption of charbroiled beef. Cancer Letters. 2001;173(1):43-51.

53. Kang DH, Rothman N, Poirier MC, Greenberg A, Hsu CH, Schwartz BS, et al. Interindividual differences in the concentration of 1-hydroxypyrene-glucuronide in urine and polycyclic aromatic hydrocarbon-DNA adducts in peripheral white blood cells after charbroiled beef consumption. Carcinogenesis. 1995;16(5):1079-85.

54. Stillwell WG, Kidd LCR, Wishnok JS, Tannenbaum SR, Sinha R. Urinary excretion of unmetabolized and phase II conjugates of 2-amino-1-methyl-6-phenylimidazo 4,5-b pyridine and 2-amino-3,8-dimethylimidazo 4,5-f quinoxaline in humans: Relationship to cytochrome P4501A2 and N-acetyltransferase activity. Cancer Research. 1997;57(16):3457-64.

55. Stillwell WG, Turesky RJ, Sinha R, Skipper PL, Tannenbaum SR. Biomonitoring of heterocyclic aromatic amine metabolites in human urine. Cancer Letters. 1999;143(2):145-8.

56. Stillwell WG, Turesky RJ, Sinha R, Tannenbaum SR. N-oxidative metabolism of 2-amino-3,8-dimethylimidazo[4,5-f]quinoxaline (MeIQx) in humans: excretion of the N2-glucuronide conjugate of 2-hydroxyamino-MeIQx in urine. Cancer Res. 1999;59(20):5154-9.

57. Stillwell WG, Sinha R, Tannenbaum SR. Excretion of the N(2)-glucuronide conjugate of 2-hydroxyamino-1-methyl-6-phenylimidazo[4,5-b]pyridine in urine and its relationship to CYP1A2 and NAT2 activity levels in humans. Carcinogenesis. 2002;23(5):831-8.

58. Strickland PT, Qian Z, Friesen MD, Rothman N, Sinha R. Measurement of 2-amino-1-methyl-6-phenylimidazo(4,5-b) pyridine (PhIP) in acid-hydrolyzed urine by high-performance liquid chromatography with fluorescence detection. Biomarkers. 2001;6(5):313-25.

59. Walters DG, Young PJ, Agus C, Knize MG, Boobis AR, Gooderham NJ, et al. Cruciferous vegetable consumption alters the metabolism of the dietary carcinogen 2-amino-1-methyl-6-phenylimidazo[4,5-b]pyridine (PhIP) in humans. Carcinogenesis. 2004;25(9):1659-69.

60. Murray S, Lake BG, Gray S, Edwards AJ, Springall C, Bowey EA, et al. Effect of cruciferous vegetable consumption on heterocyclic aromatic amine metabolism in man. Carcinogenesis. 2001;22(9):1413-20.

61. Reistad R, Rossland OJ, Latva-Kala KJ, Rasmussen T, Vikse R, Becher G, et al. Heterocyclic aromatic amines in human urine following a fried meat meal. Food and Chemical Toxicology. 1997;35(10-11):945-55.

62. Felton JS, Knize MG, Salmon CP, Malfatti MA, Kulp KS. Human exposure to heterocyclic amine food mutagens/carcinogens: Relevance to breast cancer. Environmental and Molecular Mutagenesis. 2002;39(2-3):112-8.

63. Kataoka H, Inoue T, Ikekita N, Saito K. Development of exposure assessment method based on the analysis of urinary heterocyclic amines as biomarkers by on-line in-tube solid-phase microextraction coupled with liquid chromatography-tandem mass spectrometry Microextraction Techniques. Analytical and Bioanalytical Chemistry. 2014;406(8):2171-8.

64. Kim D, Lee YJ, Ryu HY, Lee JH, Kim HK, Kim E, et al. Genetic polymorphisms in metabolism of 2-amino-1-methyl-6-phenylimidazo[4,5-b] pyridine. JApplToxicol. 2013;33(1):63-70.

65. Kulp KS, Knize MG, Fowler ND, Salmon CP, Felton JS. PhIP metabolites in human urine after consumption of well-cooked chicken. Journal of Chromatography B-Analytical Technologies in the Biomedical and Life Sciences. 2004;802(1):143-53.

66. Zhang Y, Ding J, Shen G, Zhong J, Wang C, Wei S, et al. Dietary and inhalation exposure to polycyclic aromatic hydrocarbons and urinary excretion of monohydroxy metabolites - A controlled case study in Beijing, China. Environmental Pollution. 2014;184:515-22.

67. Frandsen H. Biomonitoring of urinary metabolites of 2-amino-1-methyl-6-phenylimidazo 4,5-b pyridine (PhIP) following human consumption of cooked chicken. Food and Chemical Toxicology. 2008;46(9):3200-5.

68. Knize MG, Kulp KS, Malfatti MA, Salmon CP, Felton JS. Liquid chromatography-tandem mass spectrometry method of urine analysis for determining human variation in carcinogen metabolism. Journal of Chromatography A. 2001;914(1-2):95-103.

69. van Maanen JM, Moonen EJ, Maas LM, Kleinjans JC, van Schooten FJ. Formation of aromatic DNA adducts in white blood cells in relation to urinary excretion of 1-hydroxypyrene during consumption of grilled meat. Carcinogenesis. 1994;15(10):2263-8.

70. Li Z, Romanoff L, Bartell S, Pittman EN, Trinidad DA, McClean M, et al. Excretion Profiles and Half-Lives of Ten Urinary Polycyclic Aromatic Hydrocarbon Metabolites after Dietary Exposure. Chemical Research in Toxicology. 2012;25(7):1452-61.

71. Chien YC, Yeh CT. Amounts and proportion of administered pyrene dose excreted as urinary 1-hydroxypyrene after dietary exposure to polycyclic aromatic hydrocarbons. Archives of Toxicology. 2010;84(10):767-76.

72. Chien YC, Yeh CT. Excretion kinetics of urinary 3-hydroxybenzo[a]pyrene following dietary exposure to benzo[a]pyrene in humans. Arch Toxicol. 2012;86(1):45-53.

73. Busquets R, Frandsen H, Jonsson JA, Puignou L, Teresa Galceran M, Skog K. Biomonitoring of Dietary Heterocyclic Amines and Metabolites in Urine by Liquid Phase Microextraction: 2-Amino-1-methyl-6-phenylimidazo 4,5-b pyridine (PhIP), a Possible Biomarker of Exposure to Dietary PhIP. Chemical Research in Toxicology. 2013;26(2):233-40.

74. Kulp KS, Knize MG, Malfatti MA, Salmon CP, Felton JS. Identification of urine metabolites of 2-amino-1-methyl-6-phenylimidazo 4,5-b pyridine following consumption of a single cooked chicken meal in humans. Carcinogenesis. 2000;21(11):2065-72.

75. Turesky RJ, Gross GA, Stillwell WG, Skipper PL, Tannenbaum SR. Species differences in metabolism of heterocyclic aromatic amines, human exposure, and biomonitoring. Environmental Health Perspectives. 1994;102(SUPPL. 6):47-51.

76. Vanhaecke L, Knize MG, Noppe H, De Brabander H, Verstraete W, Van de Wiele T. Intestinal bacteria metabolize the dietary carcinogen 2-amino-1-methyl-6-phenylimidazo 4,5-b pyridine following consumption of a single cooked chicken meal in humans. Food and Chemical Toxicology. 2008;46(1):140-8.

77. Viau C, Diakite A, Ruzgyte A, Tuchweber B, Blais C, Bouchard M, et al. Is 1-hydroxypyrene a reliable bioindicator of measured dietary polycyclic aromatic hydrocarbon under normal conditions? Journal of Chromatography B-Analytical Technologies in the Biomedical and Life Sciences. 2002;778(1-2):165-77.

78. Gunier RB, Reynolds P, Hurley SE, Yerabati S, Hertz A, Strickland P, et al. Estimating exposure to polycyclic aromatic hydrocarbons: A comparison of survey, biological monitoring, and geographic information system-based methods. Cancer Epidemiology Biomarkers & Prevention. 2006;15(7):1376-81.

79. Ji H, Yu MC, Stillwell WG, Skipper PL, Ross RK, Henderson BE, et al. Urinary excretion of 2-amino-3,8-dimethylimidazo-[4,5-f]quinoxaline in white, black, and Asian men in Los Angeles County. Cancer EpidemiolBiomarkers Prev. 1994;3(5):407-11.

80. La Creis R, Kidd LC, Stillwell WG, Yu MC, Wishnok JS, Skipper PL, et al. Urinary excretion of 2-amino-1-methyl-6-phenylimidazo[4,5-b]pyridine (PhIP) in White, African-American, and Asian-American men in Los Angeles County. Cancer EpidemiolBiomarkers Prev. 1999;8(5):439-45.

81. Islami F, Boffetta P, van Schooten FJ, Strickland P, Phillips DH, Pourshams A, et al. Exposure to Polycyclic Aromatic Hydrocarbons Among Never Smokers in Golestan Province, Iran, an Area of High Incidence of Esophageal Cancer - a Cross-Sectional Study with Repeated Measurement of Urinary 1-OHPG in Two Seasons. Frontiers in oncology. 2012;2:14-.

82. Magagnotti C, Orsi F, Bagnati R, Celli N, Rotilio D, Fanelli R, et al. Effect of diet on serum albumin and hemoglobin adducts of 2-amino-1-methyl-6-phenylimidazo[4,5-b]pyridine (PhIP) in humans. IntJCancer. 2000;88(1):1-6.

83. Kobayashi M, Hanaoka T, Hashimoto H, Tsugane S. 2-Amino-1-methyl-6-phenylimidazo[4,5-b]pyridine (PhIP) level in human hair as biomarkers for dietary grilled/stir-fried meat and fish intake. MutatRes. 2005;588(2):136-42.

84. Kobayashi M, Hanaoka T, Tsugane S. Validity of a self-administered food frequency questionnaire in the assessment of heterocyclic amine intake using 2-amino-l-methyl-6-phenylimidazo 4,5-b pyridine (PhIP) levels in hair. Mutation Research-Genetic Toxicology and Environmental Mutagenesis. 2007;630(1-2):14-9.

85. Reistad R, Nyholm SH, Haug LS, Becher G, Alexander J. 2-amino-1-methyl-6-phenylimidazo(4,5-beta)pyridine (PhlP), in human hair as biomarker for dietary exposure. Proceedings of the American Association for Cancer Research Annual Meeting. 1999;40:151-. PubMed PMID: BCI:BCI199900183642.

86. Bessette EE, Yasa I, Dunbar D, Wilkens LR, Marchand LL, Turesky RJ. Biomonitoring of Carcinogenic Heterocyclic Aromatic Amines in Hair: A Validation Study. Chemical Research in Toxicology. 2009;22(8):1454-63.

87. Joosen AM, Kuhnle GG, Aspinall SM, Barrow TM, Lecommandeur E, Azqueta A, et al. Effect of processed and red meat on endogenous nitrosation and DNA damage. Carcinogenesis. 2009;30(8):1402-7. Epub 2009/06/06.

88. Pierre FH, Martin OC, Santarelli RL, Tache S, Naud N, Gueraud F, et al. Calcium and alpha-tocopherol suppress cured-meat promotion of chemically induced colon carcinogenesis in rats and reduce associated biomarkers in human volunteers. Am J Clin Nutr. 2013;98(5):1255-62. Epub 2013/09/13.

89. Stich HF, Hornby AP, Dunn BP. The effect of dietary factors on nitrosoproline levels in human urine. International journal of cancer. 1984;33(5):625-8.

90. Buratti M, Pellegrino O, Brambilla G, Colombi A. Urinary excretion of 1-hydroxypyrene as a biomarker of exposure to polycyclic aromatic hydrocarbons from different sources. Biomarkers. 2000;5(5):368-81.

91. Guertin KA, Moore SC, Sampson JN, Huang WY, Xiao Q, Stolzenberg-Solomon RZ, et al. Metabolomics in nutritional epidemiology: identifying metabolites associated with diet and quantifying their potential to uncover diet-disease relations in populations. American Journal of Clinical Nutrition. 2014;100(1):208-17.

92. Altmaier E, Kastenmuller G, Romisch-Margl W, Thorand B, Weinberger KM, Illig T, et al. Questionnaire-based self-reported nutrition habits associate with serum metabolism as revealed by quantitative targeted metabolomics. European journal of epidemiology. 2011;26(2):145-56. Epub 2010/12/01.

93. Ji H, Yu MC, Stillwell WG, Skipper PL, Ross RK, Henderson BE, et al. Urinary excretion of 2-amino-3,8-dimethylimidazo-[4,5-f]quinoxaline in white, black, and Asian men in Los Angeles County. Cancer epidemiology, biomarkers & prevention : a publication of the American Association for Cancer Research, cosponsored by the American Society of Preventive Oncology. 1994;3(5):407-11.

94. Biltoft-Jensen A, Damsgaard CT, Andersen R, Ygil KH, Andersen EW, Ege M, et al. Accuracy of self-reported intake of signature foods in a school meal intervention study: Comparison between control and intervention period. British Journal of Nutrition. 2015;114(4):635-44.

95. Handeland K, Skotheim S, Baste V, Graff IE, Froyland L, Lie O, et al. The effects of fatty fish intake on adolescents' nutritional status and associations with attention performance: results from the FINS-TEENS randomized controlled trial. Nutrition journal. 2018;17(1):30.

96. Andersen M-BS, Rinnan A, Manach C, Poulsen SK, Pujos-Guillot E, Larsen TM, et al. Untargeted Metabolomics as a Screening Tool for Estimating Compliance to a Dietary Pattern. Journal of Proteome Research. 2014;13(3):1405-18.

97. Uusitupa M, Hermansen K, Savolainen MJ, Schwab U, Kolehmainen M, Brader L, et al. Effects of an isocaloric healthy Nordic diet on insulin sensitivity, lipid profile and inflammation markers in metabolic syndrome - a randomized study (SYSDIET). Journal of Internal Medicine. 2013;274(1):52-66.

98. Zhang J, Wang C, Li L, Man Q, Meng L, Song P, et al. Dietary inclusion of salmon, herring and pompano as oily fish reduces CVD risk markers in dyslipidaemic middle-aged and elderly Chinese women. British Journal of Nutrition. 2012;108(8):1455-65.

99. Hanhineva K, Lankinen MA, Pedret A, Schwab U, Kolehmainen M, Paananen J, et al. Nontargeted Metabolite Profiling Discriminates Diet-Specific Biomarkers for Consumption of Whole Grains, Fatty Fish, and Bilberries in a Randomized Controlled Trial. Journal of Nutrition. 2015;145(1):7-17.

100. Bondia-Pons I, Martinez JA, de la Iglesia R, Lopez-Legarrea P, Poutanen K, Hanhineva K, et al. Effects of short- and long-term Mediterranean-based dietary treatment on plasma LC-QTOF/MS metabolic profiling of subjects with metabolic syndrome features: The Metabolic Syndrome Reduction in Navarra (RESMENA) randomized controlled trial. Molecular nutrition & food research. 2015;59(4):711-28.

101. Hansen AL, Dahl L, Olson G, Thornton D, Graff IE, Froyland L, et al. Fish Consumption, Sleep, Daily Functioning, and Heart Rate Variability. Journal of Clinical Sleep Medicine. 2014;10(5):567-75.

102. Zhang J, Wang C, Li L, Man Q, Song P, Meng L, et al. Inclusion of Atlantic salmon in the Chinese diet reduces cardiovascular disease risk markers in dyslipidemic adult men. Nutrition Research. 2010;30(7):447-54.

103. Schwab US, Lankinen MA, de Mello VD, Manninen SM, Kurl S, Pulkki KJ, et al. Camelina Sativa Oil, but not Fatty Fish or Lean Fish, Improves Serum Lipid Profile in Subjects with Impaired Glucose Metabolism-A Randomized Controlled Trial. Molecular nutrition & food research. 2018;62(4). 104. Lindqvist HM, Langkilde AM, Undeland I, Sandberg AS. Herring (Clupea harengus) intake influences lipoproteins but not inflammatory and oxidation markers in overweight men. British Journal of Nutrition. 2009;101(3):383-90.

105. Rufer CE, Moeseneder J, Briviba K, Rechkemmer G, Bub A. Bioavailability of astaxanthin stereoisomers from wild (Oncorhynchus spp.) and aquacultured (Salmo salar) salmon in healthy men: a randomised, double-blind study. British Journal of Nutrition. 2008;99(5):1048-54.

106. Din JNH, S. A. Valerio, C. J. Sarma, J. Lyall, K. Riemersma, R. A. Newby, D. E. Flapan, A. D. Dietary intervention with oil rich fish reduces platelet-monocyte aggregation in man. Atherosclerosis. 2008;197(1):290-6.

107. Rajaram S, Haddad EH, Mejia A, Sabaté J. Walnuts and fatty fish influence different serum lipid fractions in normal to mildly hyperlipidemic individuals: A randomized controlled study. American Journal of Clinical Nutrition. 2009;89(5):1657S-63S.

108. Chiang YL, Haddad E, Rajaram S, Shavlik D, Sabaté J. The effect of dietary walnuts compared to fatty fish on eicosanoids, cytokines, soluble endothelial adhesion molecules and lymphocyte subsets: A randomized, controlled crossover trial. Prostaglandins Leukotrienes and Essential Fatty Acids. 2012;87(4-5):111-7.

109. Lloyd AJF, G. Beckmann, M. Lin, W. Tailliart, K. Xie, L. Mathers, J. C. Draper, J. Use of mass spectrometry fingerprinting to identify urinary metabolites after consumption of specific foods. American Journal of Clinical Nutrition. 2011;94(4):981-91.

110. Meydani SN, Lichtenstein AH, Cornwall S, Meydani M, Goldin BR, Rasmussen H, et al. Immunologic effects of national cholesterol education panel step-2 diets with and without fish-derived N-3 fatty acid enrichment. Journal of Clinical Investigation. 1993;92(1):105-13.

111. Raatz SK, Rosenberger TA, Johnson LK, Wolters WW, Burr GS, Picklo MJ. Dose-Dependent Consumption of Farmed Atlantic Salmon (Salmo salar) Increases Plasma Phospholipid n-3 Fatty Acids Differentially. Journal of the Academy of Nutrition and Dietetics. 2013;113(2):282-7.

112. Lindqvist H, Langkilde AM, Undeland I, Radendal T, Sandberg AS. Herring (Clupea harengus) supplemented diet influences risk factors for CVD in overweight subjects. European Journal of Clinical Nutrition. 2007;61(9):1106-13.

113. Lloyd AJ, Favé G, Beckmann M, Lin W, Tailliart K, Xie L, et al. Use of mass spectrometry fingerprinting to identify urinary metabolites after consumption of specific foods. The American Journal of Clinical Nutrition. 2011;94(4):981-91.

114. Andersen MBS, Reinbach HC, Rinnan A, Barri T, Mithril C, Dragsted LO. Discovery of exposure markers in urine for Brassica-containing meals served with different protein sources by UPLC-qTOF-MS untargeted metabolomics. Metabolomics. 2013;9(5):984-97.

115. Stanstrup J, Schou SS, Holmer-Jensen J, Hermansen K, Dragsted LO. Whey protein delays gastric emptying and suppresses plasma fatty acids and their metabolites compared to casein, gluten, and fish protein. J Proteome Res. 2014;13(5):2396-408.

116. Mann N, Sinclair A, Pille M, Johnson L, Warrick G, Reder E, et al. The effect of short-term diets rich in fish, red meat, or white meat on thromboxane and prostacyclin synthesis in humans. Lipids. 1997;32(6):635-44.

117. Shiokawa Y, Misawa T, Date Y, Kikuchi J. Application of Market Basket Analysis for the Visualization of Transaction Data Based on Human Lifestyle and Spectroscopic Measurements. Analytical Chemistry. 2016;88(5):2714-9.

118. Heinzmann SS, Merrifield CA, Rezzi S, Kochhar S, Lindon JC, Holmes E, et al. Stability and Robustness of Human Metabolic Phenotypes in Response to Sequential Food Challenges. Journal of Proteome Research. 2012;11(2):643-55.

119. Zhang AQ, Mitchell SC, Smith RL. Dietary precursors of trimethylamine in man: A pilot study. Food and Chemical Toxicology. 1999;37(5):515-20. doi: 10.1016/S0278-6915(99)00028-9.

120. Lindberg M, Midthjell K, Bjerve KS. Long-term tracking of plasma phospholipid fatty acid concentrations and their correlation with the dietary intake of marine foods in newly diagnosed diabetic patients: results from a follow-up of the HUNT Study, Norway. British Journal of Nutrition. 2013;109(6):1123-34.

121. Hautero U, Poussa T, Laitinen K. Simple dietary criteria to improve serum n-3 fatty acid levels of mothers and their infants. Public health nutrition. 2017;20(3):534-41.

122. Saadatian-Elahi M, Slimani N, Chajès V, Jenab M, Goudable J, Biessy C, et al. Plasma phospholipid fatty acid profiles and their association with food intakes: Results from a cross-sectional study within the European Prospective Investigation into Cancer and Nutrition. American Journal of Clinical Nutrition. 2009;89(1):331-46.

123. Nash SH, Kristal AR, Boyer BB, King IB, Metzgar JS, O'Brien DM. Relation between stable isotope ratios in human red blood cells and hair: implications for using the nitrogen isotope ratio of hair as a biomarker of eicosapentaenoic acid and docosahexaenoic acid. The American Journal of Clinical Nutrition. 2009;90(6):1642-7.

124. O'Brien DM, Kristal AR, Jeannet MA, Wilkinson MJ, Bersamin A, Luick B. Red blood cell delta(15)N: a novel biomarker of dietary eicosapentaenoic acid and docosahexaenoic acid intake. American Journal of Clinical Nutrition. 2009;89(3):913-9.

125. Lu Y, Zou L, Su J, Tai ES, Whitton C, Dam RMV, et al. Meat and Seafood Consumption in Relation to Plasma Metabolic Profiles in a Chinese Population: A Combined Untargeted and Targeted Metabolomics Study. Nutrients. 2017;9(7).

126. Nash SH, Bersamin A, Kristal AR, Hopkins SE, Church RS, Pasker RL, et al. Stable Nitrogen and Carbon Isotope Ratios Indicate Traditional and Market Food Intake in an Indigenous Circumpolar Population. The Journal of Nutrition. 2012;142(1):84-90.

127. O'Gorman A, Morris C, Ryan M, O'Grada CM, Roche HM, Gibney ER, et al. Habitual dietary intake impacts on the lipidomic profile. Journal of Chromatography B: Analytical Technologies in the Biomedical and Life Sciences. 2014;966:140-6.

128. Lloyd AJ, Beckmann M, Haldar S, Seal C, Brandt K, Draper J. Data-driven strategy for the discovery of potential urinary biomarkers of habitual dietary exposure. The American Journal of Clinical Nutrition. 2013;97(2):377-89.

129. Seierstad SL, Seljeflot I, Johansen O, Hansen R, Haugen M, Rosenlund G, et al. Dietary intake of differently fed salmon; the influence on markers of human atherosclerosis. European Journal of Clinical Investigation. 2005;35(1):52-9..

130. Djuricic ID, Mazic SD, Kotur-Stevuljevic JM, Djordjevic VR, Sobajic SS. Long-chain n-3 polyunsaturated fatty acid dietary recommendations are moderately efficient in optimizing their status in healthy middle-aged subjects with low fish consumption: A cross-over study. Nutrition Research. 2014;34(3):210-8.

131. Brown AJ, Pang E, Roberts DCK. ERYTHROCYTE EICOSAPENTAENOIC ACID VERSUS DOCOSAHEXAENOIC ACID AS A MARKER FOR FISH AND FISH OIL CONSUMPTION. Prostaglandins Leukotrienes and Essential Fatty Acids. 1991;44(2):103-6.

132. Fukuoka Y, Nuruki N, Amiya S, Tofuku K, Aosaki S, Tsubouchi H. Effects of a fish-based diet and administration of pure eicosapentaenoic acid on brachial-ankle pulse wave velocity in patients with cardiovascular risk factors. Journal of Cardiology. 2014;63(3):211-7.

133. Wu JH, Marchioli R, Silletta MG, Macchia A, Song X, Siscovick DS, et al. Plasma phospholipid omega-3 fatty acids and incidence of postoperative atrial fibrillation in the OPERA trial. Journal of the American Heart Association. 2013;2(5).

134. Hodson L, Crowe FL, McLachlan KJ, Skeaff CM. Effect of supplementation with flaxseed oil and different doses of fish oil for 2 weeks on plasma phosphatidylcholine fatty acids in young women. European journal of clinical nutrition. 2018;72(6):832-40.

135. Fujioka S, Hamazaki K, Itomura M, Huan M, Nishizawa H, Sawazaki S, et al. The effects of eicosapentaenoic acid-fortified food on inflammatory markers in healthy subjects - A randomized, placebo-controlled, double-blind study. Journal of Nutritional Science and Vitaminology. 2006;52(4):261-5.

136. Browning LM, Walker CG, Mander AP, West AL, Madden J, Gambell JM, et al. Incorporation of eicosapentaenoic and docosahexaenoic acids into lipid pools when given as supplements providing doses equivalent to typical intakes of oily fish. American Journal of Clinical Nutrition. 2012;96(4):748-58.

137. Soyland E, Funk J, Rajka G, Sandberg M, Thune P, Rustad L, et al. EFFECT OF DIETARY SUPPLEMENTATION WITH VERY-LONG-CHAIN N-3 FATTY-ACIDS IN PATIENTS WITH PSORIASIS. New England Journal of Medicine. 1993;328(25):1812-6.

138. Flock MR, Skulas-Ray AC, Harris WS, Etherton TD, Fleming JA, Kris-Etherton PM. Determinants of Erythrocyte Omega-3 Fatty Acid Content in Response to Fish Oil Supplementation: A Dose-Response Randomized Controlled Trial. Journal of the American Heart Association. 2013;2(6).

139. Flock MR, Skulas-Ray AC, Harris WS, Gaugler TL, Fleming JA, Kris-Etherton PM. Effects of supplemental long-chain omega-3 fatty acids and erythrocyte membrane fatty acid content on circulating inflammatory markers in a randomized controlled trial of healthy adults. Prostaglandins, leukotrienes, and essential fatty acids. 2014;91(4):161-8.

140. Ulven SM, Kirkhus B, Lamglait A, Basu S, Elind E, Haider T, et al. Metabolic Effects of Krill Oil are Essentially Similar to Those of Fish Oil but at Lower Dose of EPA and DHA, in Healthy Volunteers. Lipids. 2011;46(1):37-46.

141. Kaul N, Kreml R, Austria JA, Richard MN, Edel AL, Dibrov E, et al. A comparison of fish oil, flaxseed oil and hempseed oil supplementation on selected parameters of cardiovascular health in healthy volunteers. Journal of the American College of Nutrition. 2008;27(1):51-8.

142. Wu SY, Mayneris-Perxachs J, Lovegrove JA, Todd S, Yaqoob P. Fish-oil supplementation alters numbers of circulating endothelial progenitor cells and microparticles independently of eNOS genotype. American Journal of Clinical Nutrition. 2014;100(5):1232-43.

143. Beblo S, Reinhardt H, Demmelmair H, Muntau AC, Koletzko B. Effect of Fish Oil Supplementation on Fatty Acid Status, Coordination, and Fine Motor Skills in Children with Phenylketonuria. Journal of Pediatrics. 2007;150(5):479-84.

144. Barceló-Coblijn G, Murphy EJ, Othman R, Moghadasian MH, Kashour T, Friel JK. Flaxseed oil and fish-oil capsule consumption alters human red blood cell n-3 fatty acid composition: A multiple-dosing trial comparing 2 sources of n-3 fatty acid. American Journal of Clinical Nutrition. 2008;88(3):801-9.

145. Oelrich B, Dewell A, Gardner CD. Effect of fish oil supplementation on serum triglycerides, LDL cholesterol and LDL subfractions in hypertriglyceridemic adults. Nutrition, Metabolism and Cardiovascular Diseases. 2013;23(4):350-7.

146. Katan MB, Deslypere JP, vanBirgelen A, Penders M, Zegwaard M. Kinetics of the incorporation of dietary fatty acids into serum cholesteryl esters, erythrocyte membranes, and adipose tissue: an 18-month controlled study. Journal of Lipid Research. 1997;38(10):2012-22.

147. Zheng JS, Lin M, Imamura F, Cai WW, Wang L, Feng JP, et al. Serum metabolomics profiles in response to n-3 fatty acids in Chinese patients with type 2 diabetes: a double-blind randomised controlled trial. Scientific Reports. 2016;6.

148. Gammelmark A, Madsen T, Varming K, Lundbye-Christensen S, Schmidt EB. Low-dose fish oil supplementation increases serum adiponectin without affecting inflammatory markers in overweight subjects. Nutrition Research. 2012;32(1):15-23.

149. Root M, Collier SR, Zwetsloot KA, West KL, McGinn MC. A randomized trial of fish oil omega-3 fatty acids on arterial health, inflammation, and metabolic syndrome in a young healthy population. Nutrition Journal. 2013;12(1).

150. Wallace FA, Miles EA, Calder PC. Comparison of the effects. of linseed oil and different doses of fish oil on mononuclear cell function in healthy human subjects. British Journal of Nutrition. 2003;89(5):679-89.

151. Huang T, Li K, Asimi S, Chen Q, Li D. Effect of vitamin B-12 and n-3 polyunsaturated fatty acids on plasma homocysteine, ferritin, C-reactive protein, and other cardiovascular risk factors: A randomized controlled trial. Asia Pacific Journal of Clinical Nutrition. 2015;24(3):403-11.

152. Harris WS, Rambjør GS, Windsor SL, Diederich D. n-3 fatty acids and urinary excretion of nitric oxide metabolites in humans. American Journal of Clinical Nutrition. 1997;65(2):459-64.

153. Wahl HG, Tetschner B, Liebich HM. The Effect of Dietary Fish Oil Supplementation on the Concentration of 3-Carboxy-4-Methyl-5-Propyl-2-Furanpropionic Acid in Human Blood and Urine. Hrc-J High Res Chrom. 1992;15(12):815-8.

154. Saifullah A, Watkins BA, Saha C, Li Y, Moe SM, Friedman AN. Oral fish oil supplementation raises blood omega-3 levels and lowers C-reactive protein in haemodialysis patients- a pilot study. Nephrology Dialysis Transplantation. 2007;22(12):3561-7.

155. Patterson AC, Chalil A, Henao JJA, Streit IT, Stark KD. Omega-3 polyunsaturated fatty acid blood biomarkers increase linearly in men and women after tightly controlled intakes of 0.25, 0.5, and 1 g/d of EPA plus DHA. Nutrition Research. 2015;35(12):1040-51.

156. Saldeen T, Wallin R, Marklinder I. Effects of a small dose of stable fish oil substituted for margarine in bread on plasma phospholipid fatty acids and serum triglycerides. Nutrition Research. 1998;18(9):1483-92.

157. Hanwell HEC, Kay CD, Lampe JW, Holub BJ, Duncan AM. Acute Fish Oil and Soy Isoflavone Supplementation Increase Postprandial Serum (n-3) Polyunsaturated Fatty Acids and Isoflavones but Do Not Affect Triacylglycerols or Biomarkers of Oxidative Stress in Overweight and Obese Hypertriglyceridemic Men. Journal of Nutrition. 2009;139(6):1128-34.

158. Zabel R, Ash S, King N, Naslund E, Bauer J. Gender differences in the effect of fish oil on appetite, inflammation and nutritional status in haemodialysis patients. Journal of Human Nutrition and Dietetics. 2010;23(4):416-25.

159. Foulon T, Richard MJ, Payen N, Bourrain JL, Beani JC, Laporte F, et al. Effects of fish oil fatty acids on plasma lipids and lipoproteins and oxidant-antioxidant imbalance in healthy subjects. Scandinavian Journal of Clinical & Laboratory Investigation. 1999;59(4):239-48.

160. Roke K, Mutch DM. The role of FADS1/2 polymorphisms on cardiometabolic markers and fatty acid profiles in young adults consuming fish oil supplements. Nutrients. 2014;6(6):2290-304.

161. Zulyniak MA, Perreault M, Gerling C, Spriet LL, Mutch DM. Fish oil supplementation alters circulating eicosanoid concentrations in young healthy men. Metabolism-Clinical and Experimental. 2013;62(8):1107-13.

162. Hals J, Bjerve KS, Nilsen H, Svalastog AG, Ek J. Essential fatty acids in the nutrition of severely neurologically disabled children. British Journal of Nutrition. 2000;83(3):219-25.

163. Von Schacky C, Fischer S, Weber PC. Long-term effects of dietary marine ω-3 fatty acids upon plasma and cellular lipids, platelet function, and eicosanoid formation in humans. Journal of Clinical Investigation. 1985;76(4):1626-31.

164. Molin MU, S. M. Dahl, L. Telle-Hansen, V. H. Holck, M. Skjegstad, G. Ledsaak, O. Sloth, J. J. Goessler, W. Oshaug, A. Alexander, J. Fliegel, D. Ydersbond, T. A. Meltzer, H. M. Humans seem to produce arsenobetaine and dimethylarsinate after a bolus dose of seafood. Environmental Research. 2012;112:28-39.

165. Yun HY, Lampe JW, Tinker LF, Neuhouser ML, Beresford SAA, Niles KR, et al. Serum Nitrogen and Carbon Stable Isotope Ratios Meet Biomarker Criteria for Fish and Animal Protein Intake in a Controlled Feeding Study of a Women's Health Initiative Cohort. The Journal of nutrition. 2018;148(12):1931-7.

166. O'Dea K, Sinclair AJ. Increased proportion of arachidonic acid in plasma lipids after 2 weeks on a diet of tropical seafood. Am J Clin Nutr. 1982;36(5):868-72.

167. Heitland PK, H. D. Fast determination of arsenic species and total arsenic in urine by HPLC-ICP-MS: Concentration ranges for unexposed german inhabitants and clinical case studies. Journal of Analytical Toxicology. 2008;32(4):308-14.

168. Lovreglio PDE, M. N. De Pasquale, P. Gilberti, M. E. Drago, I. Panuzzo, L. Lepera, A. Serra, R. Ferrara, F. Basso, A. Apostoli, P. Soleo, L. Environmental factors affecting the urinary excretion of inorganic arsenic in the general population. Medicina del Lavoro. 2012;103(5):372-81.

169. Rivera-Núñez ZM, J. R.; Meeker, J. D.; Slotnick, M. J.; Nriagu, J. O. Urinary arsenic species, toenail arsenic, and arsenic intake estimates in a Michigan population with low levels of arsenic in drinking water. Journal of Exposure Science and Environmental Epidemiology. 2012;22(2):182-90.

170. Chung H, Nettleton JA, Lemaitre RN, Barr RG, Tsai MY, Tracy RP, et al. Frequency and Type of Seafood Consumed Influence Plasma (n-3) Fatty Acid Concentrations. Journal of Nutrition. 2008;138(12):2422-7.

171. Navas-Acien AS, E. K.; Pastor-Barriuso, R.; Guallar, E. Arsenic exposure and prevalence of type 2 diabetes in US adults. Jama-Journal of the American Medical Association. 2008;300(7):814-22.

172. Miyazawa T, Nakagawa K, Kimura F, Satoh A, Miyazawa T. Erythrocytes carotenoids after astaxanthin supplementation in middle-aged and senior Japanese subjects. Journal of oleo science. 2011;60(10):495-9.

173. Miyazawa T, Nakagawa K, Kimura F, Satoh A, Miyazawa T. Plasma carotenoid concentrations before and after supplementation with astaxanthin in middle-aged and senior subjects. Bioscience, biotechnology, and biochemistry. 2011;75(9):1856-8.

174. Childs MT, Dorsett CS, King IB, Ostrander JG, Yamanaka WK. Effects of shellfish consumption on lipoproteins in normolipidemic men. Am J Clin Nutr. 1990;51(6):1020-7..

175. Lai VWMS, Y.; Ting, E.; Cullen, W. R.; Reimer, K. J. Arsenic speciation in human urine: Are we all the same? Toxicology and applied pharmacology. 2004;198(3):297-306.
