# Supplementary material for: Biomarkers of meat and seafood intake: an extensive literature review
Source: Genes Nutr. 2019 Dec 30;14:35. doi: 10.1186/s12263-019-0656-4 (PMC6937850; doi:10.1186/s12263-019-0656-4)
Supplement: Supplementary file 3 — Additional file 3: Table S4. Summary of the selected candidate biomarkers of meat and seafood intake and the discarded markers. For each marker, a brief explanation for inclusion or exclusion from the list of candidate FIBs is reported. [file 12263_2019_656_MOESM3_ESM.pdf]

Table S4. Summary of the selected candidate biomarkers of meat and seafood intake and the discarded markers. For each marker, a brief explanation for inclusion or exclusion from the list of candidate FIBs is reported

**Food item**	**Metabolites**	**Biofluid Locations**	**Reason for inclusion or exclusion from the list of candidates FIBs**	**Selected for the systematic validation as FIB**
**Meat (all)**	δ15N + δ13C	Faeces	Candidate biomarker to assess recent intake of meat in populations with limited dairy and egg intakes. It has to be validated for each specific populations, as it is affected by other sources of animal proteins.	Yes
	δ15N + δ13C	Urine	Candidate biomarker to assess recent intake of meat. It has to be validated for each specific population, as it is affected by other sources of animal proteins.	Yes
	δ15N	Plasma/Serum	Candidate biomarker to assess the habitual intake of meat. It has to be validated for each specific population, as it is affected by other sources of animal proteins.	Yes
	δ15N + δ13C	Hair	Candidate biomarker to assess the habitual intake of meat. It has to be validated for each specific population, as it is affected by other sources of animal proteins.	Yes
	Anserine	Urine	Candidate marker of recent intake of meat. Levels are much higher in chicken and certain kinds of fish (salmon and tuna)	Yes
	Anserine	Plasma	Insufficient data for validation.	No
	3-Methylhistidine	Urine	Candidate marker to assess recent meat intake, particularly chicken, but also certain kinds of fish. Due to differences in 3-MH content in different animals, it needs to be validated for each study population for quantitative purpose.	Yes
	3-Methylhistidine	Plasma	Candidate marker to assess the consumption of meat proteins, especially for chicken or for poultry in general.	Yes
	1-Methylhistidine	Urine	Endogenous compound originating from muscle protein breakdown. High background level and inter-individual variation. May be used as part of combined markers.	No
	1-Methylhistidine	Plasma	Insufficient data for validation.	No
	β-alanine	Plasma	Endogenous metabolite. Not specific enough to assess meat protein intake.	No
	Carnitine and acylcarnitines.	Urine and serum	Endogenous metabolites increasing after meat intake. Other physiological conditions may affect the ability of these compounds to quantitatively estimate meat intake.	No
	Creatine	Urine	Present in muscle, including human resulting in some background. Candidate marker for short-term and habitual intake of meat and seafood. May be used as part of combined markers.	Yes

	Creatine	Blood	Present in muscle, including human. Putative marker for habitual intake of meat.	Yes
	Creatinine	Urine	Endogenous metabolite. Excretion into urine is highly depending on kidney function. Not suitable as FIB.	No
	Carnosine	Urine	Candidate marker to assess recent meat intake. Differences in the contents in different meats may preclude the use this marker for quantitative total meat intake assessment in individuals.	Yes
	Carnosine	Plasma	Discrepancies between studies. Further investigation is needed.	No
	1-methylhistidine + 3-methylhistidine + carnosine	Urine	Combination of marker to predict a meat-based diet against lacto-ovo vegetarian diet in a controlled setting. Better prediction than single markers. Need for further validation.	Yes
	Taurine		Originating from endogenous formation and from intake of animal tissues. Discrepancies between studies. Not robust enough to be used as FIB.	No
	Trans-4-hydroxyproline	Plasma	Present in connective tissues. Putative marker to estimate the overall intake of meat. Further studies are needed to evaluate the specificity of the marker in case of fish intake.	Yes
	Agmatine	Plasma	Potentially increased with meat. Agmatine is an intermediate metabolite in arginine catabolism and a neuroactive effector. As such it cannot serve as an intake biomarker but possibly as an effect marker.	No
	TMAO	Plasma	Depending on microbial and host characteristincs.	No
**Red meat**	Ferritin	Serum	Contradictory results exist about the relationship between red meat intake and serum ferritin. More investigation is needed to investigate dose-response relationships between serum ferritin and red meat consumption.	No
	ATNCs	Urine	Positive association between heme iron content and increasing doses of red meat. Possibly an artifact of analysis. Need for better validation studies.	No
	DHN-MA	Urine	Positive association with heme iron content and/or increasing doses of red meat. Probably unspecific. Need for better validation studies.	No
**Offal meat**	8-iso-PGF2A	Urine	Not specific as meat intake biomarker.	No
	DHN-MA	Urine	Not specific to offal meats	No
**Poultry**	Anserine	Urine	Candidate marker of recent intake of poultry but also found after intake of certain kinds of fish (salmon and tuna). Due to differences in anserine content in different meats, its use for intake assessment of poultry in individual needs to be validated for each population.	Yes

	3-MH	Urine	Candidate marker of poultry intake but present also after some other meats; its use is therefore dependent on other meat intakes.	Yes
	Guanidinoacetate	Urine	Candidate biomarkers of chicken intake. Needs further validation for other poultry including those not fed with the compound.	Yes
**Heated meat and seafood**	MeIQx (free)	Urine	Not detected	No
	PhIP (free)	Urine	High inter-individual variability.	No
	PhIP (free)	Faeces	High inter-individual variability. No dose-response relationship.	No
	PhIP (free)	Hair	Candidate markers of habitual consumption of fried and roasted meat. Need for validation in observational studies.	Yes
	MeIQx (total, after enzymatic hydrolysis)	Urine	Candidate marker to assess short-term intake of fried or grilled meat and seafood. High inter-individual variability. Additional studies are needed to validate the marker in real-life exposures.	Yes
	PhIP (total, after enzymatic hydrolysis)	Urine	Candidate marker to assess short-term intake of fried or grilled meat and seafood. High inter-individual variability. Additional studies are needed to validate the marker in real-life exposure.	Yes
	MeIQx metabolites	Urine	Too high inter-induvial variability	No
	PhIP metabolites	Urine	Too high inter-induvial variability	No
	4'-OH-PhIP	Urine	Candidate marker to assess the exposure to roasted meat	Yes
	5-OH-PhIP	Urine	Marker of activation of PhIP. Not suitable as FIB for heated meat intake.	No
	Other HAAs	Urine/ plasma	Too high inter-individual variability	No
	PhIP-M1	Faeces	Too high inter-individual variability	No
	PAHs	Urine	Not specific enough to assess the intake of grilled, broiled or roasted meat in real life exposure due to confounders (e.g. cigarette smoking, air pollution).	No
**Processed meat**	Membrane lipids.	Plasma/serum	Not specific for use as biomarker	No
	Nitrosoproline	Plasma	Not specific for use as biomarker	No
**Marine fish and other seafood**	δ15N + δ13C	Plasma	Candidate biomarker to assess the habitual intake of fish, only evaluated in observational studies.	No
	EPA (total)	Blood	Candidate intake biomarker for marine fats	Yes
	DHA (total)	Blood	Candidate intake biomarker for marine fats	Yes
	EPA free	plasma/serum	Candidate shorter-term intake biomarker for marine fats	Yes
	DHA free	plasma/serum	Candidate shorter-term intake biomarker for marine fats	Yes
	EPA esterified	Plasma PCs	Candidate shorter-term intake biomarker for marine fats	Yes

DHA esterified	Plasma PCs	Candidate shorter-term intake biomarker for marine fats.	Yes
EPA	RBC membranes	Candidate long-term intake biomarker for marine fats	Yes
DHA	RBC membranes	Candidate long-term intake biomarker for marine fats	Yes
EPA/AA	Plasma phospholipids	Putative marker to differentiate fish intake from meat intake in controlled settings only.	Yes
n-6/n-3 LCPUFAs	Plasma	Putative marker to differentiate fish intake from meat intake in controlled settings only.	Yes
CMPF	Urine	Candidate marker to assess habitual fatty fish intake in free living populations.	Yes
CMPF	Plasma	Candidate marker to assess habitual fatty fish intake in free living populations	Yes
TMAO	Urine	Candidate marker to assess recent fish intake with high sensitivity.	Yes
THCC	Plasma/urine	Not specific for use as biomarker	No
Phosphatidylethanolamine	Plasma	Not specific for use as biomarker	No
Cetoleic acid	Plasma	Not specific for use as biomarker	No
Astaxanthin	Plasma	Candidate biomarker of salmon and red seafood intake	Yes
Arsenobetaine	Urine	Candidate biomarker to assess habitual or recent intake of seafood. May be affected by geographical and individual factors. Needs further validation.	Yes
Arsenobetaine	Plasma	Candidate biomarker to assess short-term intake of seafood. May be affected by geographical and individual factors. Needs further validation.	Yes
